# Investigation of the effects of *Periplaneta americana* (L.) extract on ischemic stroke based on combined multi-omics of gut microbiota

**DOI:** 10.3389/fphar.2024.1429960

**Published:** 2024-11-28

**Authors:** Xin Yang, Canhui Hong, Tangfei Guan, ChengGui Zhang, Peiyun Xiao, Yongshou Yang, Huai Xiao, Zhengchun He

**Affiliations:** ^1^ Yunnan Provincial Key Laboratory of Entomological Biopharmaceutical R&D, College of Pharmacy, Dali University, Dali, Yunnan, China; ^2^ National-Local Joint Engineering Research Center of Entomoceutics, Dali, Yunnan, China; ^3^ West China School of Public Health and West China Fourth Hospital, Sichuan University, Sichuan, Chengdu, China

**Keywords:** *Periplaneta americana* (L.), ischemic stroke, gut-brain axis, gut microbiota, metabolomics, transcriptomics

## Abstract

Ischemic stroke (IS) is a highly lethal type of cardiovascular and cerebrovascular disease. Improving survival rates and promoting recovery in patients with IS pose significant challenges, however, recent research has identified the gut–brain axis as a therapeutic target. In this study, we evaluated the regulatory effect of *Periplaneta americana* (L.) extract (PAS840), which has established anti-inflammatory, antioxidant, and neuroprotective effects, on the gut microbiota using a rat model of temporary middle cerebral artery occlusion (tMCAO). We evaluated the protective effects of PAS840 on brain damage in IS rats through TTC (triphenyltetrazolium chloride), Nissl staining, and pathological section analysis. Additionally, we investigated the impact of PAS840 on the gut microbiota and metabolites using 16S rRNA sequencing, untargeted metabolomics of gut contents, and transcriptomics analyses of brain tissues to explore its mechanism of action. PAS840 intervention resulted in significant changes in the gut microbiota, including an increase in the abundance of probiotic flora, decrease in the abundance of harmful flora, and significant changes in metabolite profiles. It also attenuated brain damage, decreased platelet activity, inhibited oxidative stress and genes related to inflammation, and improved neurological function in rats. These findings suggest that PAS840 has preventive and therapeutic effects against IS via the gut–brain axis by regulating the gut microbiota and related metabolites. Accordingly, PAS840 is a candidate therapeutic drug for further research.

## 1 Introduction

Stroke is a highly prevalent cardiovascular and cerebrovascular disease that manifests as ischemic stroke (IS) and hemorrhagic stroke, with IS accounting for 70% of all reported cases (Liu et al., 2019; Feigin et al., 2022). The pathogenesis of IS is complex and involves a robust inflammatory response and oxidative stress accompanied by various complications (Anrather and Iadecola, 2016). Modern treatments for IS are primarily based on pharmacological and mechanical thrombolysis to restore blood supply; however, because of their narrow therapeutic window, these approaches are accompanied by severe reperfusion injury (Eltzschig and Eckle, 2011), postoperative neurological impairments, such as limb paralysis, cognitive impairment, and depression (Jyotirekha and Rajanikant, 2018). Therefore, it is crucial to identify safe and effective drugs to prevent or treat IS, mitigate IS-induced injuries, and reduce complications. The gut-brain axis bidirectional regulation theory suggests that gut microbiota plays a crucial role in cerebrovascular and nervous system diseases and can bi-directionally regulate brain function and maintain brain health from multiple perspectives (Mayer et al., 2022; Mu et al., 2016). Changes in gut microbiota and microbiota metabolites are closely related to intestinal function, thrombosis, and cardiovascular and cerebrovascular diseases (Zhu et al., 2016). Therefore, treating cardiovascular and cerebrovascular diseases by altering the gut microbiota metabolites and intestinal barrier function is a valuable strategy in the treatment and prevention of IS.

*Periplaneta americana* (L.) (PA) is an insect of the genus Cockroach in the family Periplaneta, which was first documented as a traditional Chinese medicinal insect in 25–220 A.D. by Shennong Ben Cao (Zeng et al., 2019). In southwest China, various ethnic minority groups, including Dong, Yi, and Hani, commonly employ it for the treatment of internal and external injuries, dysentery, inflammation, edema, and other ailments (Minru and Yi, 2016; Bai and Liu, 2020). Modern pharmacological studies have shown that *Periplaneta americana* (L.) extract (PAE) has excellent anti-inflammatory, antibacterial, antioxidant, tissue repairing, and neuroprotective effects (Liu et al., 2022; Zhou et al., 2023). PAE is rich in amino acids, peptides, nucleosides, polysaccharides, and fatty acids (Li et al., 2018; Nguyen et al., 2019). Several studies have shown that PAE exerts significant therapeutic effects against viral hepatitis, steatohepatitis, colitis, gastric ulcers, and skin tissue damage (Fu et al., 2021; Li et al., 2022; Lu et al., 2022; Xiao et al., 2022; Zhu et al., 2018). In addition, the existing clinical preparation, “Xinmai Long” injection, which is made of PAE, has good therapeutic effects on cardiovascular diseases (Lin et al., 2019; Zhang et al., 2021b). Studies have shown that PAE activates the extracellular signal-regulated kinase (ERK)/cAMP response element binding protein (CREB)/brain-derived neurotrophic factor (BDNF) signaling pathway. This protects brain cells after a stroke and accelerates the recovery of brain function (Rao et al., 2023). Another related study showed that PAE can inhibit harmful microbiome in the intestinal tract, regulate the abundance of gut microbiota, and alleviate the stimulatory effect of intestinal diseases (Yoon et al., 2017), thereby improving anxiety and depressive behaviors in rats (Liu et al., 2023). Therefore, we speculate that PAE may be effective in preventing and treating IS by adjusting the gut microbiota, ameliorating intestinal oxidative stress and inflammatory response caused by external stimuli, and exerting a regulatory effect on the gut-brain axis.

Therefore, in this study, based on the theory of gut microbiota-gut-brain axis regulation, we used a rat model of temporary middle cerebral artery occlusion (tMCAO) to evaluate the efficacy of PAE (PAS840) against IS based on brain pathology characteristics. In addition, we used 16S rRNA sequencing and untargeted metabolomics analyses to reveal the relationship between the composition of the gut microbiota and its metabolites, in addition to a transcriptomics analysis of brain tissues to elucidate the mechanism underlying the preventive and therapeutic effects of PAS840 in IS. This study provides a scientific and theoretical basis for the role of PAS840 in preventing and controlling the development of IS.

## 2 Methods

### 2.1 Use of drugs

PAS840 is the crushed material from the dried body of PA, extracted using 90% ethanol at five times its weight. The extract was concentrated and defatted. The defatted extracts were adsorbed using S-8 macroporous adsorbent resin columns. The resin column was then washed with water and eluted with 40% ethanol. The eluate was collected, concentrated under reduced pressure at 60°C, and freeze-dried to obtain the PAS840 sample.

NaoXinTong capsule (NXT), a proprietary Chinese medicine used clinically for IS treatment, was purchased from Shaanxi Buchang Pharmaceutical Co. Ltd (Shaanxi, China).

### 2.2 Materials

The following reagents and chemicals were used in the experiment: Ammonium bicarbonate (A6141-500G, Sigma-Aldrich, United States), triethylammonium bicarbonate buffer (TEAB) (T7408-100 mL, Sigma-Aldrich, United States), urea (M123-1 KG, Amresco, United States), protein quantification stain (HXJ5137, Huaxingbio, China), bovine serum albumin (23,209, Thermo Scientific, United States), methanol (67–56–1,Thermo, China), 2-chloro-L-phenylalanine (103,616–89-3, Aladdin, China), dithiothreitol DTT (M109-5G, Amresco, United States), anhydrous ethanol/xylene/glacial acetic acid/hydrochloric acid (100,092,683/10,023,418/G10000218/10,011,008, Sinopharm, China), iodoacetamide IAM (M216-30G, Amresco, United States), ammonium formate (540–69-2, Sigma, Germany), trypsin (V5280/100ug, Promega, United States), Ziptip (ZTC18M096, Millipore, United States), acetonitrile (34,851 MSDS, J.T. Baker, United States), ammonia (013–23355, Wako, Japan), formic acid (T79708, Sigma-Aldrich, United States), injection bottle (11,190,533, Thermo, China), cap (11,150,635, Thermo, China), triphenyltetrazolium chloride (TTC) stain (L2128203, Aladdin, China), diluent for veterinary hemocyte analysis/hemolysate for veterinary hemocyte analysis (2,021,050,802/2,021,042,301, Mindray, China), Neutral Gum/HE Stain/Nissl dye solution (B0044/BHO001/B0013, Wuhan Bolf Biotechnology Co., Ltd., China), Isoflurane (2,023,090,502, Shandong Ante Herding Technology Co., Ltd., China), NEBNext Ultra II RNA Library Prep Kit for Illumina (New England Biolabs Inc., United States), Kit for Illumina (New England Biolabs Inc., United States), malondialdehyde (MDA)/superoxide dismutase (SOD) Assay Kits (20,231,006/20,231,018, Nanjing Jiancheng Bioengineering Institute, China), and MCAO line bolus/bupivacaine hydrochloride (20,230,506, Beijing Sinonon Biotechnology Co., China).

### 2.3 Component analysis of PAS840

Appropriate PAS840 sample was takenand analyzed using Liquid chromatography-MS/MS(LC-MS/MS), then weighed and vortexed for 30 s in 600 µL of methanol containing 4-chloro-L-phenylalanine (4 ppm). A tissue grinder (MB-96; United States Wall) was used to ground the samples for 120 s at 50 Hz. Subsequently, the samples were centrifuged for 10 min at 12,000 rpm after sonication at 24°C ± 2°C. The supernatant was filtered through a 0.22 μm filter membrane. Finally, the filtrate was introduced into the test bottle for LC-MS analysis.

The ACQUITY UPLC^®^ HSS T3 (2.1 × 100 mm, 1.8 µm) (Waters, Milford, MA, United States) column was used for the analysis of the samples on a Thermo Vanquish (Thermo Fisher Scientific, United States) ultra-high-performance liquid chromatography (UHPLC) system. The column temperature was set at 40 °C, a flow rate of 0.3 mL/min, and an injection volume of 2 μL. The positive ionization mode used 0.1% formic acid in acetonitrile (B2) and 0.1% formic acid in water (A2) as mobile phases, the gradient elution program was: 0–1 min, 8% B2; 1–8 min, 8%–98% B2; 8–10 min, 98% B2; 10–10.1 min, 98% to 8%B2; 10.1–12 min, 8% B2. And the negative ionization mode used acetonitrile (B3) and 5 mM ammonium formate in water (A3) as mobile phases, the gradient elution program was: 0–1 min, 8% B3; 1–8 min, 8%–98% B3; 8–10 min, 98% B3; 10–10.1 min, 98%–8% B3; 10.1–12 min, 8% B3. A Thermo Q Exactive Mass Spectrometry Detector (Thermo Fisher Scientific, United States) equipped with an electrospray ionization (ESI) source in the positive and negative ion modes was used to collect data separately. The auxiliary gas, sheath gas, and positive ion spray voltage were 10 arb, 40 arb, and 3.50 kV, respectively. The negative ion spray voltage was −2.50 kV. The main ion scanning range was m/z 100–1,000, the capillary temperature was 325°C, and a resolution of 70,000 was achieved during the primary complete scan. Secondary cleavage was performed using HCD with a collision energy of 30 eV and a secondary resolution of 17,500. The first ten ions obtained were broken, and any extraneous MS/MS data were eliminated using dynamic exclusion.

### 2.4 IS animal model establishment

#### 2.4.1 Animal grouping and drug delivery

A total of 45 male Sprague-Dawley (SD) rats weighing 270 ± 20 g were acquired from Sipeifu Biotechnology Co. Ltd (Beijing, China) under license number SCXK 2019–0,010. SPF-grade-raising conditions were maintained. The rats were kept in a tightly regulated environment with a 12-h light/dark cycle, a temperature of 24°C ± 2°C, and a relative humidity of 65% ± 5%. Each rat was given ^60^Co irradiated sterilized maintenance rat food and purified water daily. The use of animals in this study was approved by the Laboratory Animal Ethics Committee of Dali University (grant no. 2024-PZ-001), and were performed according to the EU Directive 2010/63/EU for animal experiments.

The rats were randomly and equally split into five groups of 9 rats each: the Sham (sham surgery), the Model, the PAS840-low dosage (gavage: 30 mg/kg), the PAS840-high dosage (gavage: 120 mg/kg), and the NXT positive control (gavage: 81.25 mg/kg),NXT was used for pharmacodynamic comparison, which has been demonstrated to inhibit thrombosis and exhibits a positive therapeutic impact on stroke (Wang B. et al., 2021). Following 7 days of adaptive feeding, to establish a stable gut microbiota, all dosing was administered at the same time daily for 30 days prior to modeling, and the model and dummy groups received the same amount of saline during the test period. One dose was administered 12 h prior to tMCAO surgical modeling, and the medication was continued for 7 days afterwards.

#### 2.4.2 tMCAO model establishment

A rat tMCAO model was created using a suture-occlusion method to obstruct the right middle cerebral artery occlusion (MCAO). The right common carotid artery (CCA), external carotid artery (ECA), and internal carotid artery (ICA) were exposed through a midline neck incision during the experiment. The induction anesthesia was initiated using 4% isoflurane and maintained with a continuous inhalation anesthesia of 1.75%. Following anesthesia, the ECA was cut, and the MCAO monofilaments was inserted through the ECA to a depth of (18 ± 1) mm of ICA. After suturing the wound, the MCAO monofilaments was removed after 90 min. Only the skin was cut in the Sham group, and the blood vessels were divided before suturing. After surgery, bupivacaine hydrochloride was administered to all the rats to reduce pain.

### 2.5 Validation of indicators

#### 2.5.1 Neurological function measurement

After 24 h of modeling, neurological function scores (n = 9) were administered to each group of rats using the Zea Longa scoring method (Longa et al., 1989). The scoring system assigned the following values: 4 points for total paralysis, 3 points for paralyzed on one side, 2 points for walking using the paralyzed side, 1 point for body twisting and the inability to extend the front paw by lifting the tail, 0 points for no behavioral deficits, and ≥1 indicates that nerve function injury is inflicted and that modeling is successful.

#### 2.5.2 Infarction rate

The brain tissues of rats (n = 3) were separated and stained with TTC staining solution. After freezing for 20 min at −20°C, whole brain tissues were cut into five identical slices, each measuring approximately 2 mm in thickness. The brain slices were stained with a 4% TTC staining solution for 20 min at 37°C in a dark, protected environment. The infarction rate was computed using Image Pro Plus 2.0.

Infarction rate=total area of brain tissue that has been infarcted/ total area of the brain tissue×100%

#### 2.5.3 Pathological analysis

Brains (n = 3) were obtained from each group, and paraffin sections were prepared. Paraffin sections were baked, dewaxed, hydrated, stained with hematoxylin and eosin (HE), dehydrated, made transparent, and sealed. Subsequently, the pathomorphological changes in the cortical portion of the diseased brain tissue were observed under a microscope.

#### 2.5.4 Nissl body staining

Using HE-stained wax blocks of the same brain tissue, paraffin sections were baked, dewaxed, hydrated with Nissl dye solution for staining, dehydrated, treated with xylene, sealed, and microscopically observed for Nissl bodies in the affected cortical area of the brain tissue. The Nissl bodies were counted using Photoshop 2021 software (200 × magnification).

#### 2.5.5 Platelet count

A 20 μL of rat plasma was mixed with 5 mL of special diluent (n = 9), and the resulting platelet data (PLT) were recorded and counted after detection using a veterinary blood cell analyzer (Shenzhen Mindray Biomedical Co., Ltd., China).

#### 2.5.6 Biochemical indicators testing

Rat serum samples (n = 6) were obtained by centrifuging rat plasma for 10 min at 3,000 rpm and separating the supernatant. Subsequently, SOD activity and MDA concentration were determined. All procedures were performed according to the manufacturer’s instructions.

### 2.6 16S rRNA sequencing

Fresh feces collected from rats in the Sham (Pre-operation, ShamP) and PAS840H (Pre-operation, PAS840P) groups prior to modeling, and fresh feces from rats in the Sham, Model, and PAS840 H groups (PAS840) 6 h after the last administration of the last drug after modeling were subjected to 16S rRNA sequencing (n = 3). Total DNA from the feces of each group was extracted according to the instructions provided in the fecal genomic DNA extraction kit. A nanodrop was used to quantify the extracted DNA, and 1.2% agarose gel electrophoresis was performed to determine the quality of the extracted DNA. The 16S rRNA V3–V4 region of the target gene was amplified by PCR, and the resultant products were subjected to 2% agarose gel electrophoresis. Each sample was combined according to the corresponding ratio based on the sequencing volume required for each sample, as determined from the fluorescence quantification data. After the final purification, sequencing libraries were created using the Illumina TruSeq Nano DNA LT Library Prep Kit and subjected to high-throughput sequencing and examined using the QIIME2 (2019.4) software to determine their composition and abundance at the phylum, class, order, family, and genus levels. The R programming language and QIIME2 (2019.4) were used for Alpha and Beta diversity index analyses. The bacterial community interaction relationship network was plotted using Cytoscape 3.9.1 software, and metabolic pathway abundance values were determined according to the Kyoto Encyclopedia of Genes and Genomes (KEGG) metabolic pathway database.

### 2.7 Untargeted metabolomics assay for gut microbiota metabolites

Rats’ colonic contents (n = 6) from the Model, Sham, and PAS840H groups were gathered, the LC-MS/MS analytical techniques employed were identical to those used in Section 2.3. The peaks obtained from all the experimental and QC samples were analyzed using principal component analysis (PCA), partial least squares-discriminant analysis (PLS-DA), and orthogonal partial least squares-discriminant analysis (OPLS-DA). Next, the stability of the instrument, reproducibility of the experiments, and reliability of the data quality were comprehensively evaluated. Finally, the correlation of the differential substances was analyzed, and KEGG metabolic pathway analysis was used to determine the participation of differential metabolites in the *in vivo* metabolic process.

### 2.8 Gut microbiota and untargeted metabolomics correlation analysis

The Spearman rank correlation coefficient between the untargeted metabolomics data of bacterial metabolites and abundance was computed using the Mothur software, and a correlation heat map was created.

### 2.9 Brain tissue transcriptomics sequencing

Rat brain tissues (n = 3) from the Model, Sham, and PAS840 H groups were evaluated for total RNA quality. RNA-specific agarose electrophoresis was used to assess the extraction quality of the RNA. A sample of ≥1 µg of total RNA was chosen. The instructions in the NEBNext Ultra II RNA Library Prep Kit for Illumina were followed in the selection and processing of total RNA. Random oligonucleotides were used as primers to create cDNA, and the fragmented mRNA served as a template. Double-stranded cDNA was used to purify the cDNA, and when the double ends were fixed, the sequencing junction was joined, and the “A” base was added at the 3′end. To create the final library, cDNA was screened in the 400–500 bp range using AMPure XP beads, and the PCR products were purified using the same beads. The multiplexed DNA libraries were combined and homogenized into equal parts. The combined libraries were then gradually diluted, measured, and sequenced on an Illumina sequencer in the PE150 mode.

The samples were sequenced, and the software included in the sequencing equipment converted the image files to produce raw FASTQ data. HISAT2 (v2.1.0) was used to create the reference genome index and compare the clean paired-end reads to the reference genome. HTSeq (v0.9.1) was used to calculate the read count for each gene. DESeq (v1.38.3) was used to perform a differential expression analysis of the two combinations. The GO enrichment study was carried out using topGO, the *P*-value was computed using the hypergeometric distribution method after the gene list, and counts for each phrase were obtained using GO word-annotated differential genes. Using KEGG pathway-annotated differential genes, the gene list and counts for each pathway were tallied as part of the KEGG enrichment study carried out with ClusterProfiler. After calculating the gene list and gene number for every pathway, the hypergeometric distribution method was used to obtain the *P*-value (a *P*-value of less than 0.05 was considered significant enrichment).

### 2.10 Statistical analysis

The Parsonalbio GenesCloud Platform (https://www.genescloud.cn/cloudClassroom) and SPSS 27 were used to analyze all data. Statistical differences among the different groups were compared using one-way analysis of variance. The least significant difference test and Dunnett-t test were used for homogeneous and heterogeneous variance, respectively. Differences were considered statistically significant at *P*< 0.05. Using Cytoscape 3.9.1, correlation network diagrams were created. GraphPad Prism 8.0.2 and the Parsonalbio Genescloud Platform were used to process the images.

## 3 Results

### 3.1 PAS840 component analysis results

The LC-MS/MS results revealed 46 categories of small molecule compounds, including 223 small molecule compounds. The top 5 categories with respect to content included carboxylic acids and their derivatives, accounting for 15.7% of all compounds, followed by benzene and its substituted derivatives (14.89%), fatty acids (7.17%), and phenols (5.83%). Detailed information on other components, including ms, exact_mass, ppm, and class data, can be found in the Supplementary Material (LC-MS/MS identification table).

### 3.2 PAS840 reduces pathologic brain tissue damage

**FIGURE 1:**
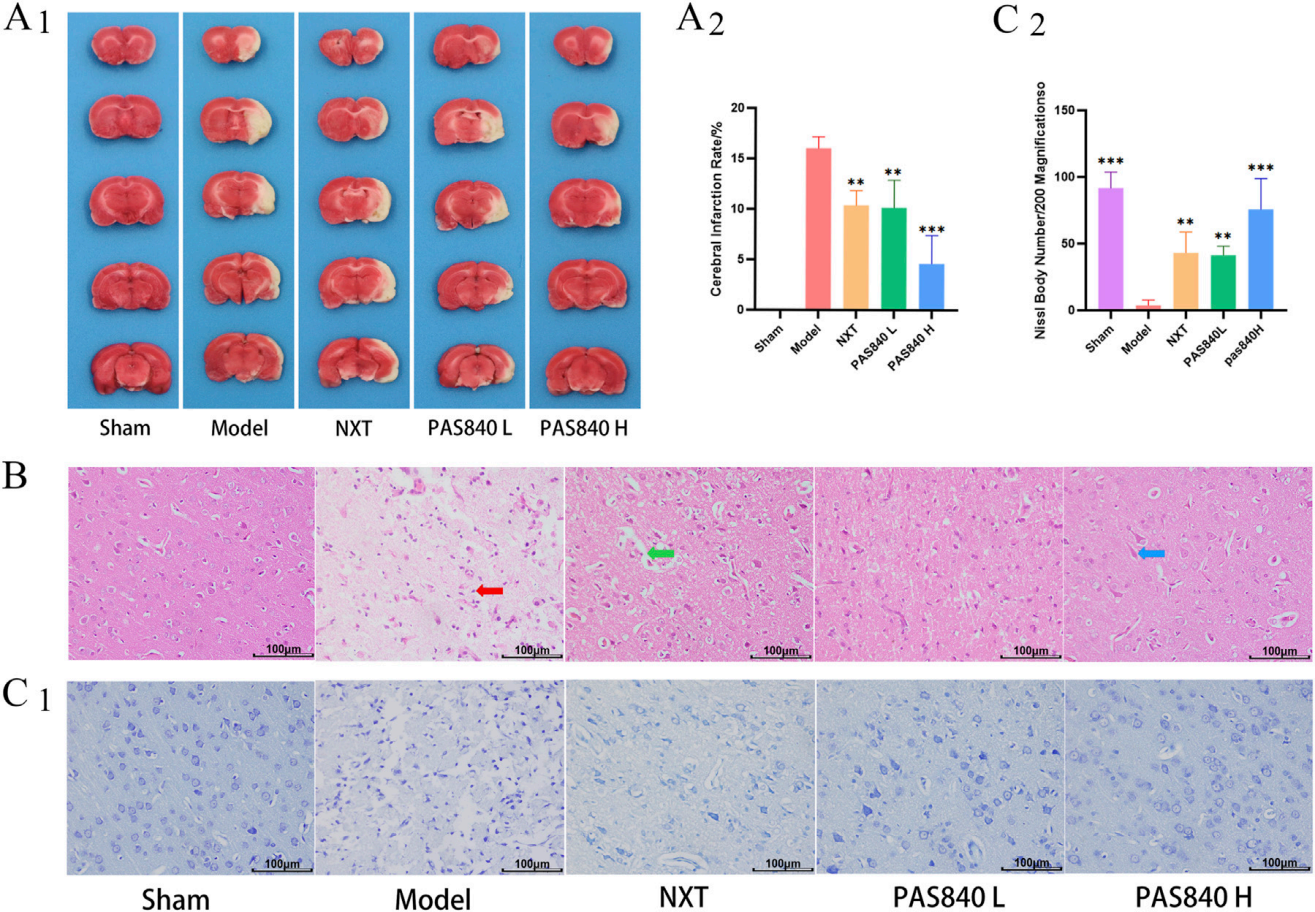
Pathologic tissue staining (n = 3). **(A1)**, TTC staining of brain tissue sections; **(A2)**, quantitative analysis of infarction rate in tMCAO rats; **(B)**, HE staining ×200; **(C1)**, Nissl body staining ×200; **(C2)**, Nissl body count. **P< 0.01, ***P< 0.001 vs. the Model group. 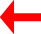
indicative of inflammatory cell infiltration; 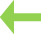
indicative of cell vacuolization; 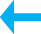
indicative of neuronal cell deep staining crumpling apoptosis.

The infarction rate of the rat brain tissue in each group was determined using TTC staining. The results indicated that the infarction rate of the three treated groups was significantly lower than that of the Model group. Moreover, the infarction area gradually decreased with an increase in the dose of PAS840 (Figure 1A1,A2). Similarly, the results of HE staining demonstrated that the administration of PAS840 and NXT effectively reduced the infarct area of the cerebral cortex tissue, inflammatory cell infiltration, and neuronal cell deep staining wrinkled apoptosis (Figure 1B). In the cortex of the brain of rats in the Model group, the number of Nissl bodies was significantly lower than that in the treated group (Figure 1C1,C2). This indicates that pathological damage to the brains of tMCAO rats was successfully attenuated by PAS840 treatment.

### 3.3 Neural function score and

**FIGURE 2:**
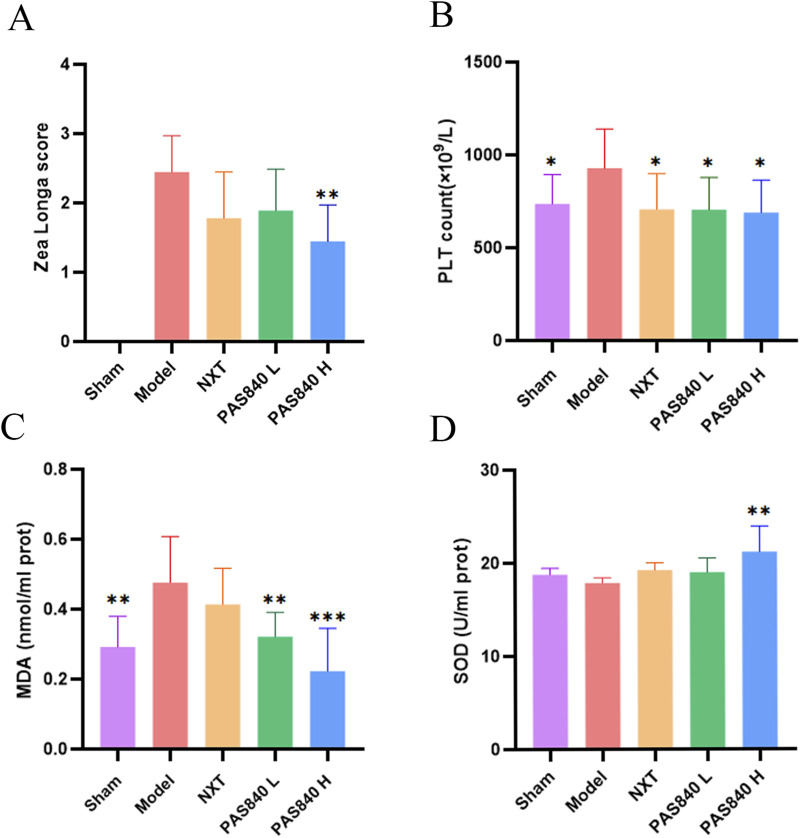
Effect of PAS840 on biochemical indices **(A)** Zea Longa score (n = 9); **(B)** PLT count (n = 9); **(C)** MDA content (n = 6); **(D)** SOD activity (n = 6). *P< 0.05, **P< 0.01, ***P< 0.00 vs. the Model group. PLT, platelets; SOD, superoxide dismutase; MDA, malondialdehyde.

Using the same modeling settings, Zea Longa scoring was performed for rats in the Model, NXT, PAS840 L, and PAS840 H groups. The findings revealed that all rats in the Model group had neurological function scores of >2, whereas some rats in each of the three groups had scores of 1. Furthermore, the number of rats with low scores increased as the dose of PAS840 increased, suggesting that PAS840 successfully reduced the behavioral impairments due to IS onset (Figure 2A).

### 3.4 PAS840 affects blood PLT, MDA, and SOD indices

According to the blood analysis results, the PLT values of the Model group were significantly higher than those of the other groups. PLT stress activation was inhibited in the PAS840 and NXT groups (*P*< 0.05; Figure 2B). Additionally, rats in the two PAS840 groups had significantly lower serum MDA levels as the PAS840 dose increased (*P*< 0.01; Figure 2C). Similarly, SOD activity was significantly higher (*P*< 0.01) in the high-dose group (Figure 2D). Therefore, administering PAS840 may prevent thrombosis and suppress platelet stress activation, while lowering IS-induced oxidative stress.

### 3.5 The effect of PAS840 on gut microbiota as determined by 16S rRNA detection

#### 3.5.1 Effect of PAS840 on gut microbiota species in IS rats

**FIGURE 3:**
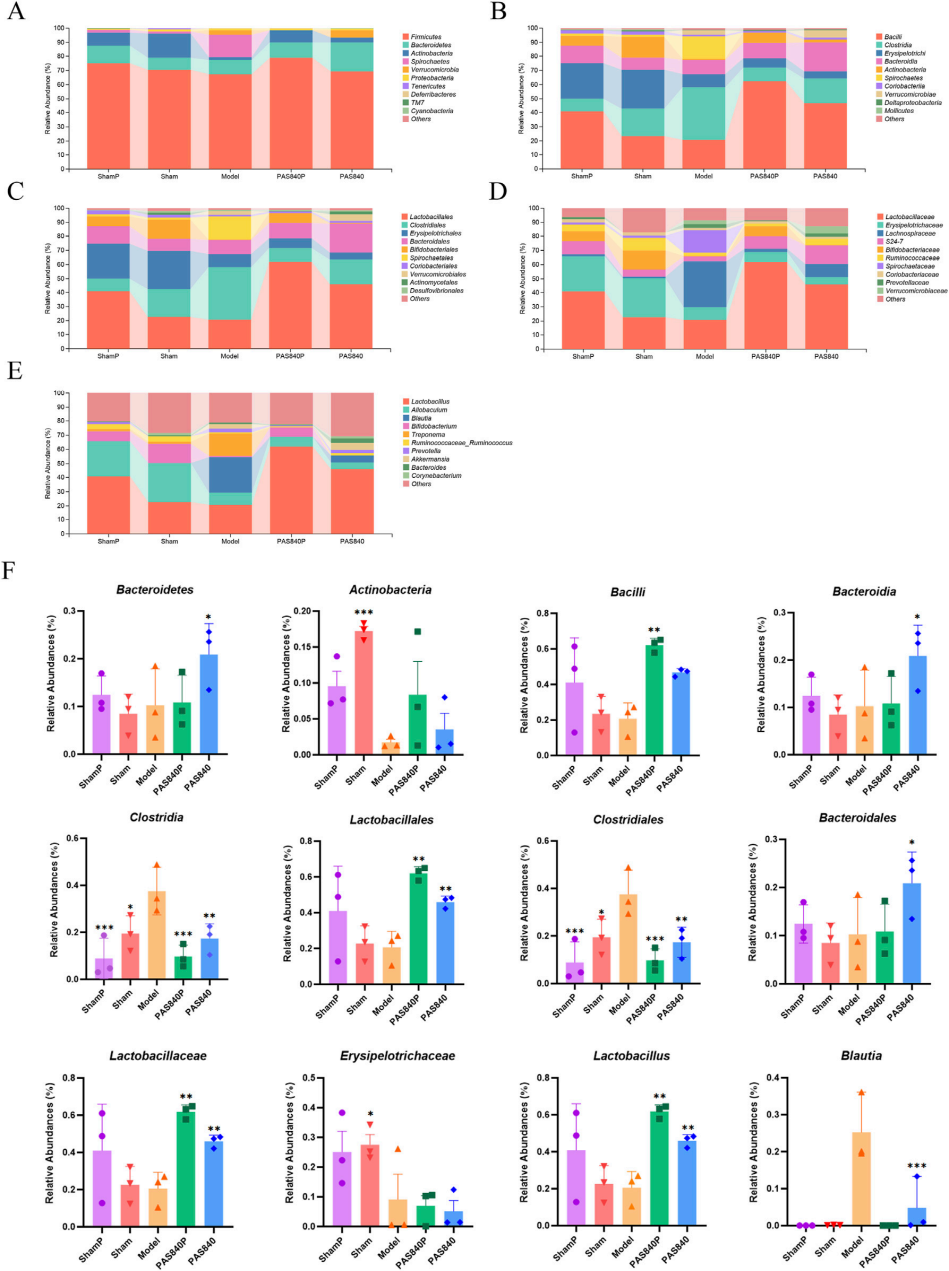
Comparison of dominant gut microbiota in IS rats after treatment with PAS840. Comparison of dominant microbiome at the phylum **(A)**, class **(B)**, order **(C)**, family **(D)**, and genus **(E)** levels; **(F)** Dominant microbiome at each classification level (n = 3). *P< 0.05, **P< 0.01, ***P< 0.001 vs. the Model group.

Rat feces from each group were subjected to 16S rRNA sequencing for microbiological investigation. The results showed that at the phylum level, *Firmicutes*, *Bacteroidetes*, *Actinobacteria*, and *Verrucomicrobia* were the dominant microbiome in the intestines in each group of rats (Figure 3A); at the class level, *Bacilli*, *Clostridia*, *Bacteroidia*, and *Actinobacteria* were the dominant microbiome (Figure 3B); at the order level, *Lactobacillaceae*, *Clostridiales*, and *Bacteroidales* were the dominant microbiome (Figure 3C); at the family level, *Lactobacillaceae* and *Lachnospiraceae* were the dominant microbiome (Figure 3D); at the genus level, *Bifidobacterium*, *Blautia*, and *Akkermansia* were the dominant microbiome (Figure 3E).

The rats’ gut microbiota homeostasis was considerably affected by both the trauma from the sham operation and IS when the ShamP and Sham groups were compared with the Model group. PAS840 treatment altered the diversity, abundance, and homeostasis of the gut microbiota in both the PAS840P and PAS840 groups. It also increased the abundance of probiotics from the *Lactobacillus* family and decreased the abundance of harmful bacteria, such as *Clostridium*, *Erysipelotrichaceae*, and *Blautia* (Figure 3F). In addition, the abundance of Blautia was very low in the ShamP, Sham, and PAS840P groups, whereas its abundance increased significantly when IS was induced. The abundance of Blautia significantly reduced after the administration of PAS840, which led to the hypothesis that Blautia is an important genus in the prevention and treatment of IS. Therefore, PAS840 may play a preventive and therapeutic role against IS by improving the abundance, structure, and diversity of gut microbiota.

#### 3.5.2 Effect of PAS840 on the abundance of species in the gut microbiota

**FIGURE 4:**
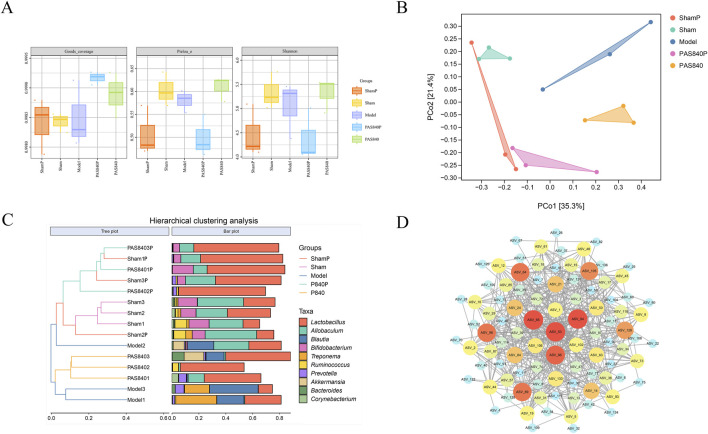
Effect of PAS840 on the abundance of gut microbiota in IS rats **(A)** goods-coverage, Pielou, and Shannon index analyses (n = 3); **(B)** Distance matrix PCoA analysis; **(C)** genus-level Hierarchical clustering analysis; **(D)** correlation network diagram. The darker colors indicate a larger circle size, while an increased number of connecting lines reflects a closer correlation between the microbiome.

The results of the analysis of the Piclou, Shannon, and Goods-coverage indices indicated that PAS840 influenced the diversity of gut microbiota in rats with IS. Additionally, the abundance of PAS840 was closer to that in the sham group, and the overall abundance uniformity of the microbiome was good (Figure 4A). Better separation across all groups and more variation in community abundance across samples were revealed by distance matrix principal co-ordinates analysis (PCoA) analysis (Figure 4B). When the findings of the hierarchical clustering analysis were integrated, it became evident that IS had a notable impact on *Lactobacillus*, *Allobaculum*, *Blautia*, and *Bifidobacterium* and that the treatment and Model groups differed in terms of abundance (Figure 4C). The findings indicate that PAS840 exerts a significant regulatory effect on the variations in microbiota abundance induced by IS.

#### 3.5.3 Relationship between gut microbiota interactions

The classification data for the predominant microbiome in each sample group was imported into Cytoscape 3.9.1 software. Subsequently, the correlation network for the predominant microbiome at each classification level was visualized (Figure 4D), and the degree value of the association network was evaluated using the Centiscape 2.2 plugin. The gut microbiota exhibited intricate linkages, as seen on the association network. The findings indicate that *Bacteroidetes* and *Firmicutes* were the two most prevalent phyla with the highest degree values. Notably, *Bacteroidetes* and *Firmicutes probiotics* are crucial to the body’s metabolic functions. According to the correlation network analysis, the dominant strains interacted with one another to provide a range of advantageous effects on the organism. Changes in bacterial microbiota also affected how different bacterial strains interacted with each other. This could affect how IS treated and prevented through the gut-brain axis.

#### 3.5.4 Predictive analysis of metabolic pathways in differential strains

**FIGURE 5:**
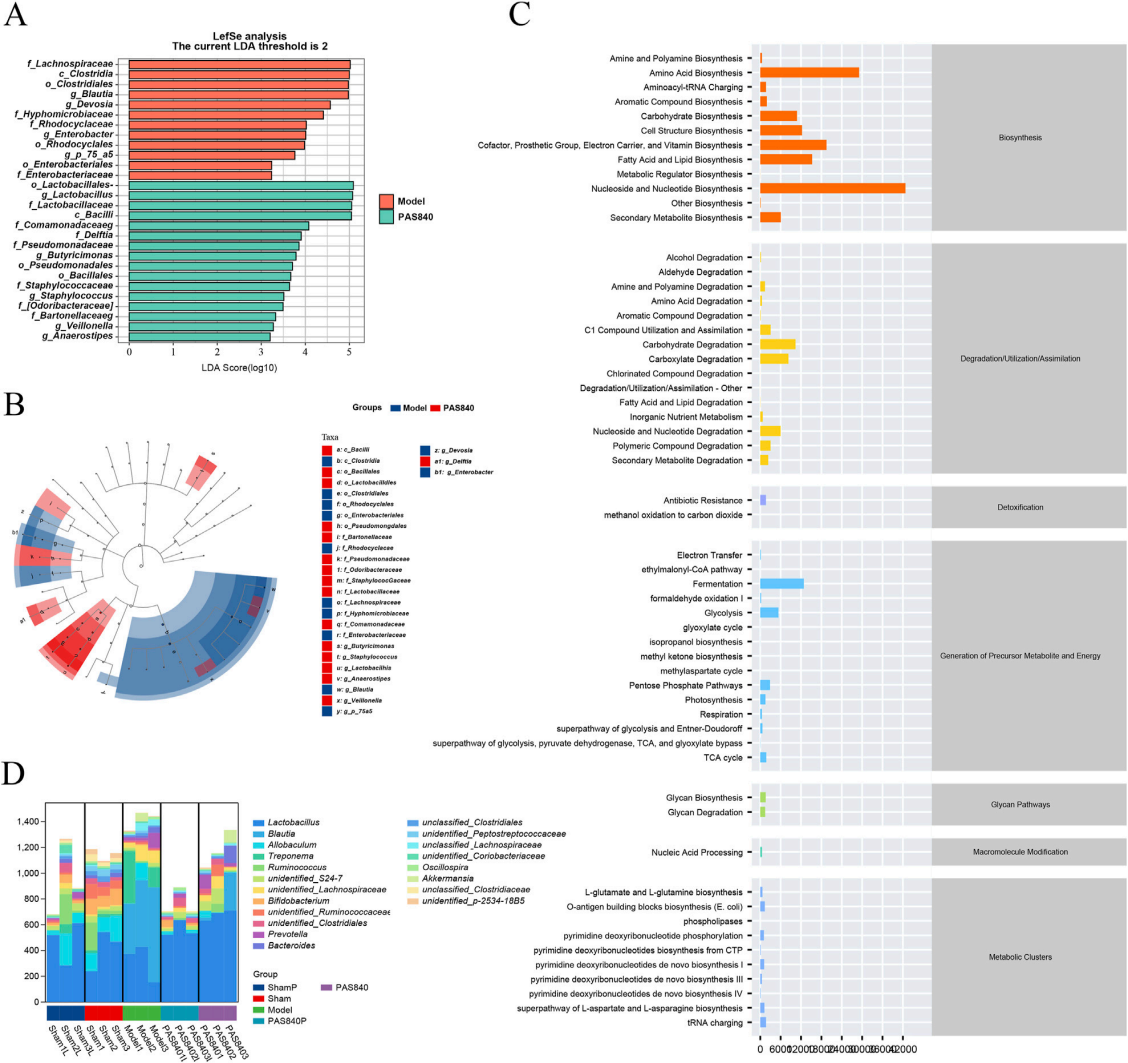
Impact of PAS840 on specie differences and metabolic pathways **(A)** LEfSe analysis of LDA histograms; **(B)** LEfSe analysis of taxonomic branching diagrams; **(C)** Metabolic pathway prediction; **(D)** genus-level metabolic pathway species composition.

LEfSe analyses (Figures 5A,B) showed more significant variation in the microbiome of rats in the Model group than in the group treated with PAS840. The highest LDA scores and dominant groups in the Model group were *Lachnospiraceae* (family), *Clostridia* (class), *Clostridiales* (order), and *Blautia* (genus). In the PAS840 group, the highest LDA scores and dominant groups were *Lactobacillales* (order), *Lactobacillus* (genus), *Lactobacillaceae* (family), and *Bacilif* (class). This suggests that the occurrence of IS had a significant impact on the abundance of these groups. The *Bacilli* family was significantly more abundant in this instance, which may be one of the possible signs of the influence of PAS840 on IS.

The KEGG database was used to perform a predictive analysis of metabolic pathway profiles (Figure 5C) and metabolic pathway species composition (Figure 5D). The results showed that the occurrence of IS significantly altered species abundance, which in turn affected many metabolic pathways, including nucleoside and nucleotide biosynthesis and degradation, amino acid biosynthesis and degradation, vitamin biosynthesis, fatty acid and lipid biosynthesis and degradation, carbohydrate biosynthesis and degradation, aldehyde and alcohol degradation, and polysaccharide degradation, amongst others.

According to the prediction of metabolic pathways, the synthesis and metabolism of nucleosides and nucleotides, amino acids, vitamins, and fatty acids are the key pathways through which PAS840 acts on IS through the gut-brain axis.

### 3.6 Untargeted metabolomics assay of PAS840 on the intestinal contents of IS rats

#### 3.6.1 Analysis of the material composition of metabolites

**FIGURE 6:**
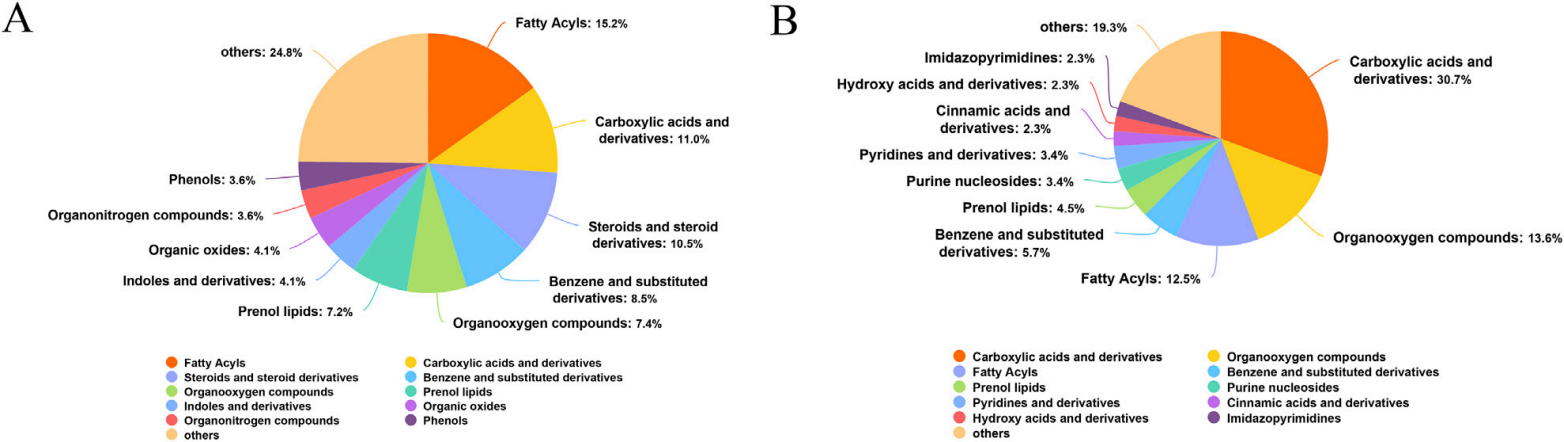
Metabolite identification analysis plots **(A)** POS model metabolite identification pie chart; **(B)** NEG model metabolite identification pie chart.

Rats in the Sham, Model, and PAS840 H groups were examined for changes in fecal metabolites. In the positive (POS) model, the intestinal microbial metabolites comprised 15.2% fatty acids, 11.0% carboxylic acids and derivatives, 10.5% steroids and steroid derivatives, 8.5% benzene and substituted derivatives, and 7.4% organooxygen compounds (Figure 6A). In the negative (NEG) model, the largest proportion was 30.7% for carboxylic acids and derivatives, followed by 13.6% and 12.5% for organooxygen compounds and fatty acids, respectively (Figure 6B).

#### 3.6.2 Evaluation of untargeted metabolomics assay models

**FIGURE 7:**
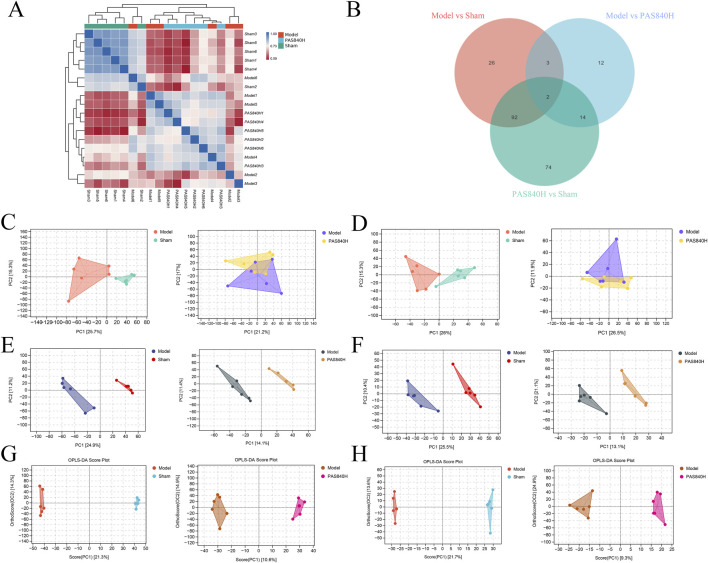
Effect of PAS840 on metabolites of gut microbiota in IS rats (n = 6) **(A)** Heatmap of metabolite correlation of samples; **(B)** Venn diagram of metabolically differentiated substances; **(C)** POS pattern of PCA scoring diagram; **(D)** NEG pattern of PCA scoring diagram; **(E)** POS pattern of PLS-DA scoring diagram; **(F)** NEG pattern of PLS-DA scoring diagram; **(G)** POS pattern of OPLS-DA scoring diagram; **(H)** OPLS-DA scoring diagram NEG mode.

Analysis of the fecal metabolites of rats from the Sham, Model, and PAS840 H groups demonstrates that the metabolites of the colonies from the three groups differed in abundance and expression (Figures 7A,B). Unsupervised analysis (PCA) and supervised analysis (PLS-DA) were used to differentiate the categories of all metabolic samples; both the PCA (Figures 7C,D) and PLS-DA score plots (Figures 7E,F) showed good separation among the three groups.

Compared with PLS-DA scores, OPLS-DA corrected the model with orthogonal transformation to filter out the information unrelated to classification, which improved the predictive ability of the model to a certain extent. The separation degree of OPLS-DA analysis was good, and the relationship between the samples was close (Figures 7G,H). The results show that the validation model was well-constructed, and the experiment was reliable.

#### 3.6.3 Differential analysis of metabolites

**FIGURE 8:**
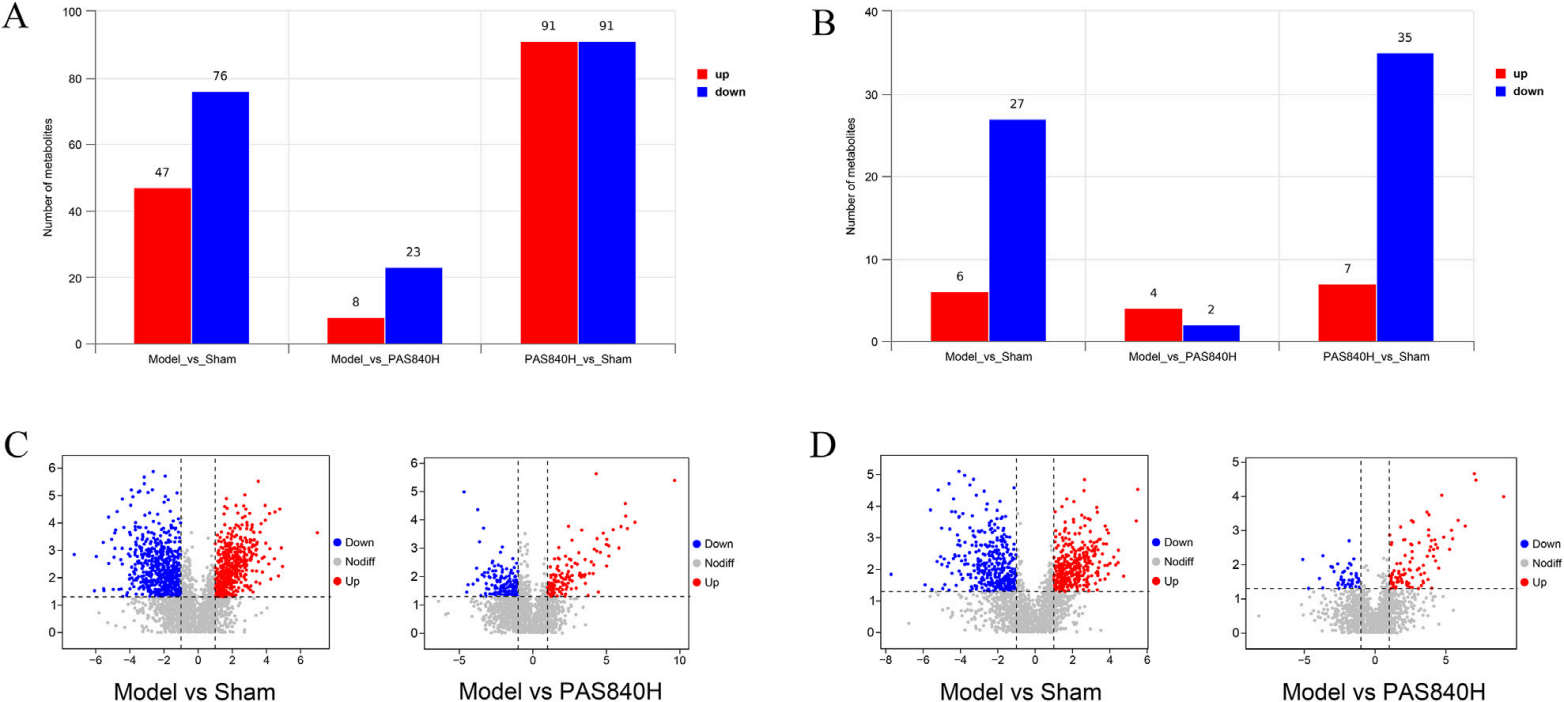
Screening visualization of differential substances **(A)** POS mode differential metabolite quantitative analysis bar graph; **(B)** NEG mode differential metabolite quantitative analysis bar graph; **(C)** POS mode differential metabolite volcano graph; **(D)** NEG mode differential metabolite volcano graph.

The metabolites in the Model group were compared and analyzed with those in the PAS840H group. Metabolites were considered differential when they simultaneously met the conditions of VIP>0, *P*< 0.05, and FC ≠ 1. Consequently, 31 differential metabolites were screened in the POS mode, of which 8 were upregulated, and 23 were downregulated. Furthermore, 6 differential metabolites were screened in the NEG mode, of which 4 were upregulated, and two were downregulated (Table 1). The bar charts and volcano plots of the sample combinations of the Model, the Sham, and the PAS840H groups for comparison of the differential metabolites are shown in the visualization display (Figure 8).

**TABLE 1:** Difference metabolites.

Id	Name	VIP	FC	*P*-value	Trend	KEGG id
M208T436	4-(2-Aminophenyl)-2,4-dioxobutanoic acid	2.8490	0.0391	0.0000	↓	C01252
M430T356	2,22-Dideoxy-3-dehydroecdysone	2.6555	0.0980	0.0002	↓	C16498
M190T361	L-2-Amino-6-oxoheptanedioate	2.8005	57.8618	0.0010	↑	C03871
M196T98	Leucodopachrome	2.5065	0.7772	0.0012	↓	C05604
M271T511	4-Hydroxyestradiol	2.5894	0.2258	0.0044	↓	C14209
M355T356	Bufadienolide	2.3493	0.2358	0.0069	↓	C16921
M132T232	(S)-4-Amino-5-oxopentanoate	2.1856	0.2439	0.0075	↓	C03741
M372T670	Cholestane	2.4496	0.7903	0.0092	↓	C19661
M331T377	Malvidin	2.1283	2.9812	0.0095	↑	C08716
M587T659	Hederagenin 3-O-arabinoside	2.2079	0.1704	0.0100	↓	C08953
M256T686	Palmitic acid	1.9791	1.1878	0.0188	↑	C00249
M228T674	Deoxyuridine	1.9915	0.7040	0.0194	↓	C00526
M299T433	(9R,10R)-Dihydroxyoctadecanoic acid	2.1060	0.4786	0.0225	↓	C08314
M172T291	Gabapentin	1.9780	0.6926	0.0240	↓	C07018
M397T542	Clionasterol	1.9438	1.8284	0.0278	↑	C19654
M265T575	Vaccenic acid	2.1003	3.3315	0.0279	↑	C08367
M224T386	Cerulenin	2.1457	0.5177	0.0282	↓	C12058
M266T245	Mirtazapine	2.0607	4.0382	0.0291	↑	C07570
M154T53	N-Acetylhistamine	2.0394	0.6110	0.0299	↓	C05135
M432T465	Vitexin	1.8244	0.3842	0.0342	↓	C01460
M107T103	Benzaldehyde	1.9749	1.9716	0.0354	↑	C00261
M311T402	2,3-Dinor-8-iso prostaglandin F1alpha	2.0156	2.7964	0.0355	↑	C14795
M348T301	4-Chloroprogesterone	1.8683	0.5134	0.0367	↓	C14676
M329T385	Corticosterone	1.7131	0.1969	0.0368	↓	C02140
M468T528	Lupeol acetate	1.7857	0.5787	0.0372	↓	C08630
M226T641	Porphobilinogen	1.9922	0.6583	0.0376	↓	C00931
M585T579_1	Bilirubin	1.8393	0.4922	0.0377	↓	C00486
M441T582_1	(24R)-24-Methylcycloarta-25-en-3-beta-ol	1.9208	0.3417	0.0404	↓	C11513
M311T240	N-Desmethylcitalopram	1.8269	0.5032	0.0459	↓	C16608
M166T660	Cyromazine	1.7565	0.8320	0.0488	↓	C14147
M312T538	Aflatoxin B1	1.8533	0.4102	0.0492	↓	C06800
M423T688	Alpha-Tocotrienol	0.6497	2.2145	0.0001	↓	C14153
M283T78	Xanthosine	5.6024	2.3171	0.0002	↑	C01762
M464T331	Glycocholic acid	0.2051	2.4087	0.0003	↓	C01921
M367T620	Tetracosanoic acid	3.7623	1.9696	0.0004	↑	C08320
M207T240	Genipin	1.7750	1.9761	0.0005	↑	C09780
M193T47	D-Glucuronic Acid	2.1656	2.0454	0.0006	↑	C00191

#### 3.6.4 Differential metabolite correlation analysis

**FIGURE 9:**
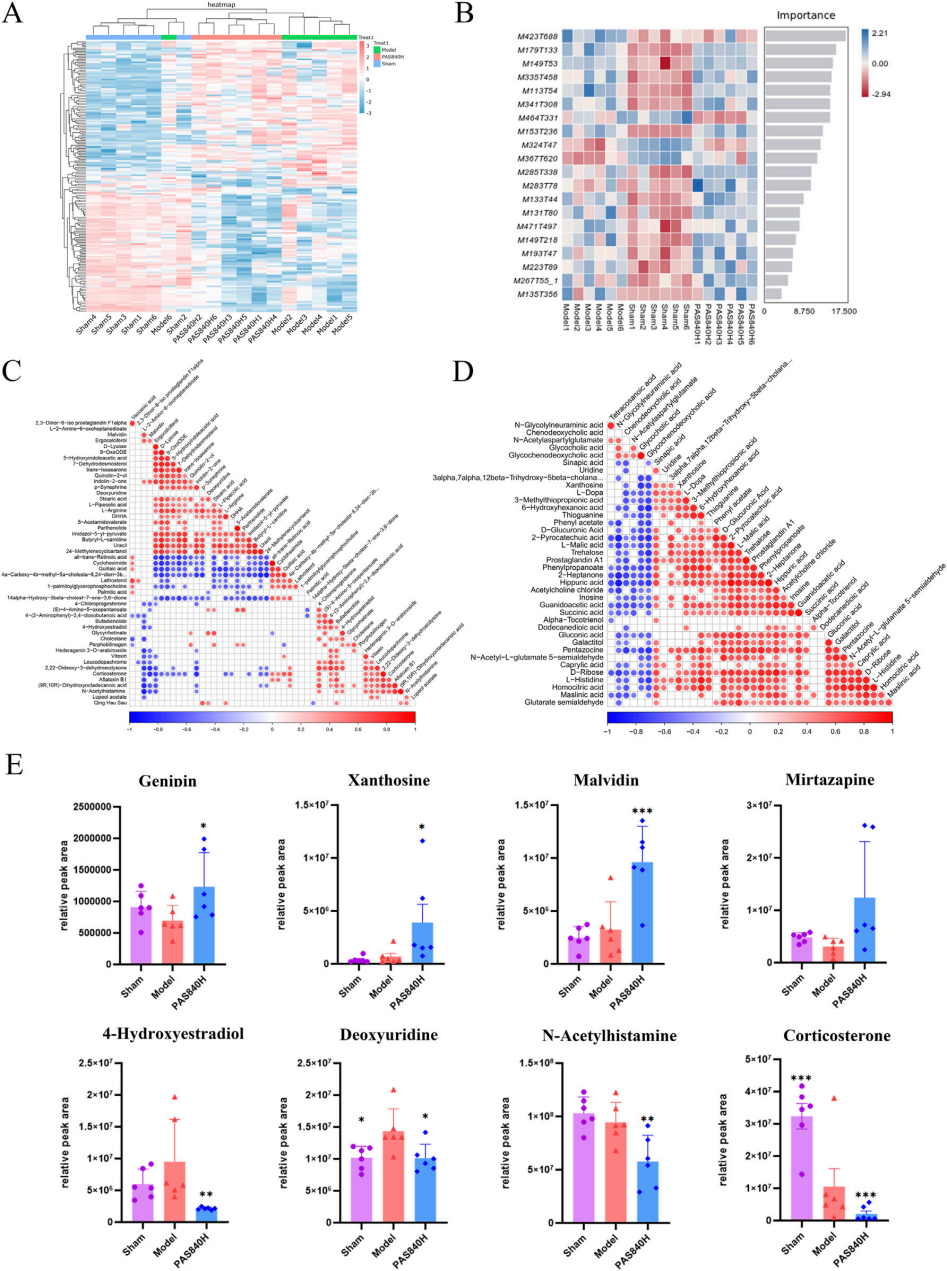
Effect of PAS840 on differential metabolites **(A)** Heat map of cluster analysis of differential metabolites; **(B)** Random Forest plot of differential metabolites; **(C)** POS model correlation analysis plot; **(D)** NEG model correlation analysis plot; **(E)** bar chart of comparative analysis of differential metabolites (n = 6). *P< 0.05, **P< 0.01, ***P< 0.001 vs. the Model group.

PAS840 administration significantly improved the metabolites of the gut microbiota altered by IS and balanced the structure of the metabolites of the microbiota. Cluster and machine learning analyses of differential substances (Figures 9A,B) showed significant differences in metabolites between groups, indicating that IS alters the metabolites of the gut microbiota.

The correlation analysis results (Figures 9C,D) demonstrated the following relationships between the differential metabolites: palmitic acid was positively correlated with Valacenic acid and negatively correlated with uracil Deoxyuridine and Parthenolide; Stearic acid was positively correlated with Ergocalciferol; Quillaic acid was positively correlated with Valacenic acid, and negatively correlated with L−Arginine and Stearic acid; Glycochenodeoxycholic acid was positively correlated with Glycocholic acid and Chenodeoxycholic acid; L−Arginine was negatively correlated with Corticosterone, Quillaic acid, and Cycloheximide; Xanthosine was positively correlated with Sinapic acid and Uridine; Malvidin was negatively correlated with 4-Hydroxyestradiol, N-Acetylhistamine, and Corticosterone. The administration of PAS840 is thought to have a significant effect on the correlation of metabolites in the colon, including fatty acids, amino acids, and nucleosides.

The metabolites of Genipin, Xanthosine, Malvidin, and Mirtazapine were upregulated, while those of 4-Hydroxyestradiol, Deoxyuridine, N-Acetylhistamine, and Corticosterone—all of which are closely related to IS—were downregulated, according to an analysis of some of the differential metabolites (Figure 9E). This implies that the administration of PAS840 may modulate gut microbiota metabolism, which influences gut microbiota metabolites, and ultimately generates compounds that may contribute to mitigating the effects of IS.

#### 3.6.5 Differential metabolite enrichment analysis

KEGG enrichment analysis was performed on differentially expressed metabolites (http://www.kegg.jp/). The number of metabolites enriched in this pathway, FDR value, and Rich factor were used to quantify the degree of enrichment based on the KEGG enrichment data.

**FIGURE 10:**
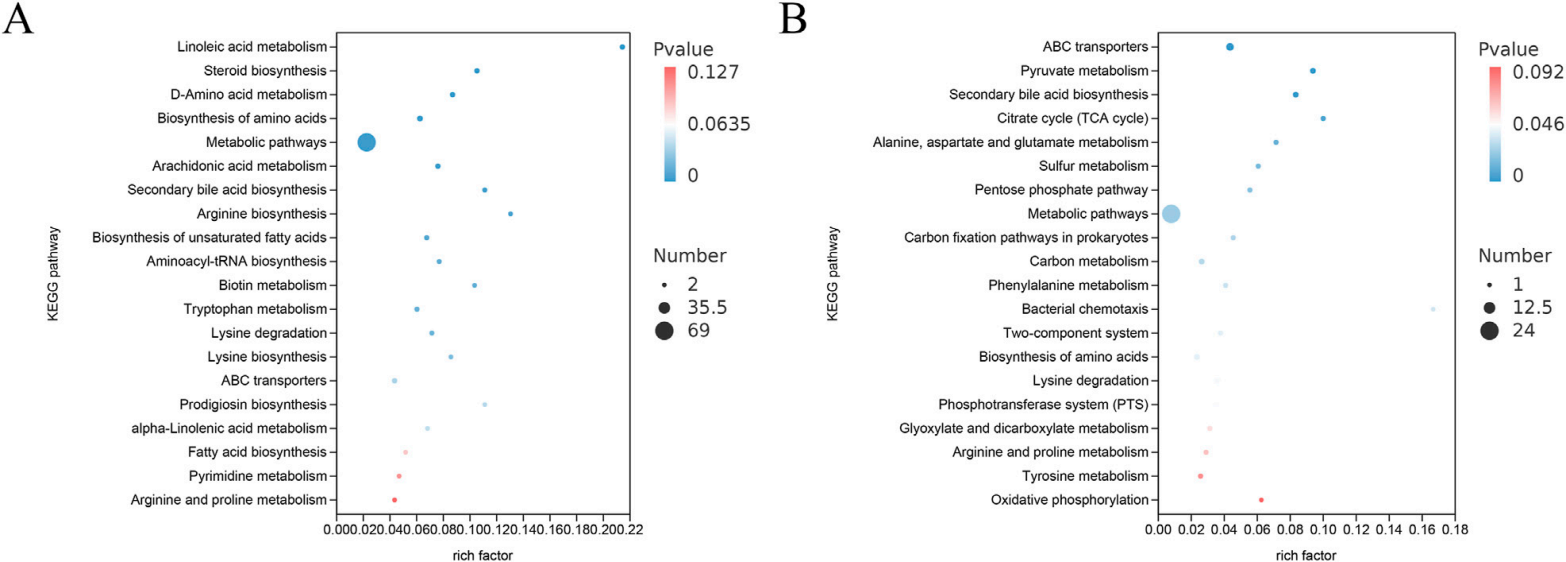
Differential substance KEGG enrichment analysis **(A)** POS model KEGG enrichment analysis; **(B)** NEG model KEGG enrichment analysis.

The top 20 KEGG pathways exhibiting the smallest FDR values, indicating the highest level of enrichment, were selected for presentation in a bubble plot (Figures 10A,B) by comparing the Sham, Model, and PAS840H groups. The plot shows that PAS840 could have a significant effect on Linoleic acid metabolism (Murru et al., 2020), biosynthesis of amino acid, arachidonic acid metabolism (Wang Z. et al., 2021), arginine and proline metabolism (Chen et al., 2020), unsaturated fatty acid biosynthesis (Venø et al., 2019), fatty acid biosynthesis, and lysine degradation (Babusikova et al., 2021), metabolic pathways highly implicated in IS disease, were positively affected.

### 3.7 Correlation analysis of gut microbiota with metabolomics

**FIGURE 11:**
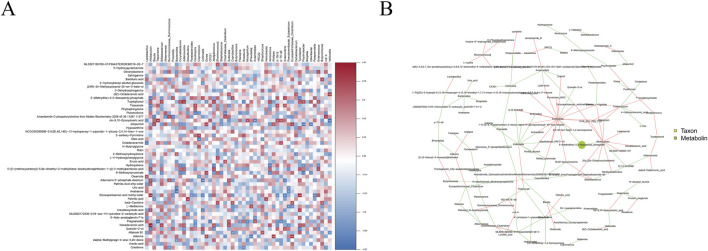
Correlation analysis **(A)** Heatmap of the association between gut microbiota and microbiota metabolites; **(B)** Association network diagram of the association between gut microbiota and microbiota metabolites. The red line between the nodes indicates a positive correlation, whereas the green line indicates a negative correlation.

Correlation heat map (Figure 11A) shows that *Lactobacillus* was positively correlated with oleamide and negatively correlated with ocadecanamide; *Blautia* was positively correlated with allopurinol and negatively correlated with palmitic acid, and *Bifidobacterium* was positively correlated with tryptophanol and nonadecanoic acid and negatively correlated with 8-methyloxykynurenate. Differential gut microbiota and metabolites were analyzed for correlations, and a correlation network diagram was constructed (Figure 11B). More connections through a node yielded more information about the associated species. Correspondingly, a node with a larger area indicates a higher abundance of the species it represents or a higher detection index.

### 3.8 Effect of PAS840 on transcriptome expression in rat brain tissue

#### 3.8.1 Differential analysis of RNA expression in IS rat brain tissue by PAS840

**FIGURE 12:**
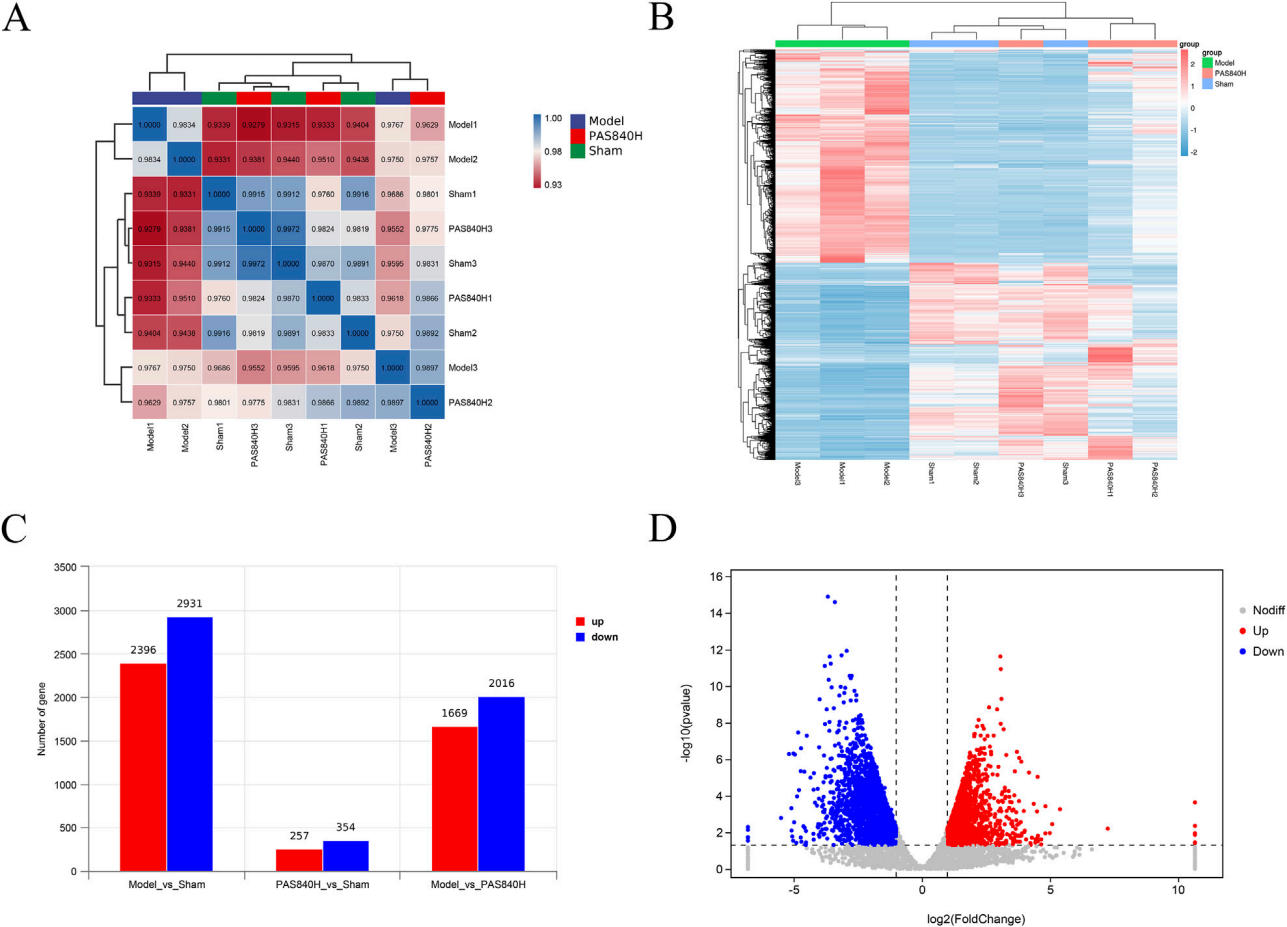
Effect of PAS840 administration on RNA expression **(A)** Heatmap of correlation analysis; **(B)** Heatmap of cluster analysis; **(C)** Differential gene expression bar graph; **(D)** Volcano plot of the DEGs between the Model vs. PAS840H groups.

Brain tissues from rats in the Sham, Model, and PAS840H groups were collected for RNA detection and differentially expressed genes (DEG) identification. The correlation analysis heatmap (Figure 12A) showed a strong intragroup correlation in each group, whereas there was a large gap between the Model and the other two groups. In the clustering heatmap (Figure 12B), there was a significant difference in gene expression among the three groups, whereas the gene expression of the PAS840H group after PAS840 administration was closer to that of the Sham than Model groups. Further differential gene analysis (Figure 12C) demonstrated that the Sham group had 5,327 differential genes compared to the Model group, of which 2,396 and 2,931 genes were upregulated and downregulated, respectively. The PAS840H group exhibited 3,685 genes that significantly differed from the Model group, with 1,699 of them upregulated and 2016 downregulated. Figure 12D depicts the distribution of volcanoes.

#### 3.8.2 Differentially-expressed gene analysis

**FIGURE 13:**
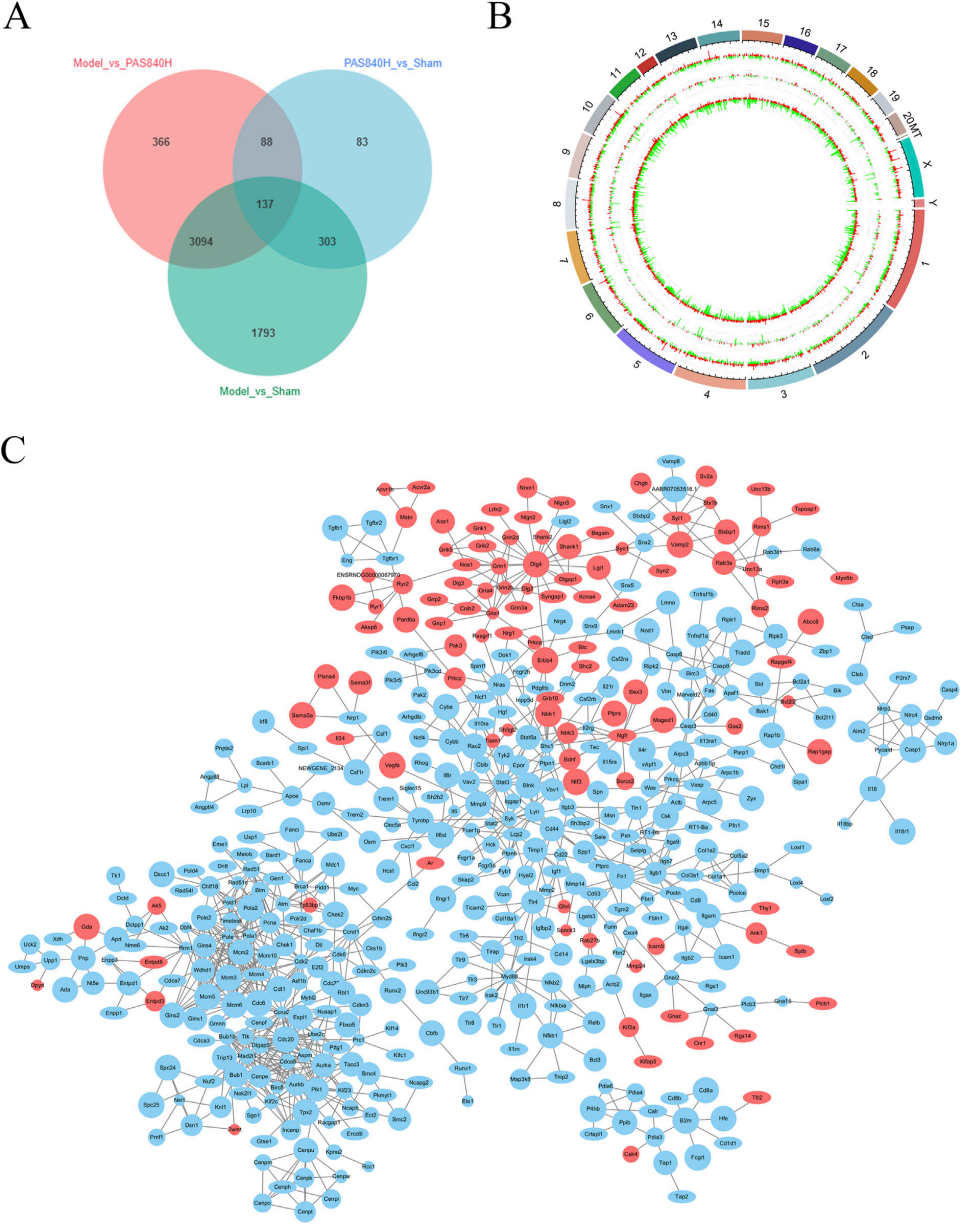
Effect of PAS840 administration on RNA expression **(A)** Differential gene expression Venn diagram; **(B)** differential gene expression genomic circle diagram; **(C)** PAS840H vs. Model group differential gene protein interaction network relationship map. The red color in the graph represents upregulated genes and the blue represents downregulated genes. A larger circle represents a larger score value of the gene.

After PAS840 administration, in the Model vs. PAS840H comparator group and the Sham vs. Model comparator group, there were 3,231 co-expressed DEGs in both groups and 137 co-expressed DEGs in the three groups, while the Model vs. PAS840H comparator group had 366 unique DEGs (Figure 13A). This may be the key gene responsible for the action of PAS840 on IS. All differential gene expression was visualized using a genome circle plot (Figure 13B).

The outermost circle of the genome circle plot represents the chromosome bands, and the results of the various differential expression analyses are shown from the outside to the inside. Red and green are the histograms of log2FoldChange values for upregulated and downregulated genes, respectively, and gray is the scatterplot of log2FoldChange values for non-DEGs. Values for genes with no differential expression are shown in gray.

DEGs in the PAS840H and Model groups were analyzed using a protein interaction network relationship diagram (Figure 13C). The results showed that in the PAS840H group, the expression of genes related to the inflammatory process, such as *Mmp9*, *Nlrp3*, *Caspase1*, *Il-1β*, *Il-6*, *Mapk13*, *Nf-кb*, *Tnfrsf*, *Cd40*, and *Cd90* was downregulated, whereas the expression of related genes that have a positive effect on IS treatment, such as nerve growth factor receptor (*Ng*fr), Nerve Growth Factor Inducible (*Vgf*), brain-derived neurotrophic factor (*Bdnf*), vascular endothelial growth factor (*Vegfa/Erbb4*) expression, was upregulated. These changes in gene expression suggest that PAS840 administration positively regulates IS, which could potentially be associated with the inhibition of the NLRP3/Caspase1/IL-1β/NF-κB signaling pathway, and the upregulation of the BDNF/VGF/NGFR pathway.

#### 3.8.3 Transcriptomics enrichment analysis

**FIGURE 14:**
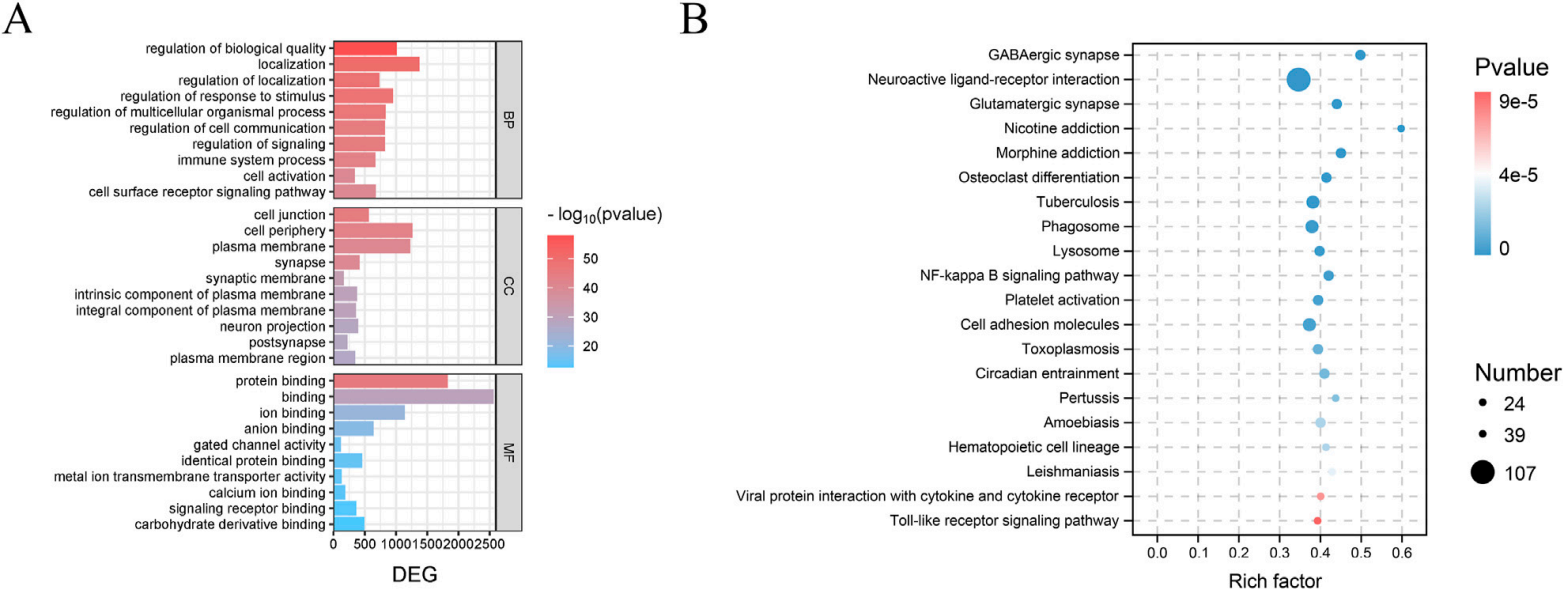
GO and KEGG enrichment analysis of IS by PAS840 **(A)** Bar graph of GO analysis; **(B)** bubble graph of KEGG analysis.

GO (Figure 14A) and KEGG (Figure 14B) enrichment analyses of the PAS840H and Model groups were compared. The GO enrichment analysis revealed that the administration of PAS840 had a positive effect on the immune system, cell activity, neuron projection, calcium ion binding, response to stimuli, regulation of cell communication, regulation of signaling, immune system processes, and other processes that had positive effects on IS rats. The KEGG enrichment analysis showed that GABAergic synaptic processes, neuroactive ligand-receptor interactions, glutamatergic synapse, toll-like receptor signaling route, NF-кB signaling pathway, and platelet activation of IS rats were all positively affected by PAS840 administration. The GO and KEGG analyses demonstrated a strong agreement with the results of the network pharmacology enrichment analysis, indicating that PAS840 could positively influence cellular activity, resistance to external stimuli, positive regulation of cytokines, control of the inflammatory response, and control of platelet activation.

## 4 Discussion

Stroke is one of the most common diseases worldwide with risk factors including acute psychological stress, smoking, atherosclerosis, hypertension, and hyperlipidemia (Prasad et al., 2020). Intense oxidative stress and inflammatory reactions ensue when IS occurs (Wang L. et al., 2022). Moreover, most stroke survivors who encounter acute ischemia experience a range of neurological impairments (Xu et al., 2021). In clinical practice, intravenous thrombolysis and endovascular interventions are frequently used as early therapies. However, both strategies entail an increased risk of bleeding, along with significant considerations pertaining to patient age and the time constraints associated with thrombolysis (Powers et al., 2018). Thus, it is imperative to enhance the physical health of patients with less neurological damage at the onset of IS, reduce the risk of complications, and enhance patients’ quality of life.

Studies have shown that PAE exhibits anti-inflammatory properties, promotes tissue repair, dilates blood vessels, lowers blood pressure, and promotes neovascularization (Zhang et al., 2023). These pharmacological effects, along with the presence of antimicrobial peptides, fatty acids, amino acids, and other components in the extract, contribute positively to the regulation of IS diseases and exert a beneficial effect on the gut microbiota (Ma et al., 2021). The gut microbiota, an important protective factor, maintains relative stability under normal physiological conditions. However, perturbations induced by external factors or stimulations can disrupt this equilibrium, potentially precipitating a spectrum of diseases.

In this study, we used TTC staining, Nissl staining, pathological section analysis, and histological examinations to examine the pathological alterations in the brain tissues of rat models with IS. The results showed that administering PAS840 could ameliorate IS-induced brain damage, lower the amount of MDA in the blood, boost the activity of SOD, attenuate platelet activity, and lower oxidative stress. These findings suggest that PAS840 can be used to prevent and treat IS.

The 16S rRNA sequencing revealed that the presence of IS can significantly alter the ecological structure of rat gut microbiota, which in turn affects the prognosis of the disease. The administration of PAS840 diminished the abundance of harmful bacteria such as *Clostridium*, *Spirochaetes*, and *Blautia*, which are directly implicated in inflammatory processes (Jiang, 2018; Rees et al., 2017). Conversely, it promotes the proliferation of beneficial microorganisms such as lactic acid-producing *Lactobacillus* and S24-7 strains within *Firmicutes* and *Bacteroidetes* phyla, which may exert probiotic effects. Reportedly, *Lactobacillus*, a probiotic, can protect patients with IS by preventing the growth of harmful bacteria through the generation of hydrogen peroxide, bacteriocins, and organic acids (Stanley et al., 2018). S24-7 reduces intestinal inflammation (Rooks et al., 2019) and effectively regulates the *in vivo* metabolism of fatty acids, amino acids, and steroids (Seo et al., 2019); it also plays a role in inhibiting β-amyloid deposition and neuroinflammation in the mouse brain (Zhu et al., 2023).

Notably, we found a low abundance of *Blautia* in the ShamP and Sham groups; however, it appeared to be significantly elevated after the onset of IS. Similarly, the abundance of *Blautia* was reduced after the administration of PAS840 compared with the Model group. *Blautia* is of interest due to its role in the alleviation of inflammatory diseases and its inhibitory effect on specific microorganisms. Moreover, it has been employed as an important indicator of inflammatory processes *in vivo* (Liu et al., 2021). Based on these results, we hypothesized that the elevation of *Blautia* in the Model group was due to a stress response caused by IS, leading to a systemic inflammatory response *in vivo*; changes in the relative abundance of *Blautia* may be a key indicator of the pathogenesis of IS. Furthermore, PAS840 may have increased probiotic abundance in the gut and attenuated the inflammatory response by regulating the microenvironment *in vivo* through various mechanisms, ultimately limiting the proliferation of *Blautia*.

Various metabolites that are strongly associated with inflammatory responses, including 4-hydroxyestradiol, deoxypyridine, N-acetylhistamine, and corticosterone, were downregulated after the administration of PAS840 (Huang et al., 2022; Xu et al., 2017). Related studies have shown that 4-Hydroxyestradiol has specific cancer-inducing effects (Lanxiang et al., 2019), and deoxyridine has a negative effect on neuronal cell value-addition and induces apoptosis in neuronal epithelial cells in rats (Rodríguez-Vázquez and Martí, 2020). In contrast, beneficial differential metabolites, such as genipin, xanthosine, malvidin, and mirtazapine, were upregulated. Studies have shown that Genipin could significantly reduce the area of cerebral infarction and attenuate neuronal damage and necrosis by inhibiting inflammatory factors such as NLRP3, TNF-α, IL-6, and IL-1β (Zhang et al., 2021c; Zhang et al., 2021b). Moreover, Xanthosine has a potent antioxidant activity, which could alleviate neuronal damage and necrosis by reducing the appearance of lipofuscin granules and neurofilaments and enhancing the expression of synaptophysin, SOD, and Bcl-xL, thus attenuating neuronal damage caused by external stimuli (Hayakawa et al., 2009). Malvidin has significant anti-inflammatory, antioxidant, and apoptosis-inhibiting effects (Bastin et al., 2020); Mirtazapine, a clinically in-use medication, is an antidepressant with specific pharmacological features, which can effectively treat depression associated with epilepsy, Alzheimer’s disease, stroke, and cardiovascular disease (Hassanein et al., 2023).

IS-induced secondary brain injury is frequently caused by brain inflammation (Ren et al., 2021). Inflammatory factors play a critical role in immune system regulation, apoptosis, value-added control, stress injury regulation, and trauma recovery (Valin et al., 2020; Yao et al., 2016). In this study, to further explore the influence of the gut microbiota and the pharmacological action of PAS840 in IS, a transcriptome analysis was performed, revealing that PAS840 exerts a potent regulatory effect on the NF-κB/NLRP3/Caspase1/Il-1β signaling pathway. *Mmp9*, *Il-6*, *Mapk*, *Cd40*, and *Cd90* genes significantly downregulated in brain tissues, indicating that the inflammatory response in the brain was attenuated by PAS840. This aligns with the results of the 16S rRNA and untargeted metabolomics analyses of the gut, demonstrating the role of probiotics in increasing beneficial flora metabolites, thereby attenuating host intestinal and systemic inflammatory responses and oxidative stress.

The metabolic pathways of the gut microbiota and the results of the differential metabolite enrichment analysis showed a simultaneous promotion of fatty acid biosynthesis, promotion of arginine anabolism, and modulation of metabolic pathways such as that of carbohydrates. Short-chain fatty acids, the fermentation products of the gut microbiome, promote neuronal regeneration and modulate recovery after stroke (Sadler et al., 2019). In addition, amino acid synthesis plays a significant role in promoting the synthesis of arginine and proline and inhibiting branched-chain amino acid synthesis. Supplemental arginine has been shown to aid in the recovery from intestinal damage and enhance the immune system (Wu et al., 2019). Arginine and proline can also stop the HIF-1α/LDHA-mediated IS inflammatory response, which in turn lowers acute inflammation and neuronal death in rats after an IS injury (Chen et al., 2020). *Bacteroides* are involved in the metabolism of arginine, proline, phenylalanine, and unsaturated fatty acids in the intestine. They play a major role in executive control and sensorimotor systems at the neural level (Zhang S. et al., 2021). These bacteria can use tryptophan and glutamate to synthesize the neuroactive compounds glutamine and GABA (Horvath et al., 2022). This is consistent with the findings of the KEGG enrichment analyses of the transcriptome. Conversely, many branched-chain amino acids make microglia-induced neuroinflammation worse by turning on the AKT/STAT3/NF-κB signaling pathway. However, *Lactobacillus* colonization has potent neuroprotective effects that lower the amount of branched-chain amino acids that build up (Shen et al., 2022).

Furthermore, the *Vegfa* and *Erbb4*, which are members of the endothelial growth factor family along with EGFR, encode core proteins involved in IS, with crucial roles in the restoration of neural function, promoting neovascularization, attenuating apoptosis of hippocampal cells, and repairing the blood–brain barrier (Pankratova et al., 2018; Xu et al., 2023). A transcriptome analysis showed that PAS840 upregulated the BDNF/VGF/NGFR pathway and increased neurotrophic nutrition to promote neurological function recovery and attenuate the risk of IS-induced depression (Pan et al., 2023). The upregulation of these genes showed a high degree of correlation with the positive regulatory effects on the gut microbiota, including the fatty acid metabolism pathway, biosynthesis of arginine and proline, and metabolism of carbohydrates.

Transcriptome enrichment analyses showed that PAS840 affected several disease pathways, including toxoplasmosis and immunological dysregulation. It positively regulates cellular resistance to stimulus injury, inflammatory responses, neural signal modulation, and anti-apoptosis. Among them, the regulation of pathways, such as neuroreceptor-ligand interactions, calcium channels, and neural signaling is relevant in various neurological diseases (Inzitari and Poggesi, 2006; Neff et al., 2021; Wang J. et al., 2022). Transcriptome enrichment analysis of the brain tissue showed that PAS840 administration positively affected the neuromodulatory GABAergic synapse, glutamatergic synapse, and neural function signaling processes. Concurrently, it controls the NF-кB and Toll-like receptor pathways to mitigate inflammatory responses, along with pathways associated with platelet activation and others that strongly correlate with IS. These effects suggest that the PAS840 administration and alterations in the structure of the gut microbiota can promote the prevention and treatment of IS from multiple perspectives.

In summary, PAS840 can effectively inhibit inflammatory gene expression, reduce platelet activity, control energy metabolism, and maintain neurological function, which can significantly attenuate IS-induced pathological brain injury in rats. Its mechanism of action may involve the regulation of the structure of the rat gut microbiota, selectively increasing the abundance of the probiotic families *Firmicutes* and *Bacteroidetes*, which have a positive effect on IS protection, and inhibiting *Spirochaetes*, *Erysipelotricaceae*, *Blautia*, and other pathogenic bacteria. In turn, it changes the structure of the metabolic products of the flora, increases the production of beneficial microbial products, and reduces the production of toxic microbial products, thereby improving the pathological state of IS. Further studies are needed to determine how PAS840 regulates the gut microbiota and its products and how they exert synergistic effects to prevent and treat IS.

## 5 Conclusion

The present study showed that the *Periplaneta americana* (L). Extract PAS840 regulates the structure of the intestinal flora in rats. It can effectively prevent and regulate the gut microbiota disorder caused by tMCAO in rats, improve the structure of the metabolites in the flora, reduce oxidative stress, and inhibit inflammatory gene expression through the gut-brain axis, thus playing a preventive and therapeutic role in IS. It is therefore a candidate for further in-depth studies aimed at the development of IS therapeutic agents.

## Data Availability

The datasets presented in this study can be found in online repositories. The names of the repository/repositories and accession number(s) can be found in the article/Supplementary Material.
