# Supplementary figures and images for: Investigation of the effects of Periplaneta americana (L.) extract on ischemic stroke based on combined multi-omics of gut microbiota
Source: Front Pharmacol. 2024 Nov 28;15:1429960. doi: 10.3389/fphar.2024.1429960 (PMC11638836; doi:10.3389/fphar.2024.1429960)

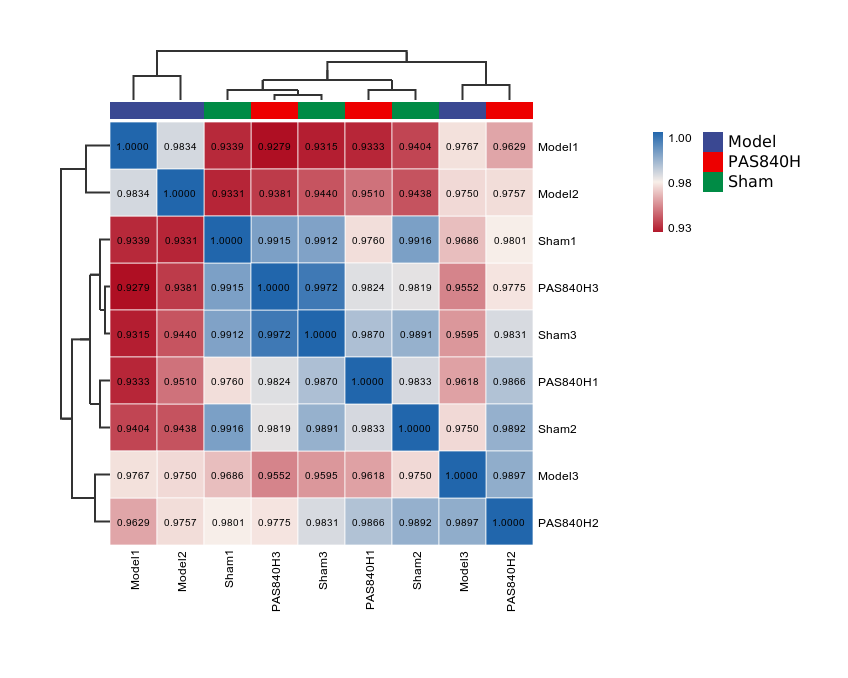

Supplement: Supplementary file 2 [file DataSheet1.zip › Correlation analysis/correlation.png]

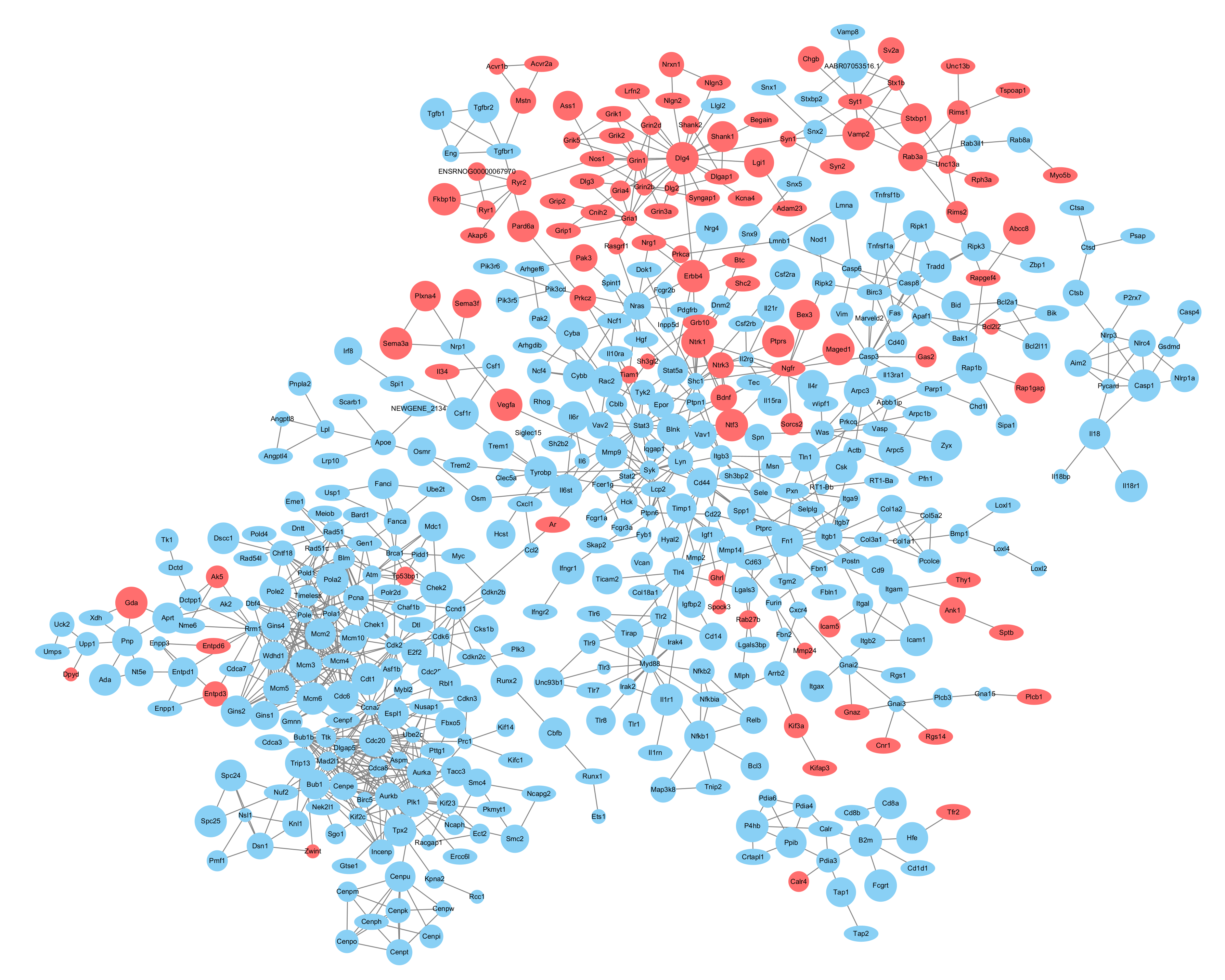

Supplement: Supplementary file 2 [file DataSheet1.zip › Correlation analysis/Interaction network diagram.png]

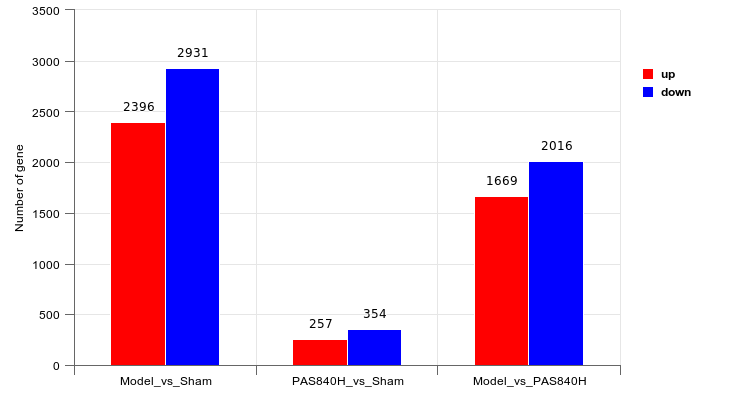

Supplement: Supplementary file 2 [file DataSheet1.zip › Differentially expressed gene/Differential expression.png]

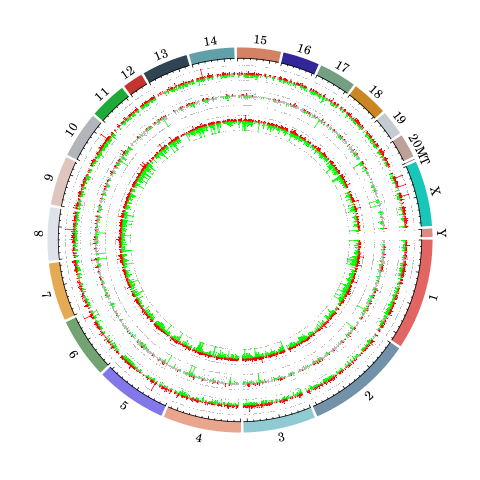

Supplement: Supplementary file 2 [file DataSheet1.zip › Differentially expressed gene/genomeCircos.png]

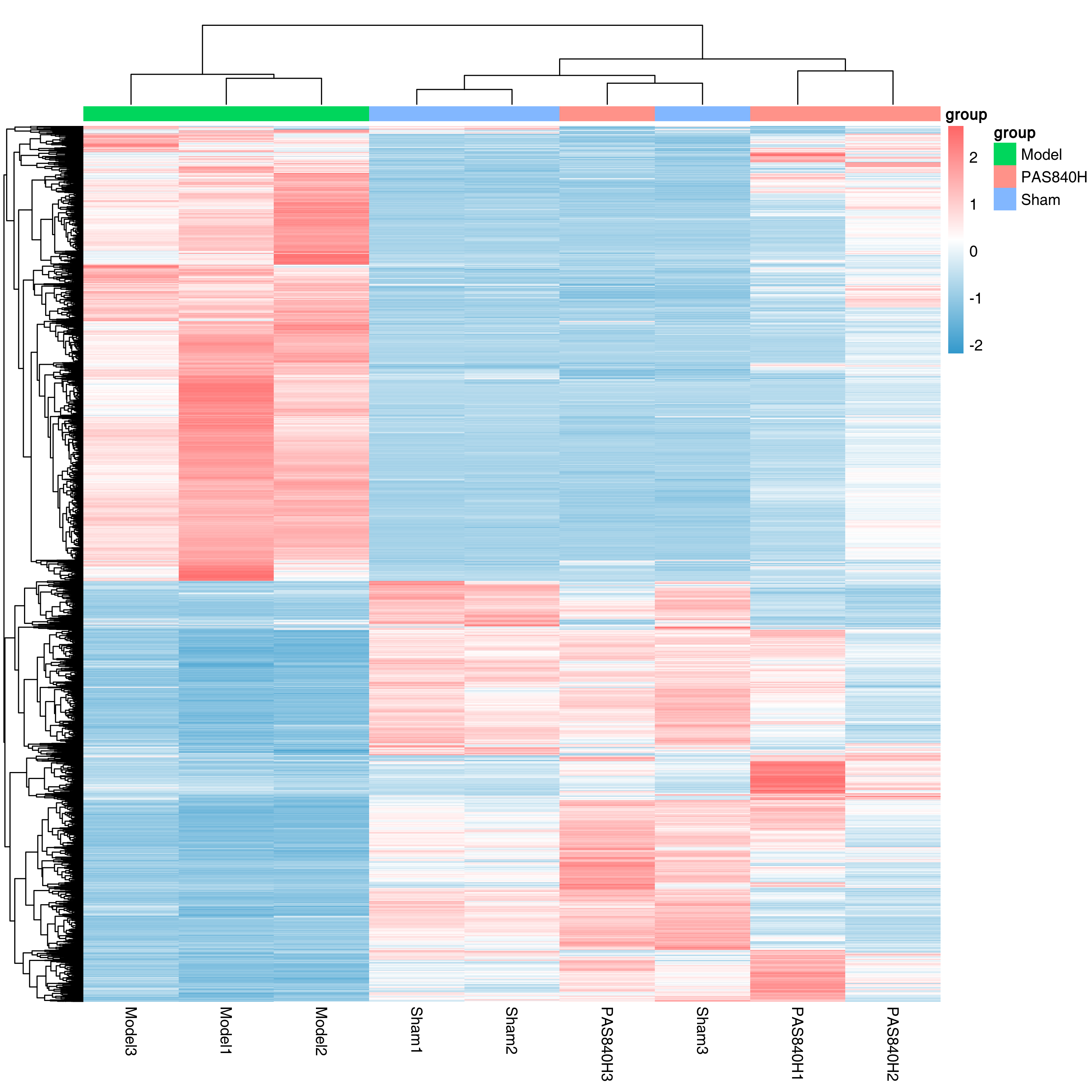

Supplement: Supplementary file 2 [file DataSheet1.zip › Differentially expressed gene/heatmap.png]

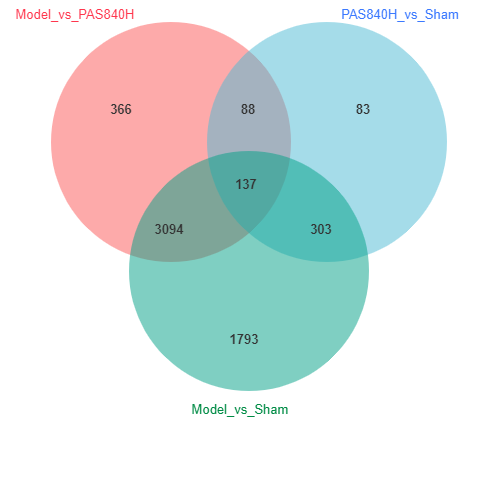

Supplement: Supplementary file 2 [file DataSheet1.zip › Differentially expressed gene/venn (1).png]

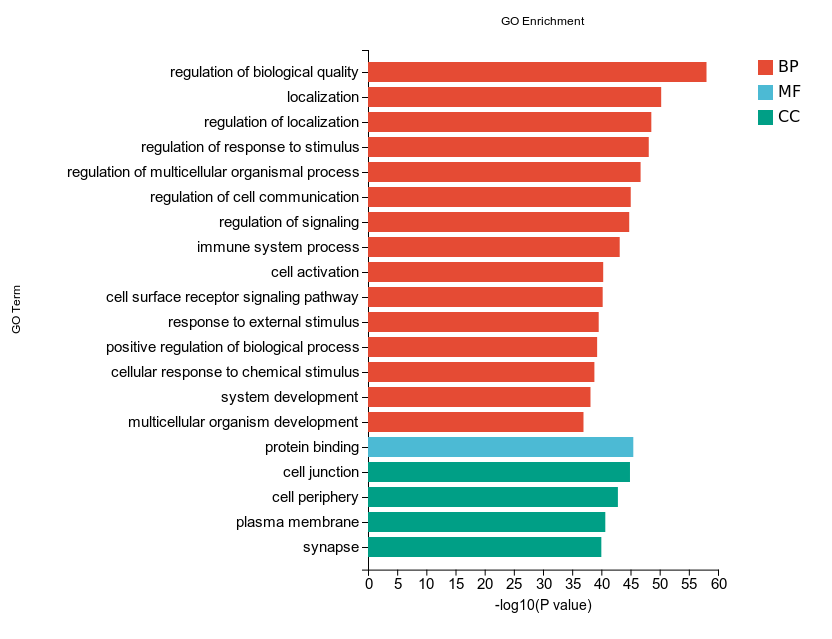

Supplement: Supplementary file 2 [file DataSheet1.zip › Enrichment analysis/GO Model_vs_PAS840H-柱状图.png]

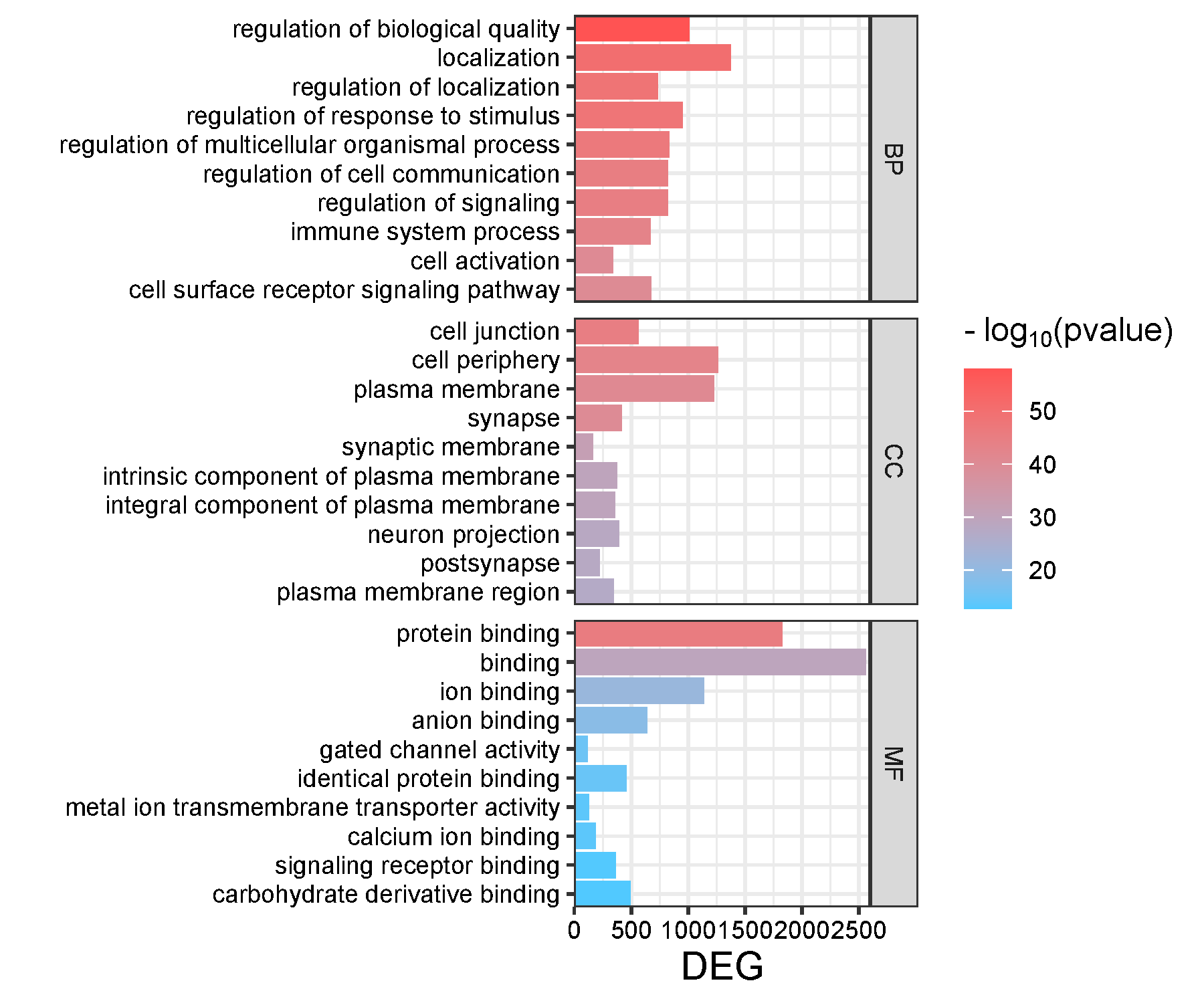

Supplement: Supplementary file 2 [file DataSheet1.zip › Enrichment analysis/GO Model_vs_PAS840H.png]

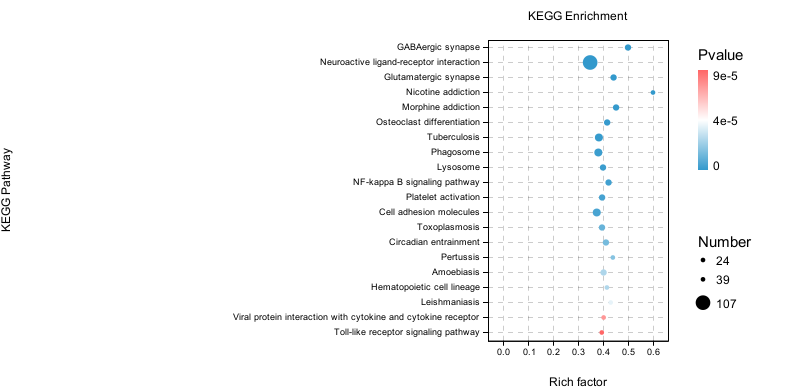

Supplement: Supplementary file 2 [file DataSheet1.zip › Enrichment analysis/Model_vs_PAS840H-因子图.png]

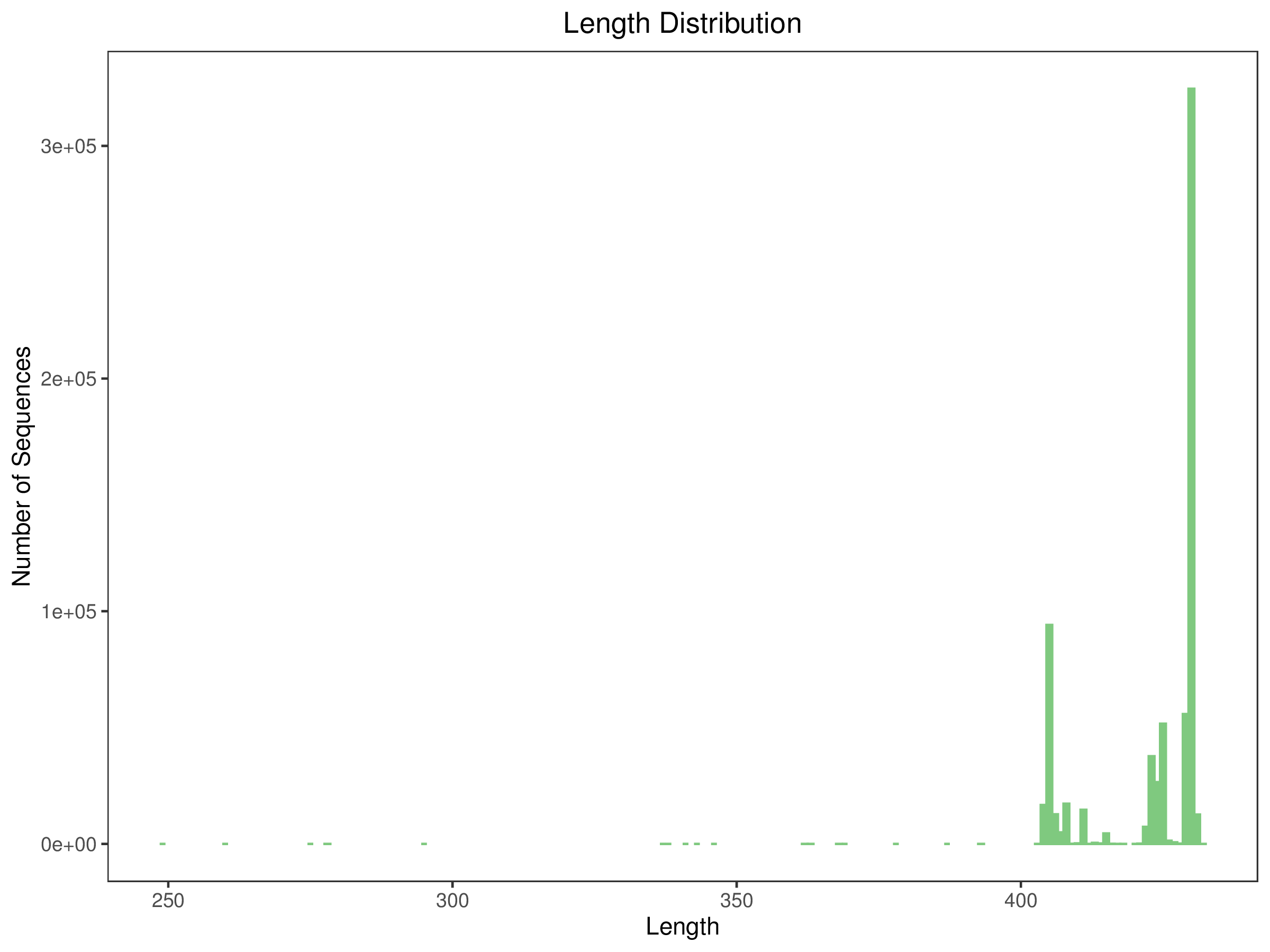

Supplement: Supplementary file 3 [file DataSheet2.zip › 2.1.2_sequences/Length_Distribution.png]

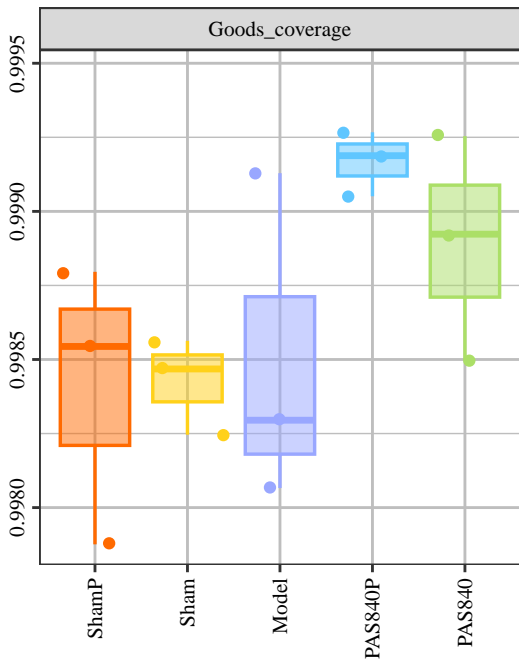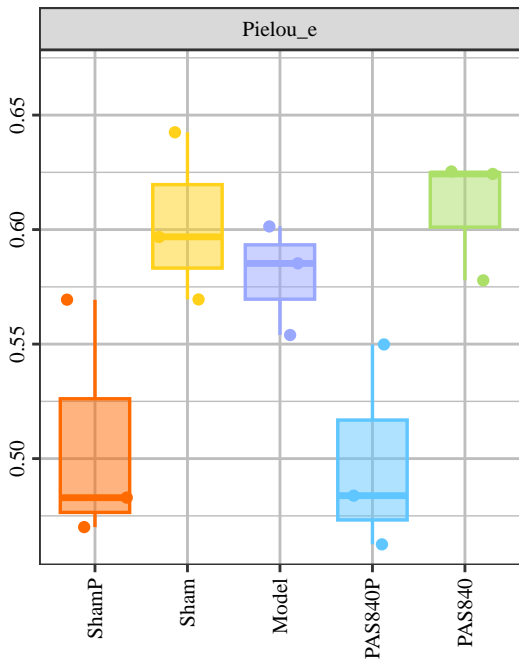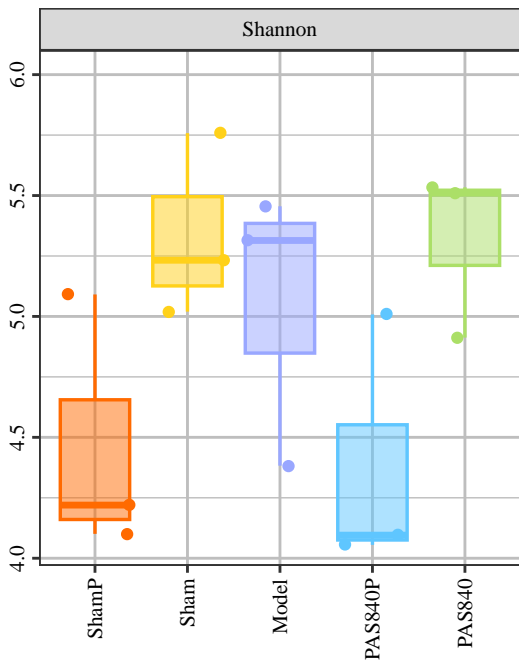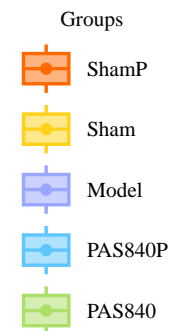

Supplement: Supplementary file 3 [file DataSheet2.zip › alpha/alpha_boxplot.pdf]

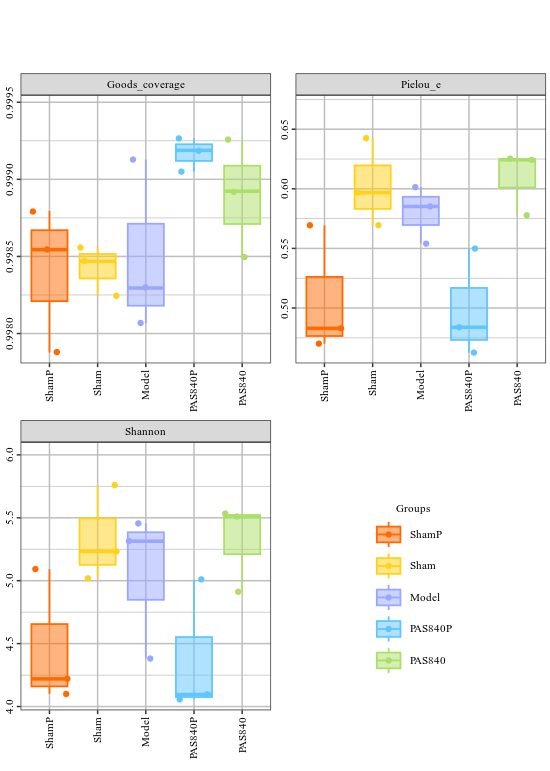

Supplement: Supplementary file 3 [file DataSheet2.zip › alpha/alpha_boxplot.png]

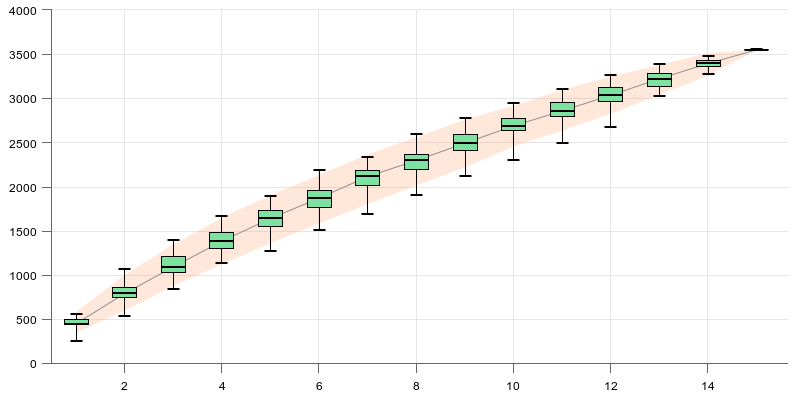

Supplement: Supplementary file 3 [file DataSheet2.zip › alpha/Specaccum.png]

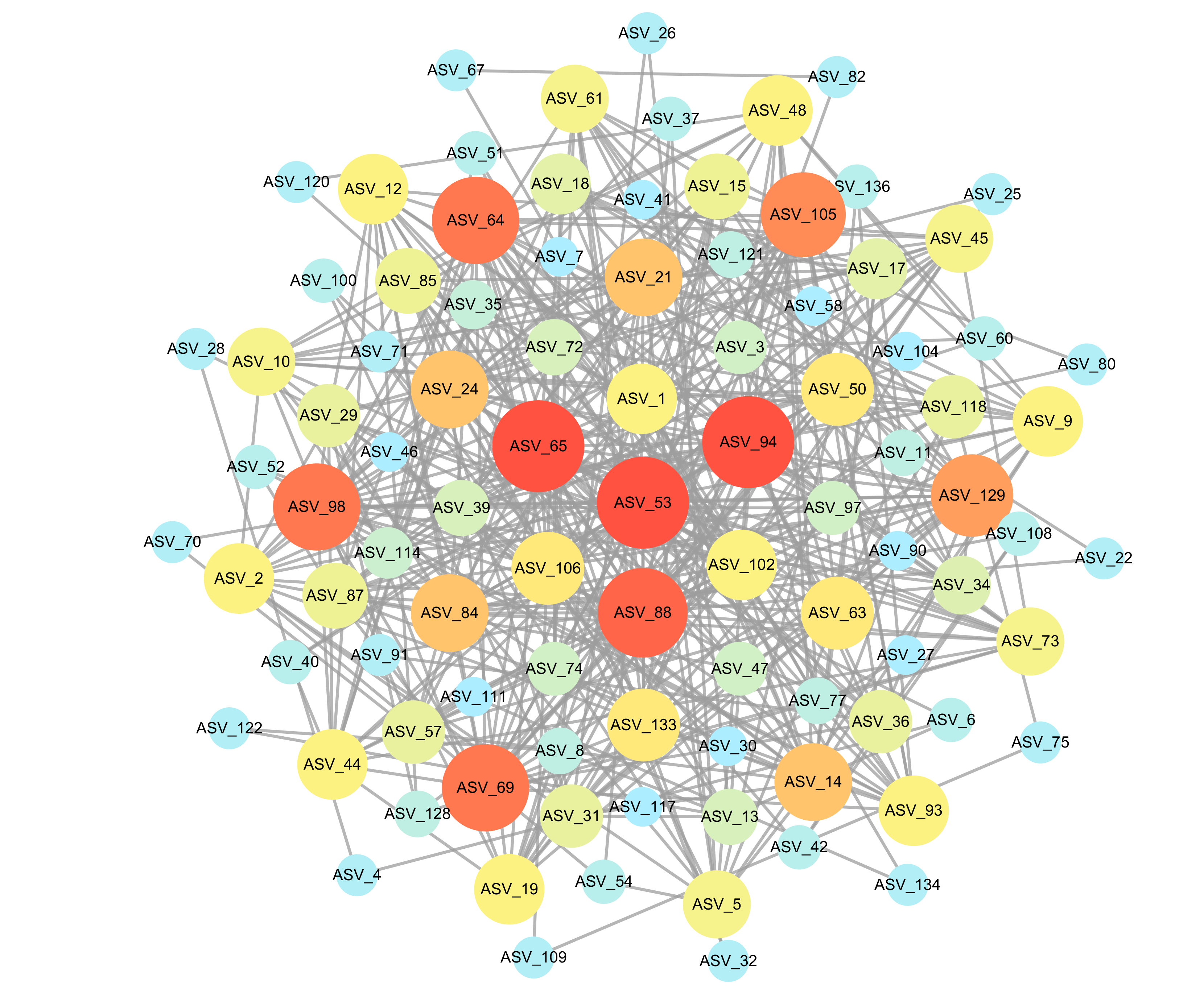

Supplement: Supplementary file 3 [file DataSheet2.zip › Association network/network.png]

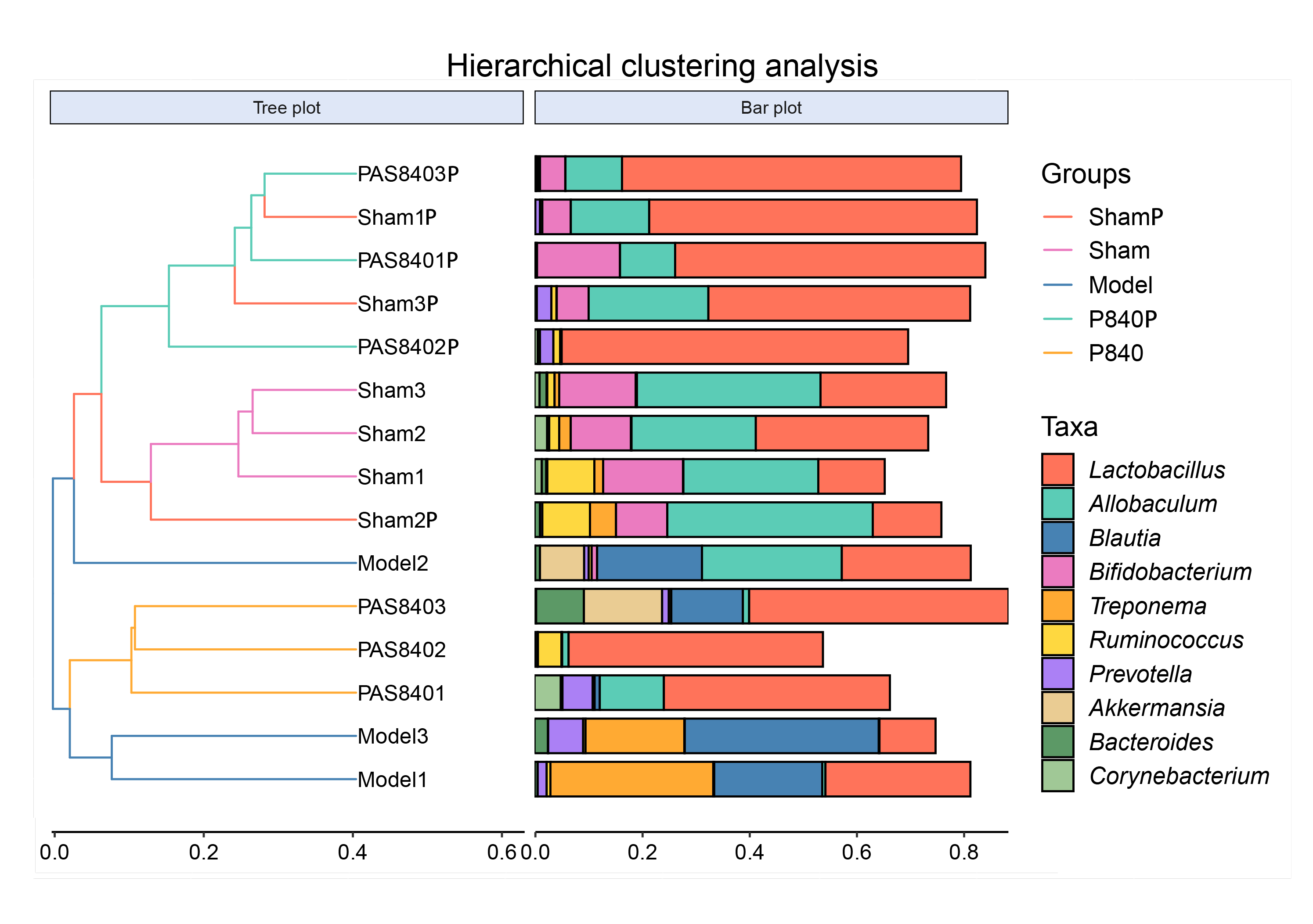

Supplement: Supplementary file 3 [file DataSheet2.zip › beta/hclust_tre.png]

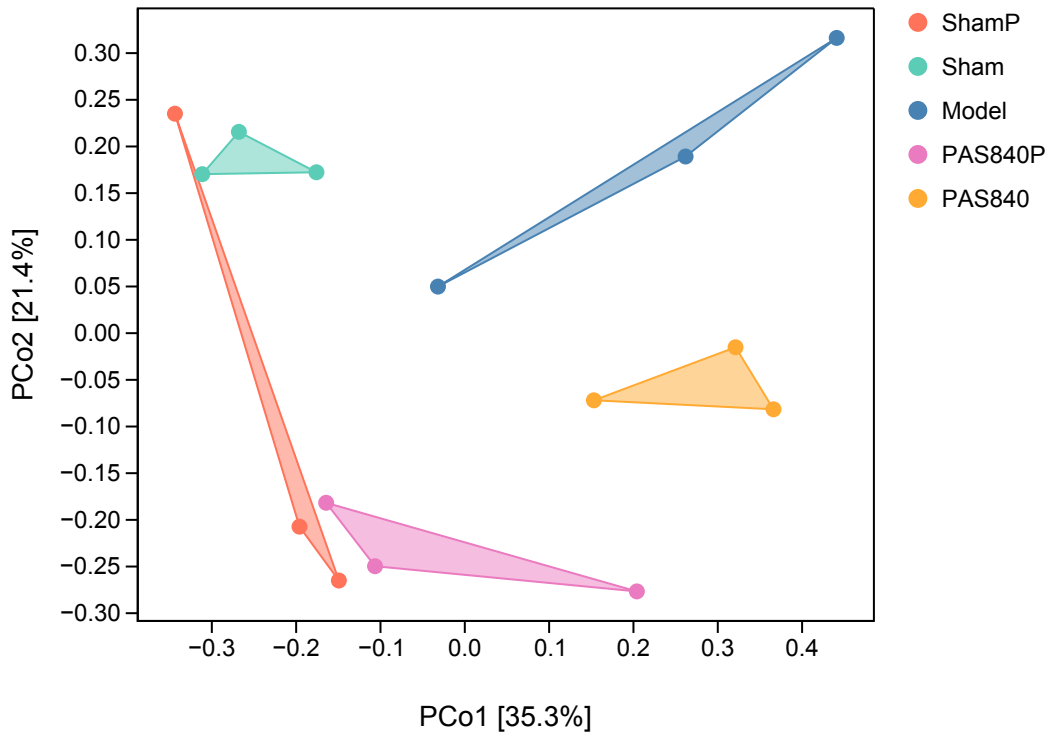

Supplement: Supplementary file 3 [file DataSheet2.zip › beta/PCoAAnalysis.pdf]

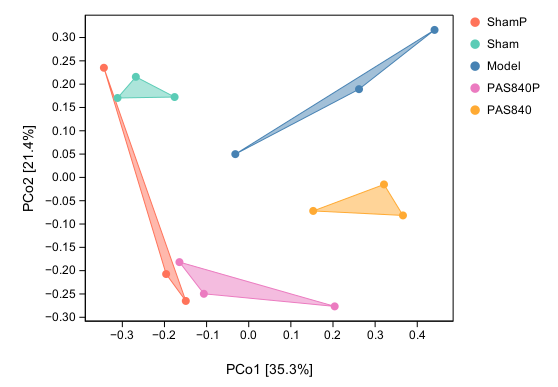

Supplement: Supplementary file 3 [file DataSheet2.zip › beta/PCoAAnalysis.png]

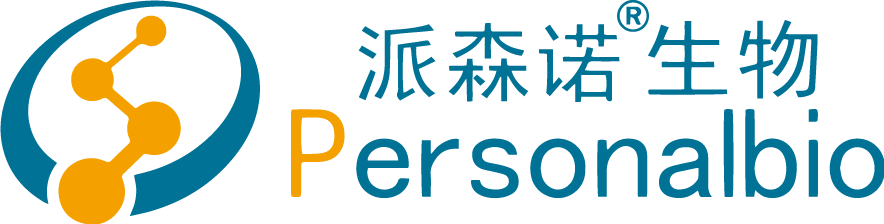

Supplement: Supplementary file 3 [file DataSheet2.zip › images/cor-logo.png]

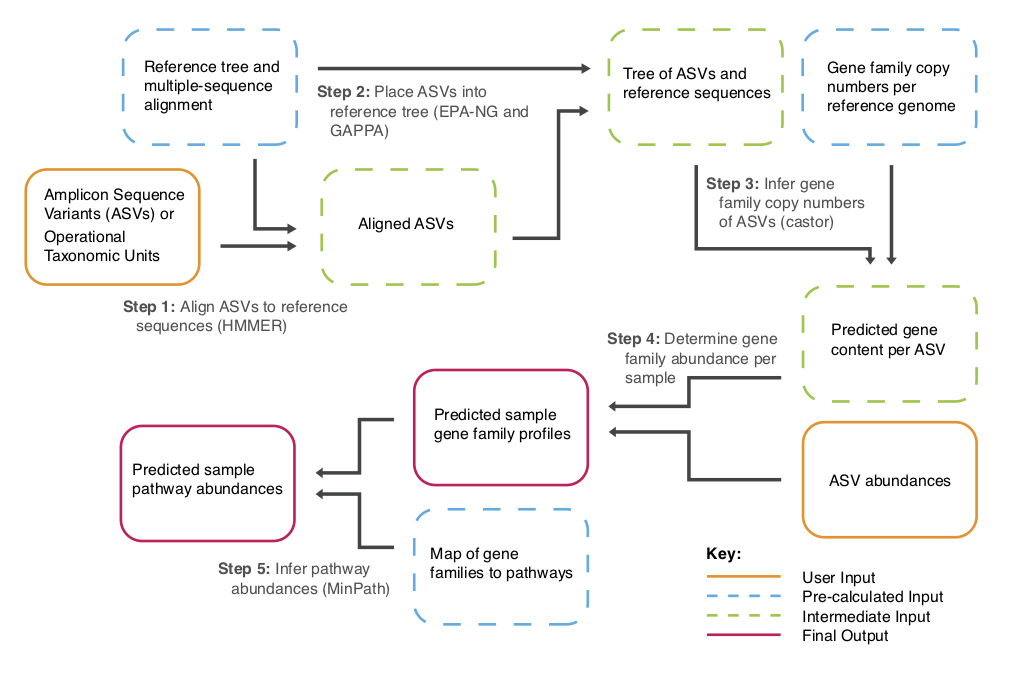

Supplement: Supplementary file 3 [file DataSheet2.zip › images/fig43.png]

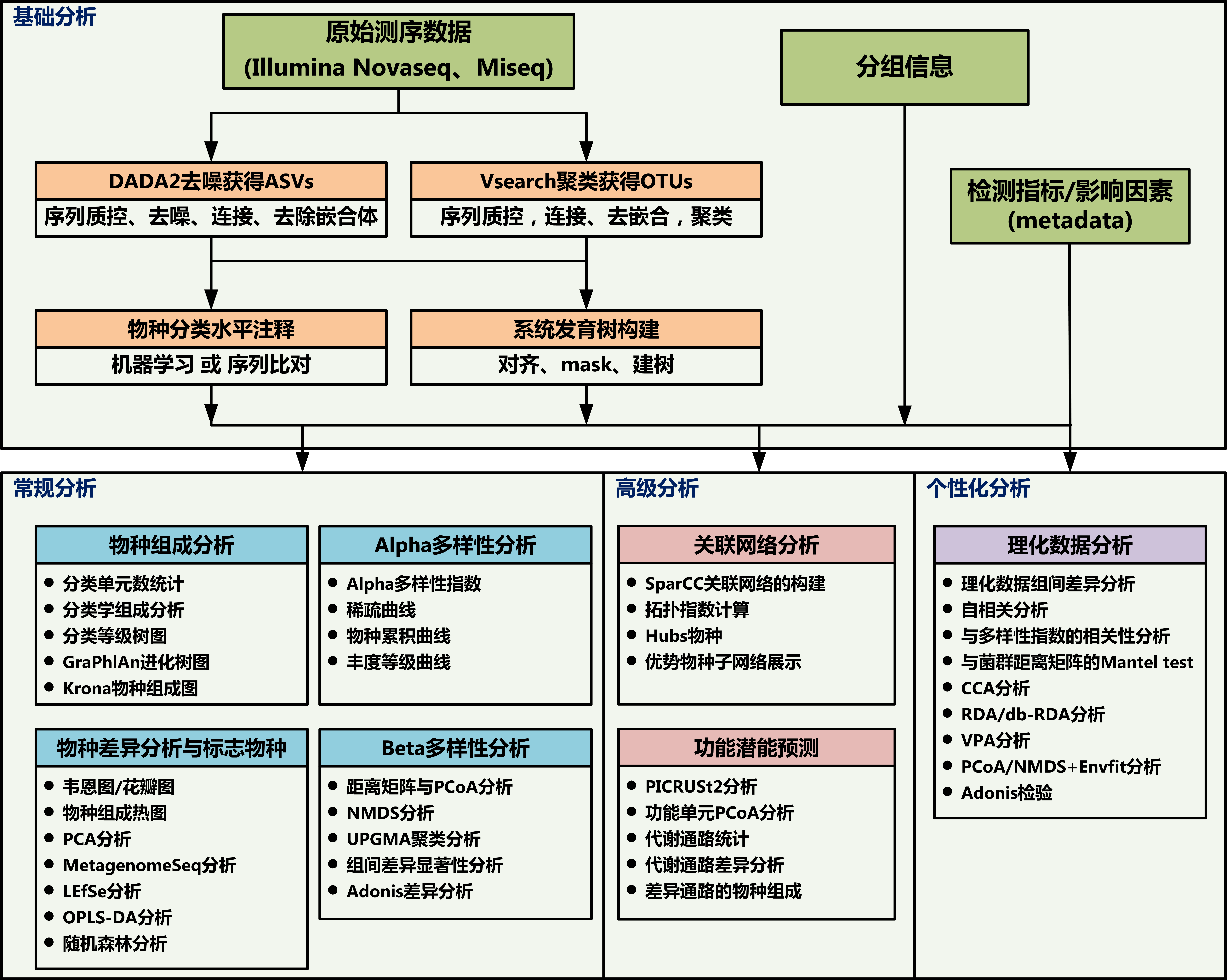

Supplement: Supplementary file 3 [file DataSheet2.zip › images/workflow.png]

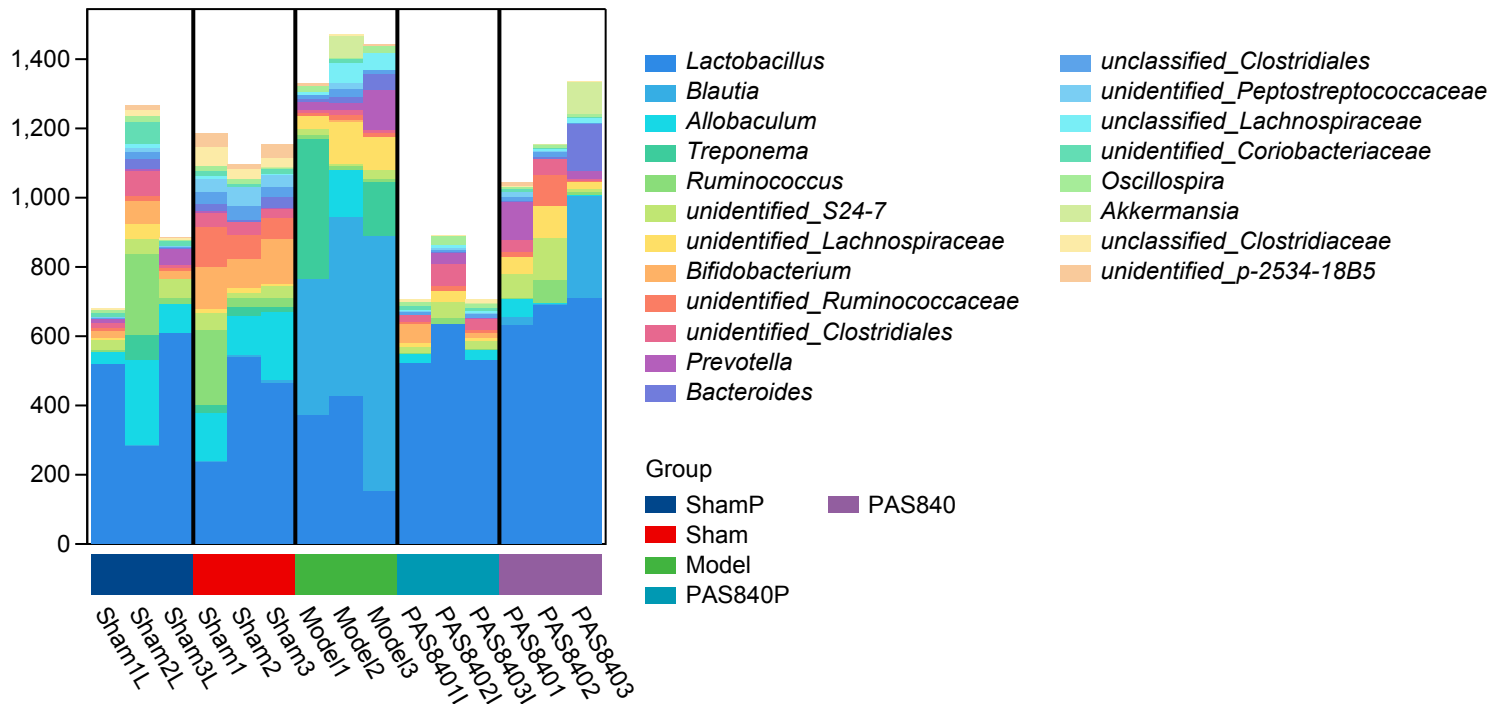

Supplement: Supplementary file 3 [file DataSheet2.zip › Metabolic pathway prediction/DifferentialPathway.pdf]

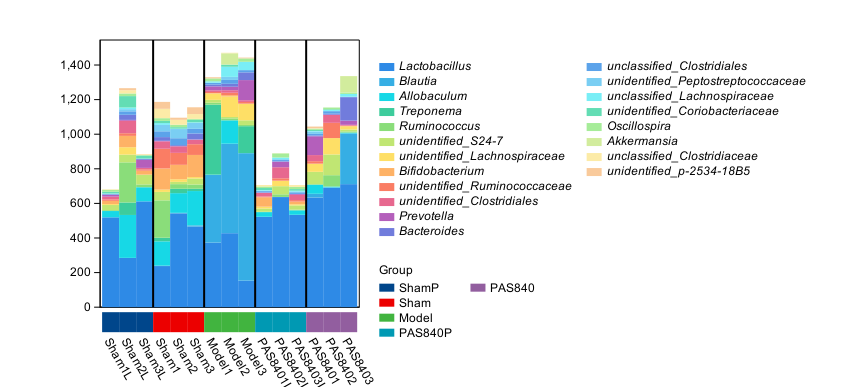

Supplement: Supplementary file 3 [file DataSheet2.zip › Metabolic pathway prediction/DifferentialPathway.png]

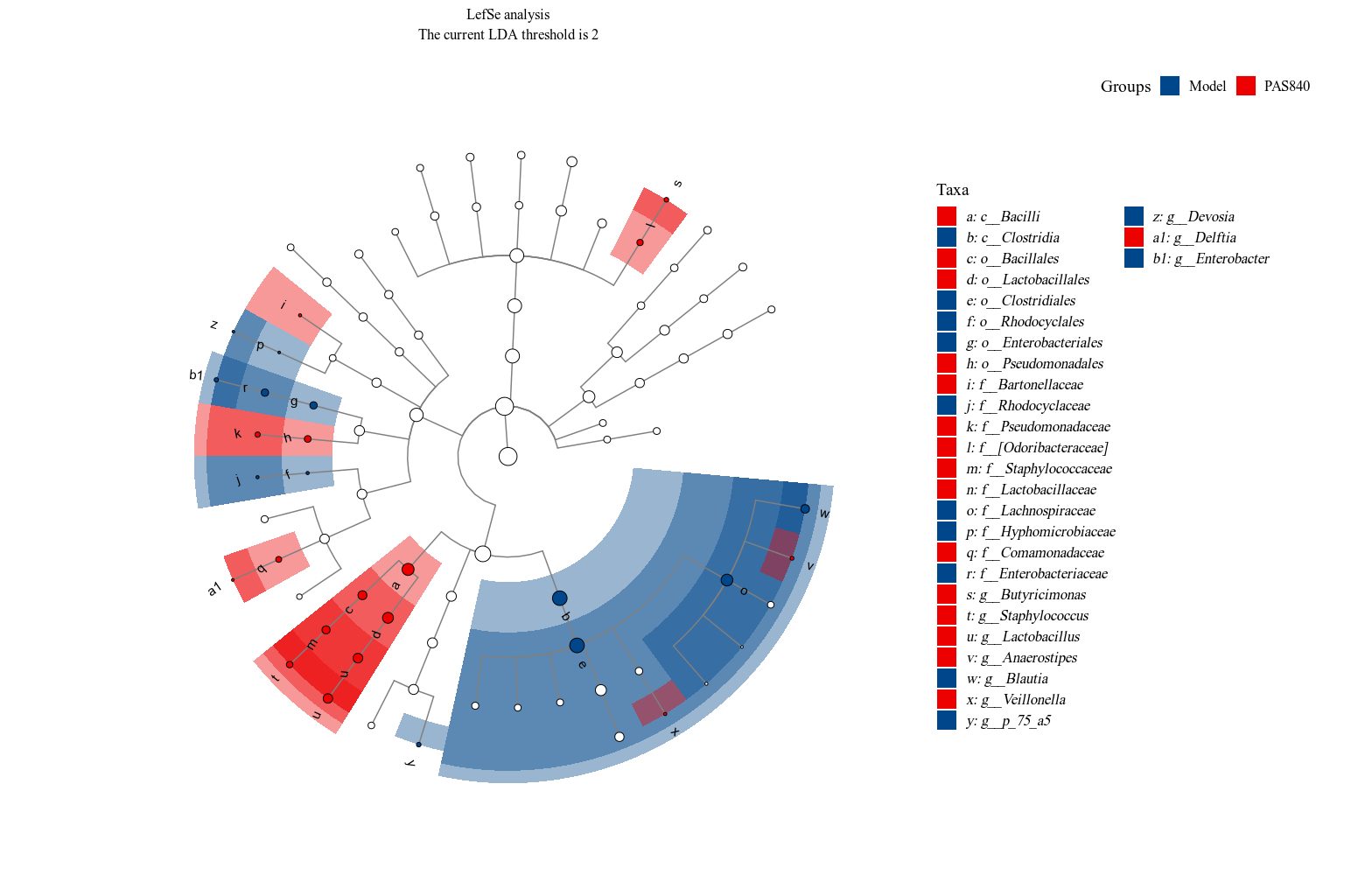

Supplement: Supplementary file 3 [file DataSheet2.zip › Metabolic pathway prediction/lefse_cladogram.png]

# LefSe analysis

The current LDA threshold is 2

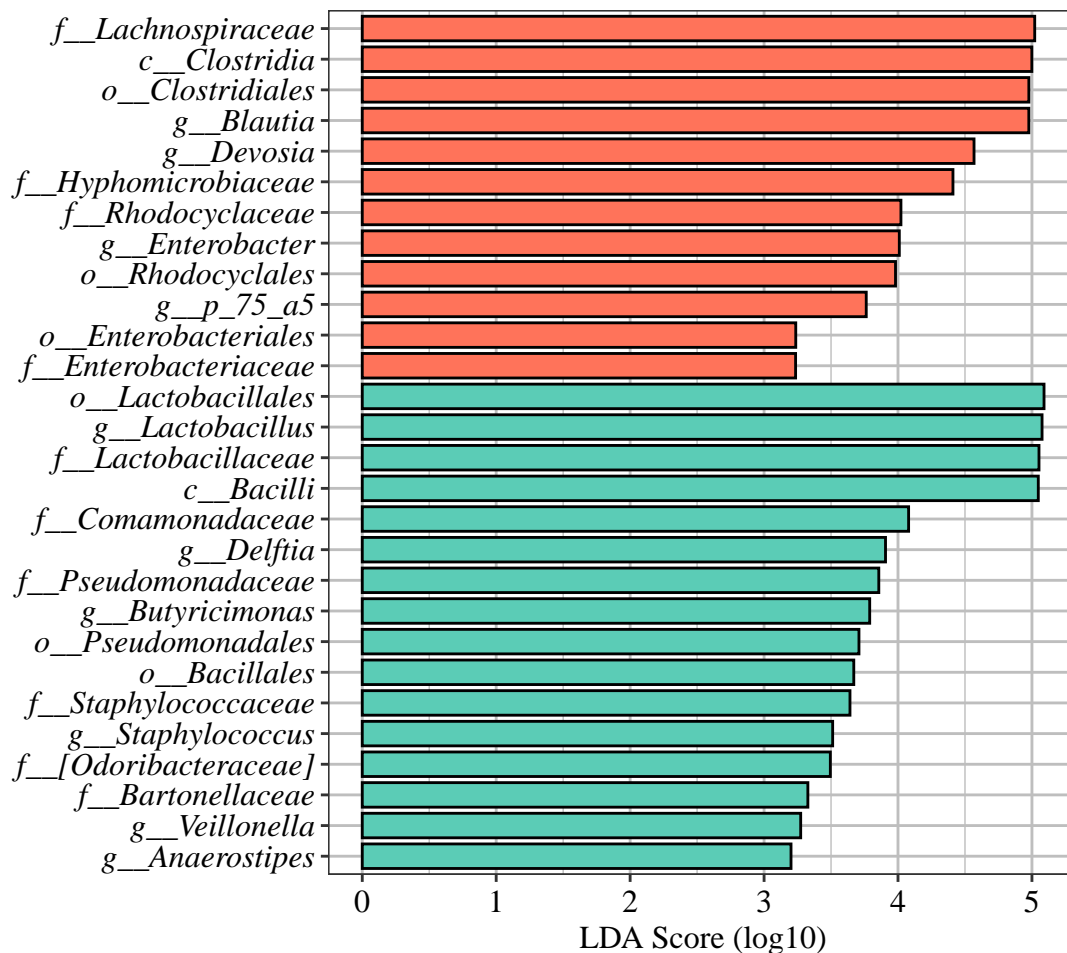

Model  
PAS840

Supplement: Supplementary file 3 [file DataSheet2.zip › Metabolic pathway prediction/lefse_effect_size_rank.pdf]

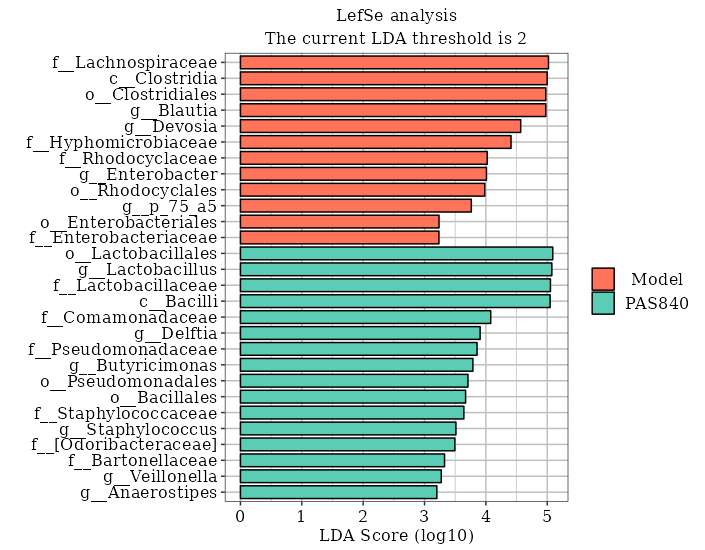

Supplement: Supplementary file 3 [file DataSheet2.zip › Metabolic pathway prediction/lefse_effect_size_rank.png]

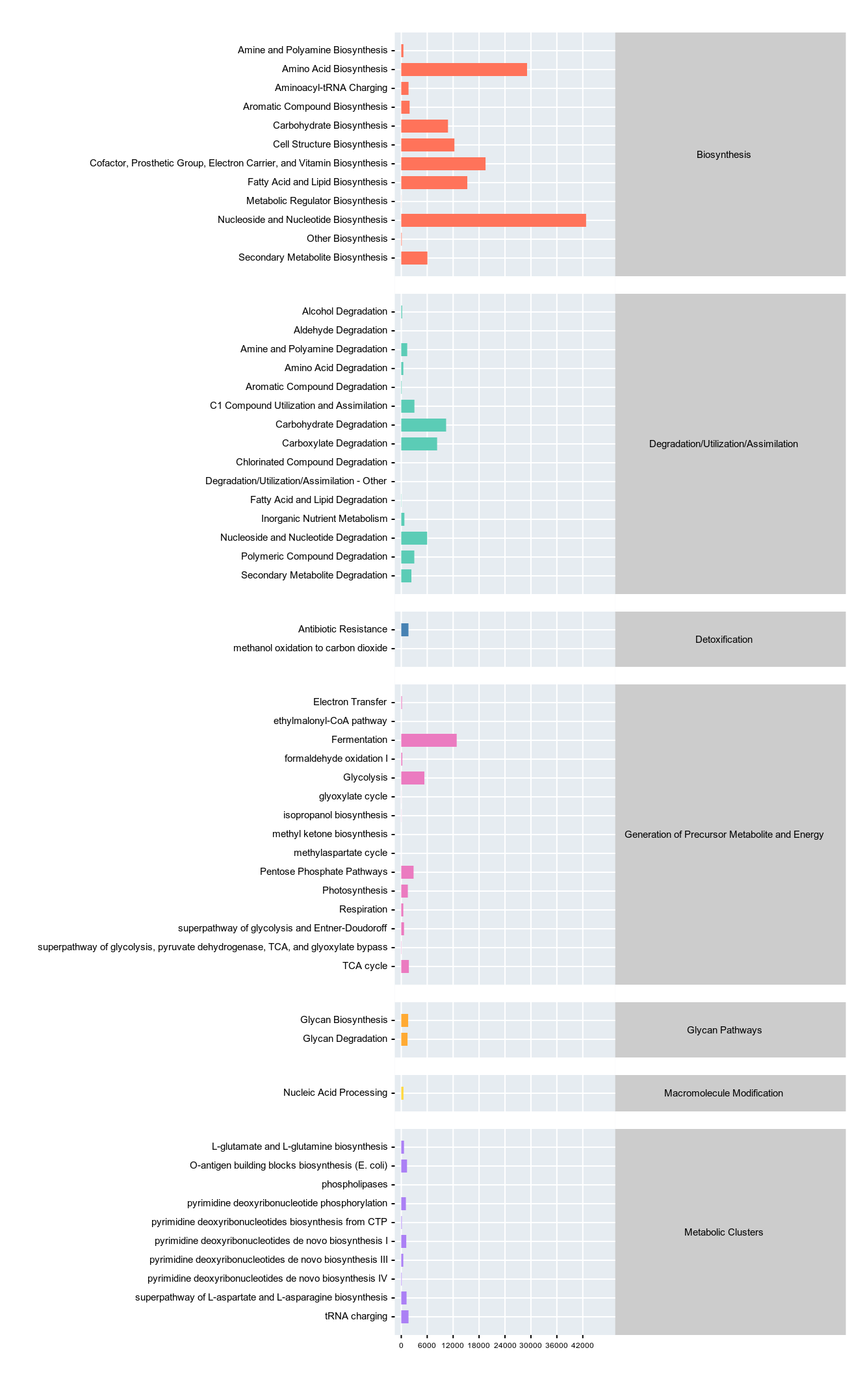

Supplement: Supplementary file 3 [file DataSheet2.zip › Metabolic pathway prediction/MetabolicPathway.png]

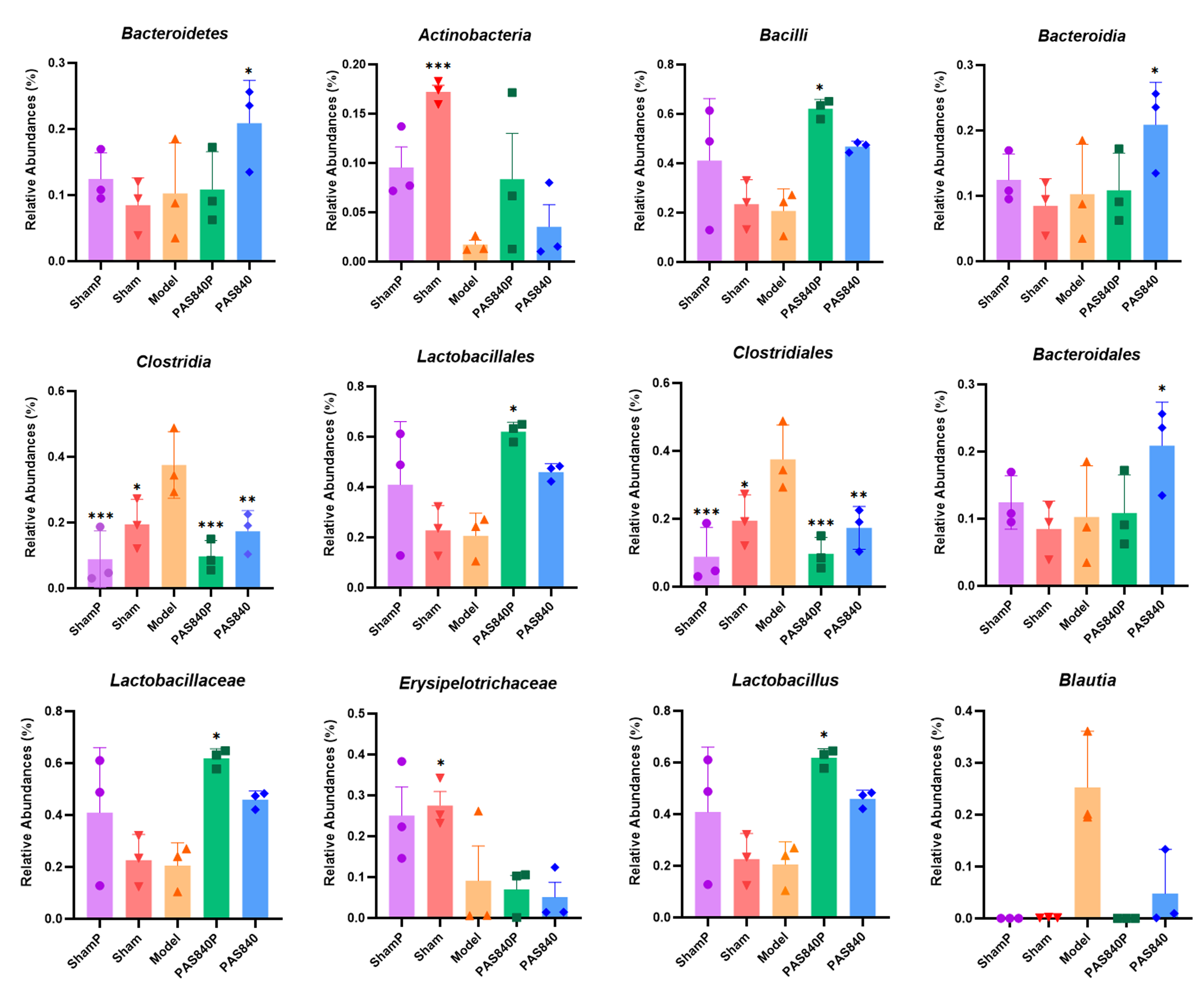

Supplement: Supplementary file 3 [file DataSheet2.zip › Single species analysis/figure 6.png]

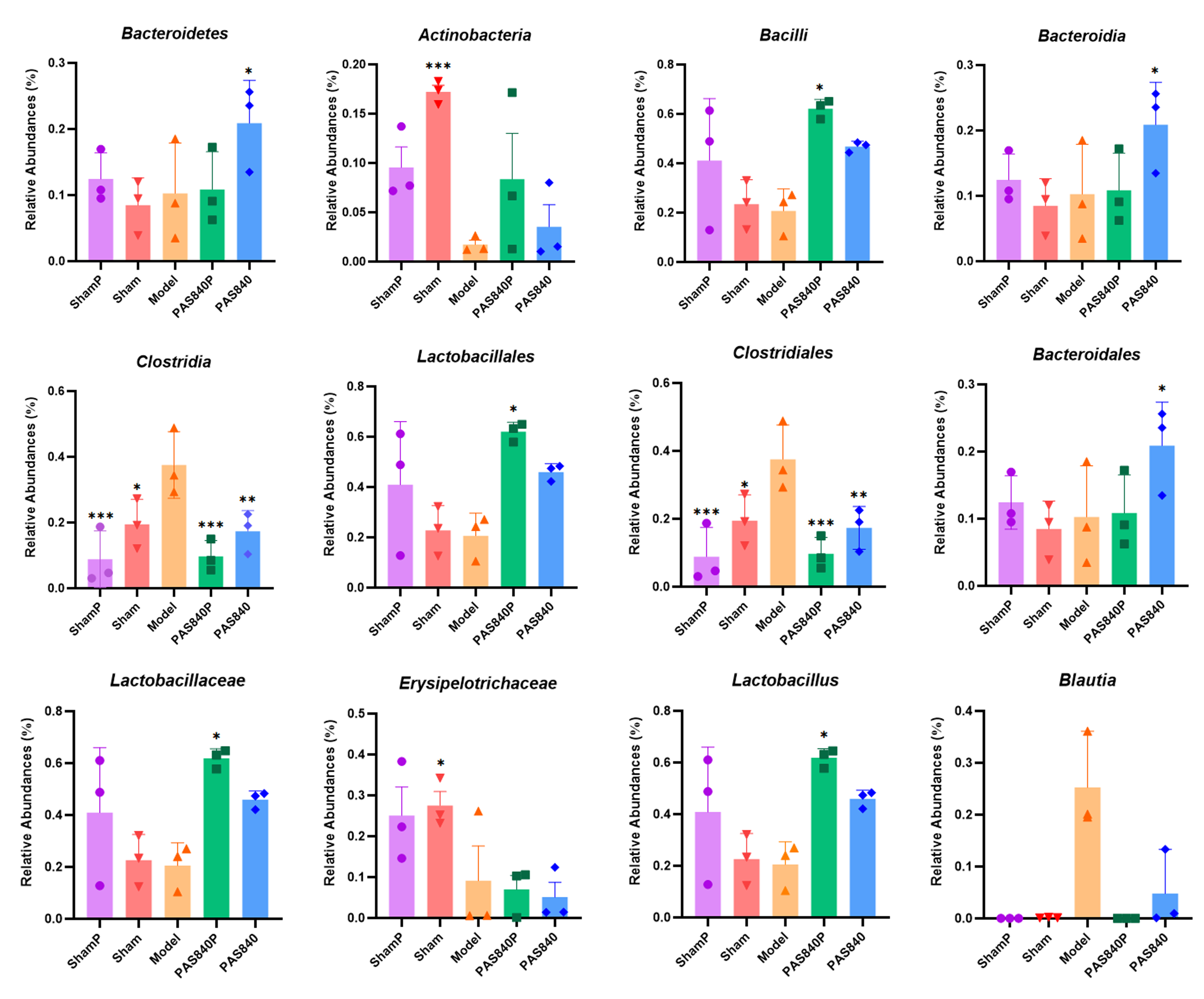

Supplement: Supplementary file 3 [file DataSheet2.zip › Single species analysis/figure 6.tif]

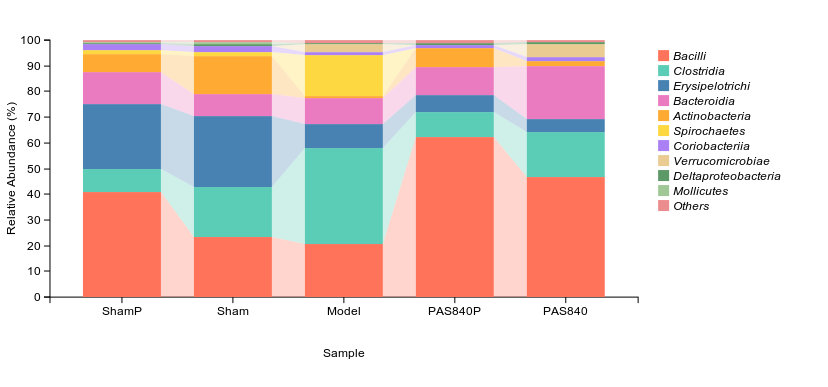

Supplement: Supplementary file 3 [file DataSheet2.zip › Species analysis/Class.png]

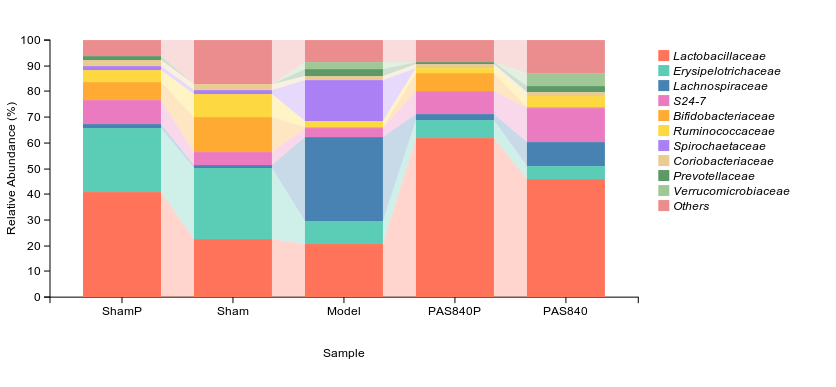

Supplement: Supplementary file 3 [file DataSheet2.zip › Species analysis/Family.png]

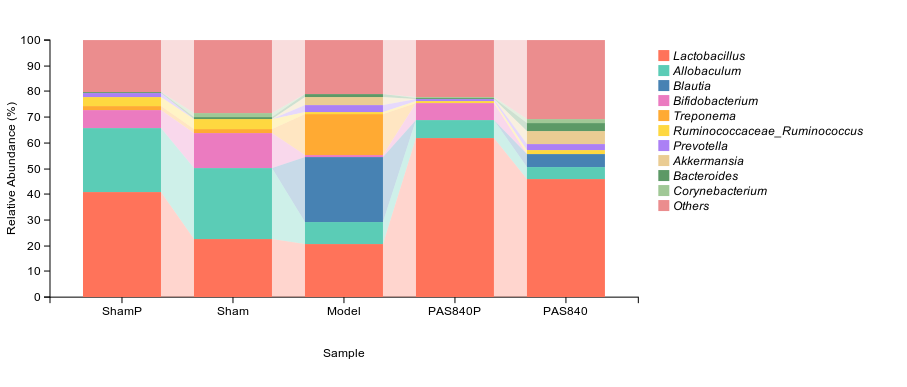

Supplement: Supplementary file 3 [file DataSheet2.zip › Species analysis/Geuns.png]

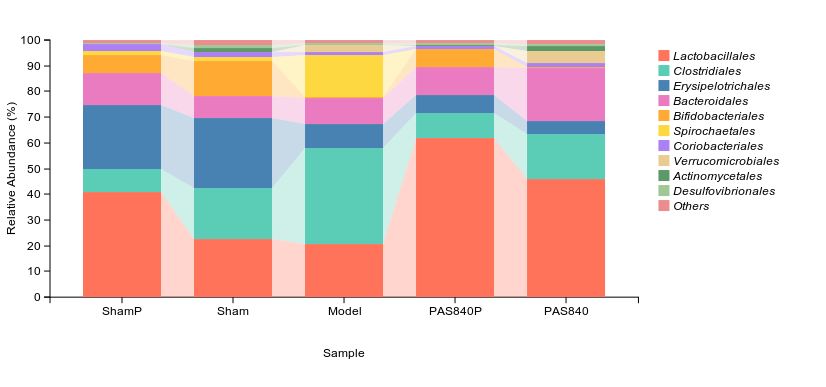

Supplement: Supplementary file 3 [file DataSheet2.zip › Species analysis/Order.png]

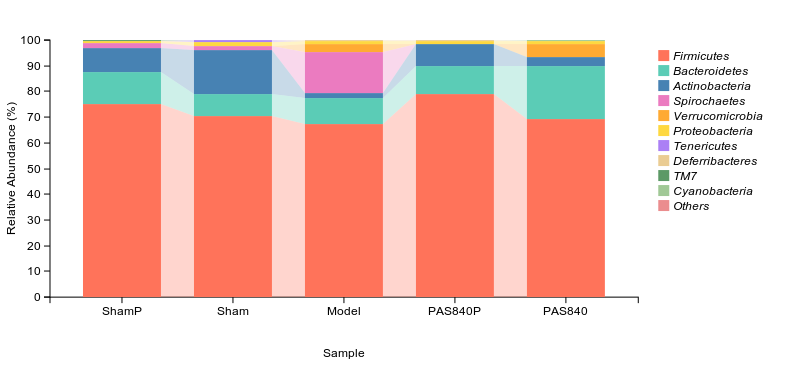

Supplement: Supplementary file 3 [file DataSheet2.zip › Species analysis/Phylum.png]

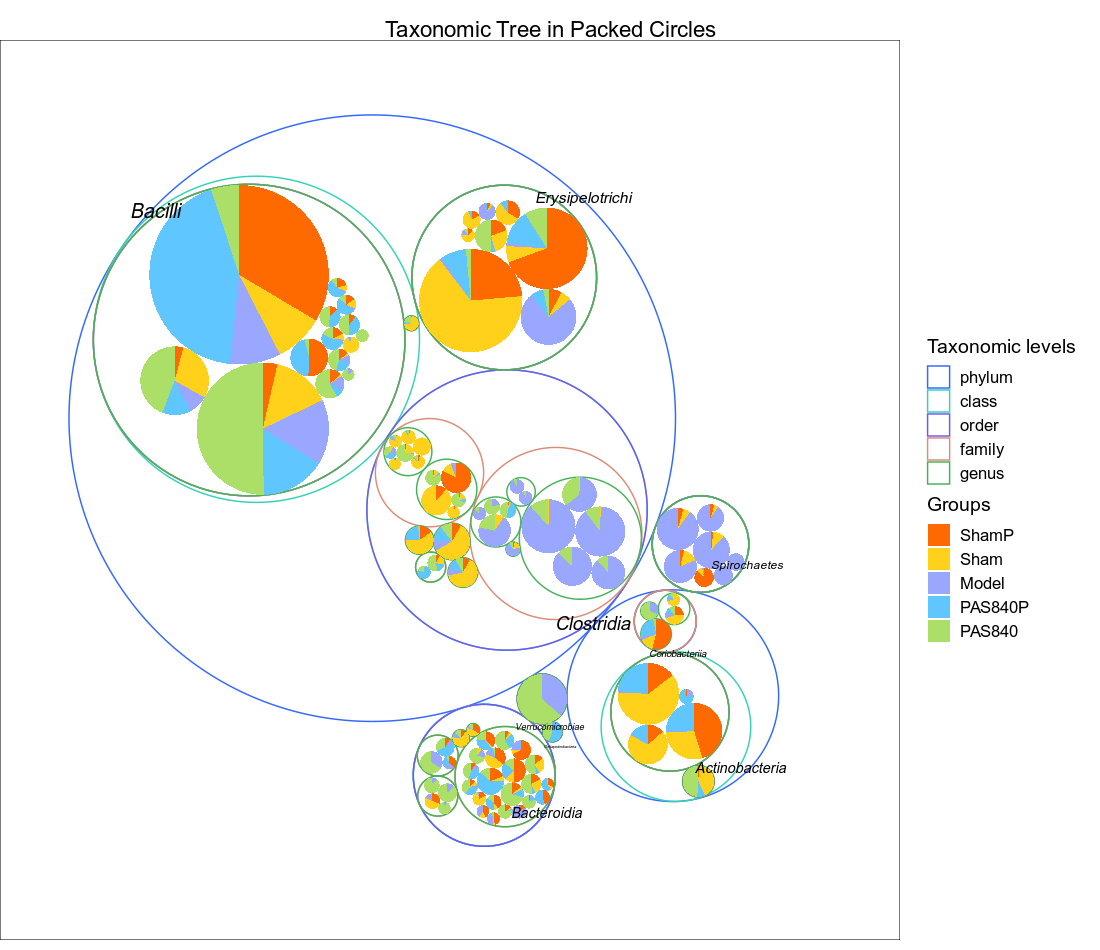

Supplement: Supplementary file 3 [file DataSheet2.zip › Species analysis/taxonomic_tree.png]

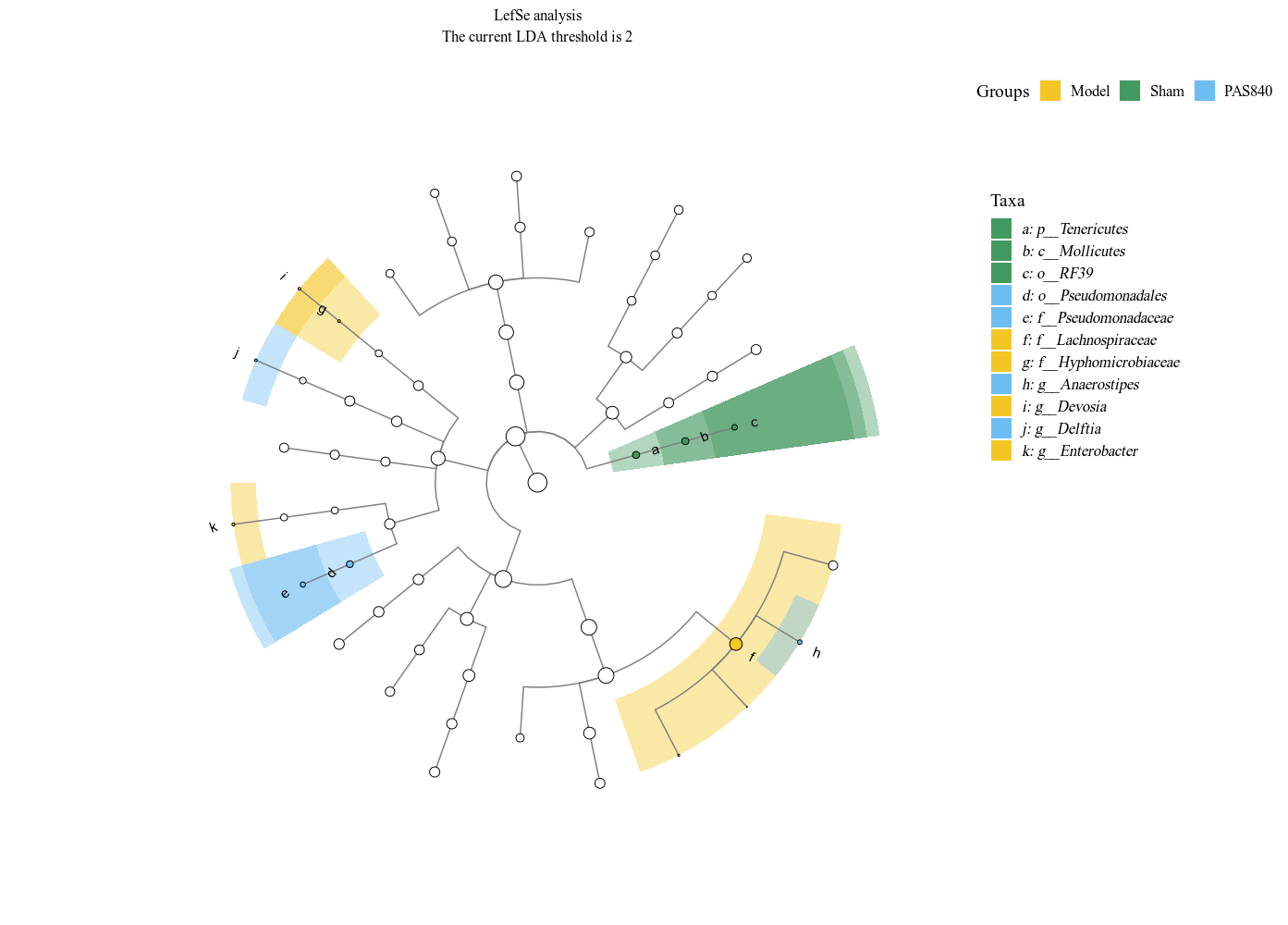

Supplement: Supplementary file 3 [file DataSheet2.zip › Species difference and marker analysis/lefse_cladogram.png]

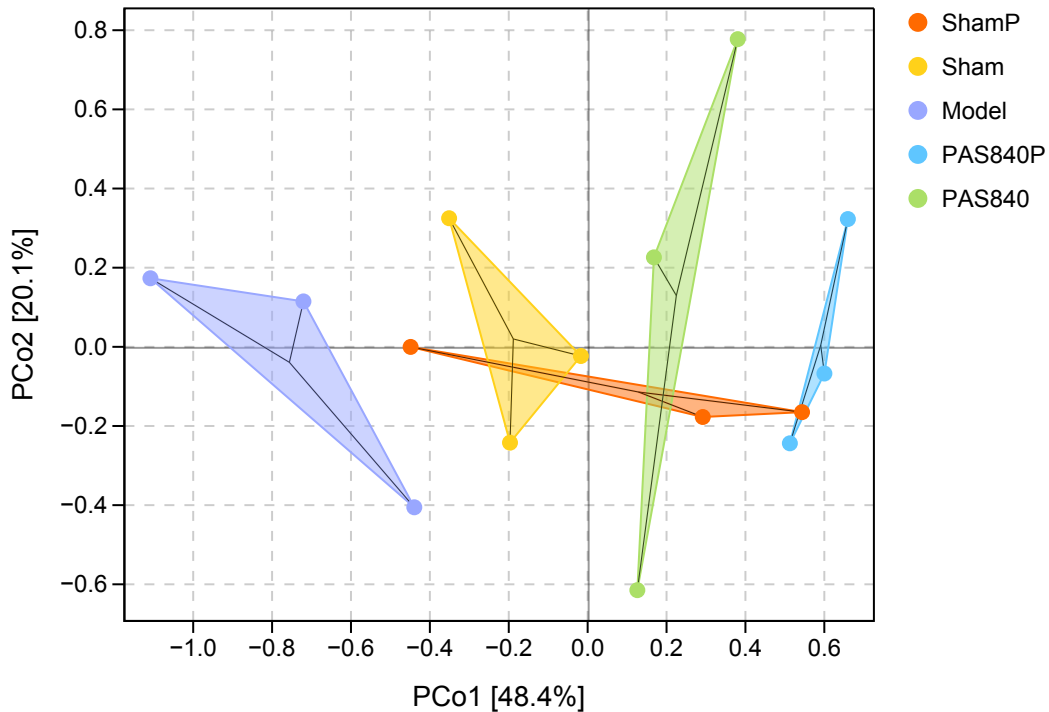

Supplement: Supplementary file 3 [file DataSheet2.zip › Species difference and marker analysis/OPLSDA.pdf]

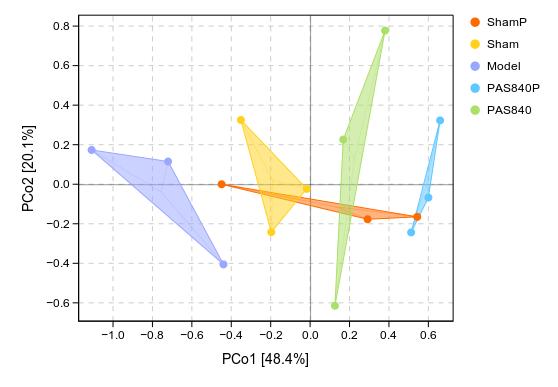

Supplement: Supplementary file 3 [file DataSheet2.zip › Species difference and marker analysis/OPLSDA.png]

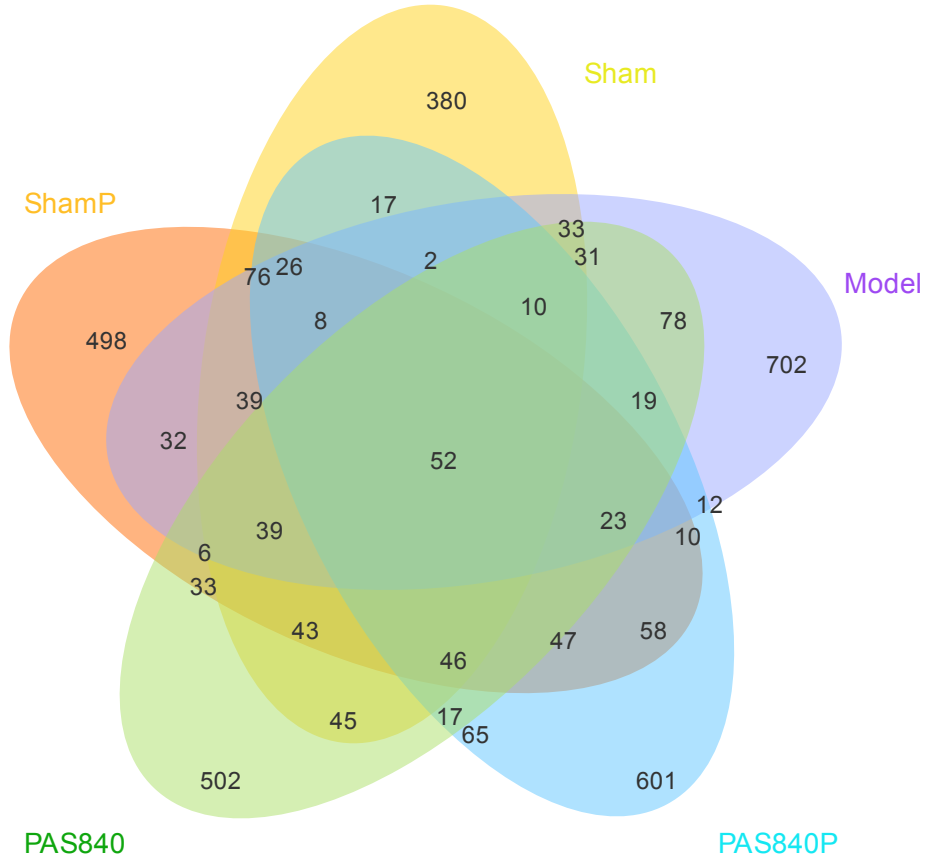

Supplement: Supplementary file 3 [file DataSheet2.zip › Species difference and marker analysis/Venn.pdf]

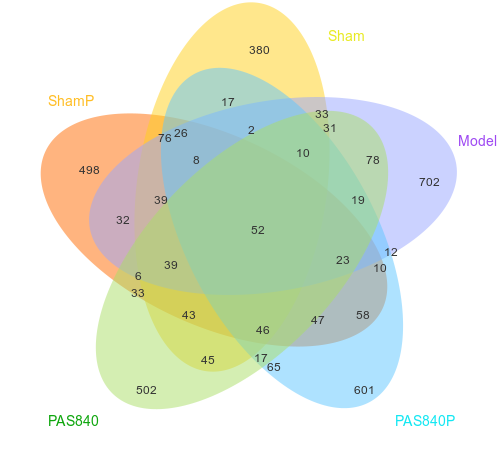

Supplement: Supplementary file 3 [file DataSheet2.zip › Species difference and marker analysis/Venn.png]

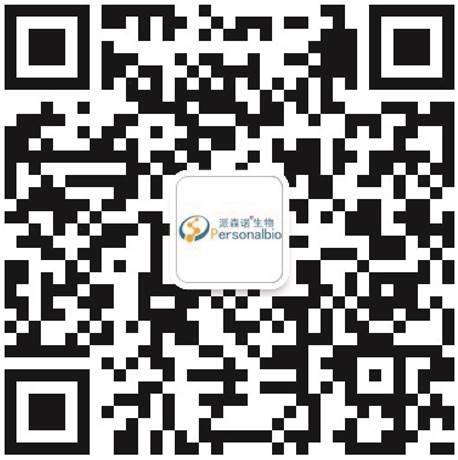

Supplement: Supplementary file 3 [file DataSheet2.zip › static/icon/2Dplot_1.jpg]

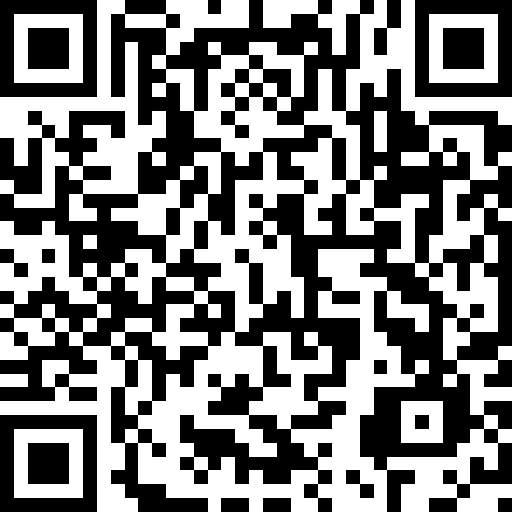

Supplement: Supplementary file 3 [file DataSheet2.zip › static/icon/2Dplot_2.png]

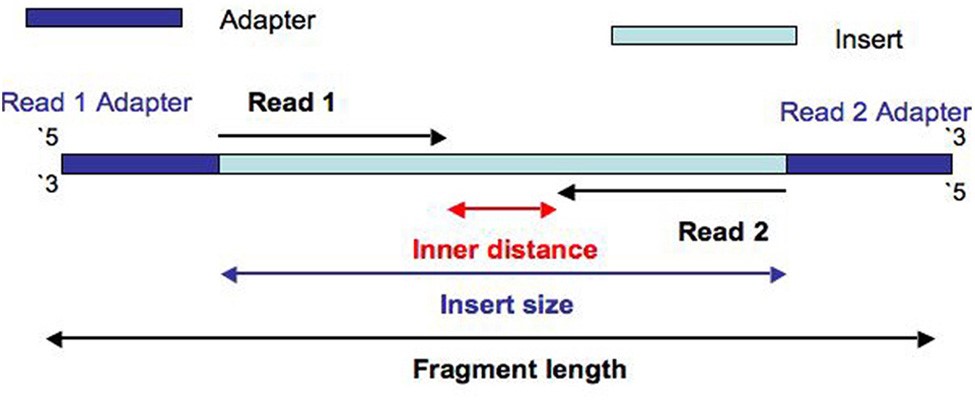

Supplement: Supplementary file 3 [file DataSheet2.zip › static/icon/adapter.jpg]

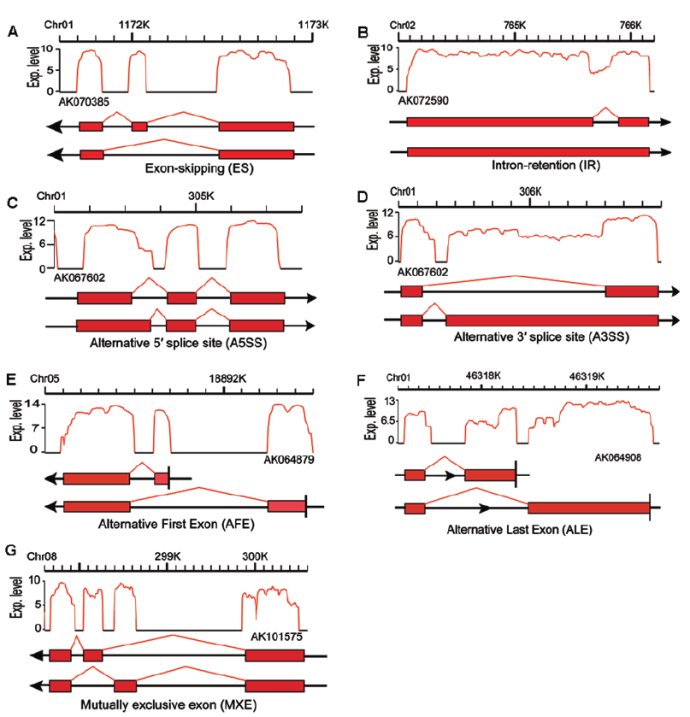

Supplement: Supplementary file 3 [file DataSheet2.zip › static/icon/AS_type.jpg]

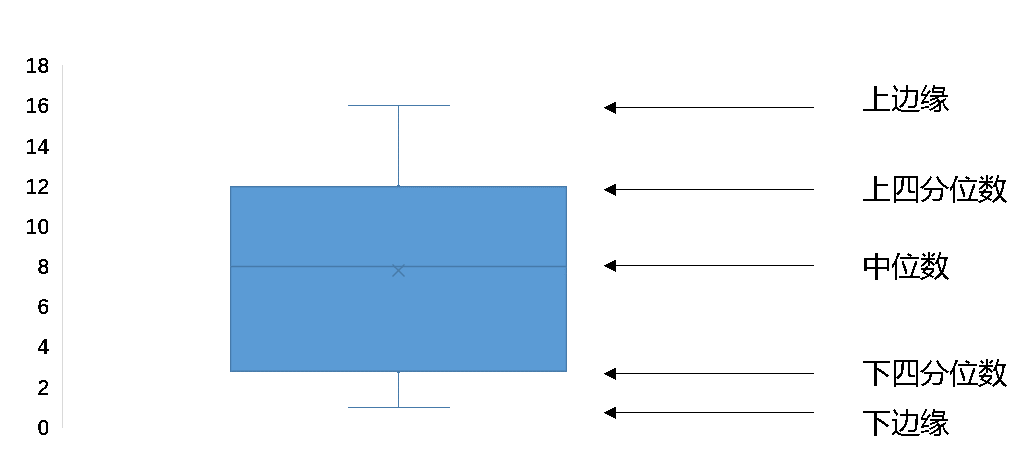

Supplement: Supplementary file 3 [file DataSheet2.zip › static/icon/boxplot_exam.png]

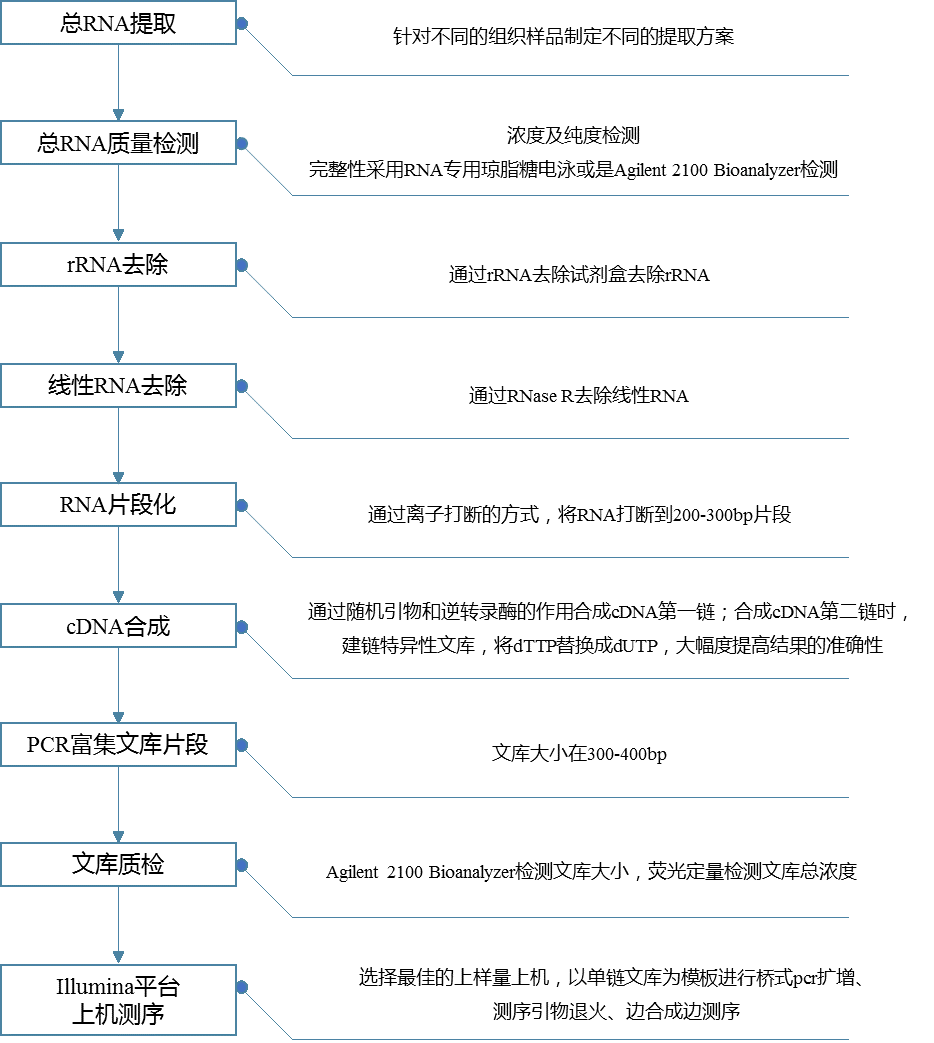

Supplement: Supplementary file 3 [file DataSheet2.zip › static/icon/CircRNA_library.png]

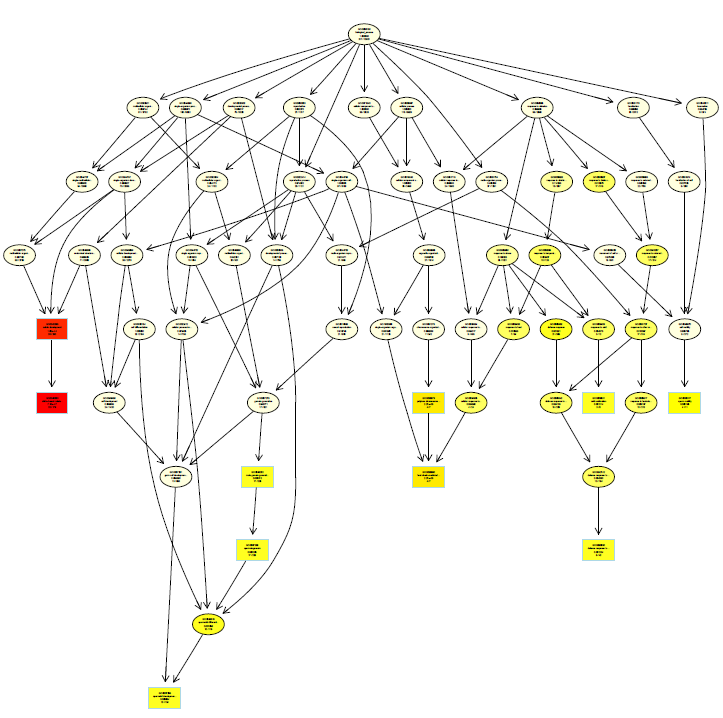

Supplement: Supplementary file 3 [file DataSheet2.zip › static/icon/DAG_exam.png]

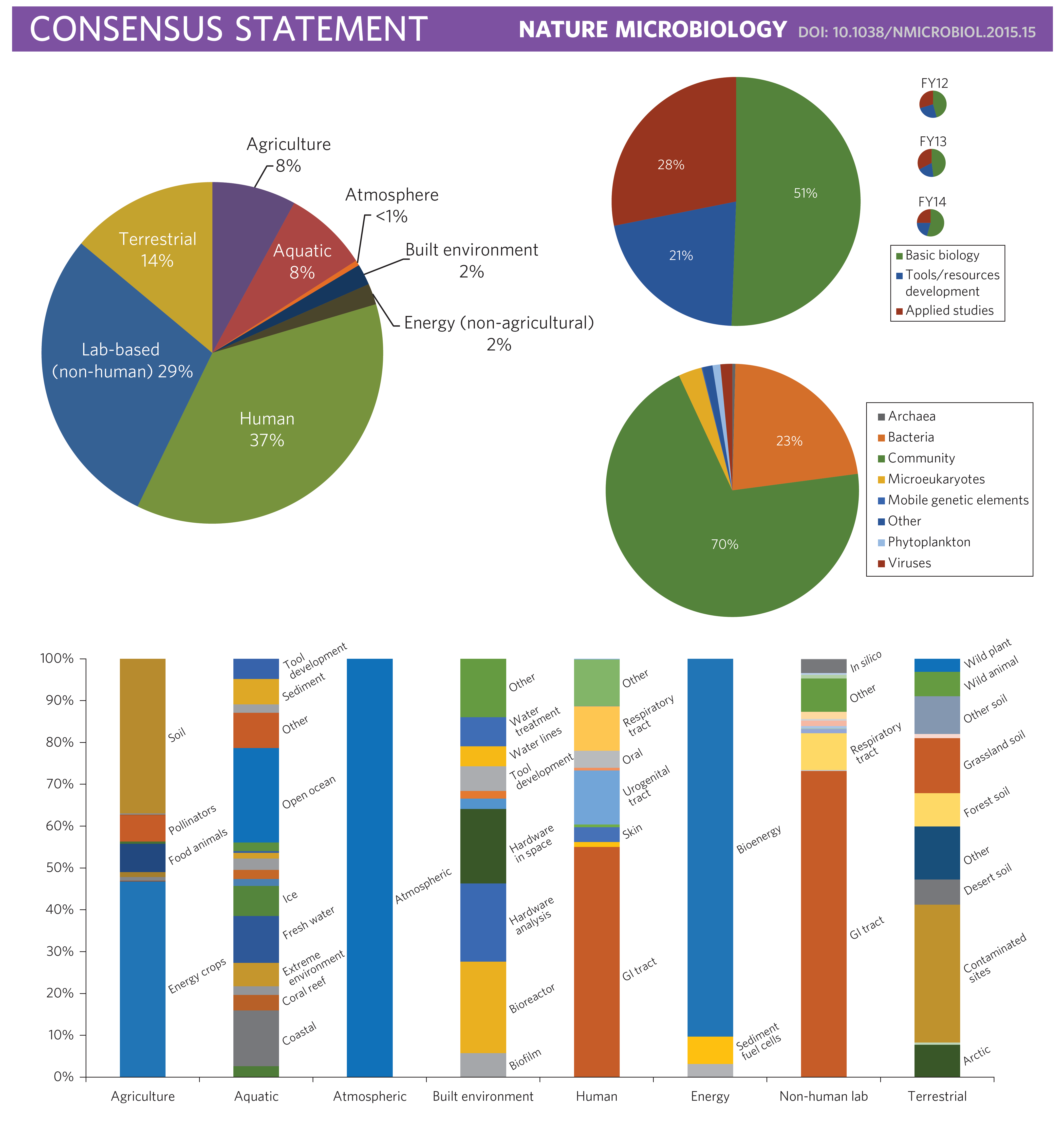

Supplement: Supplementary file 3 [file DataSheet2.zip › static/icon/flow1.png]

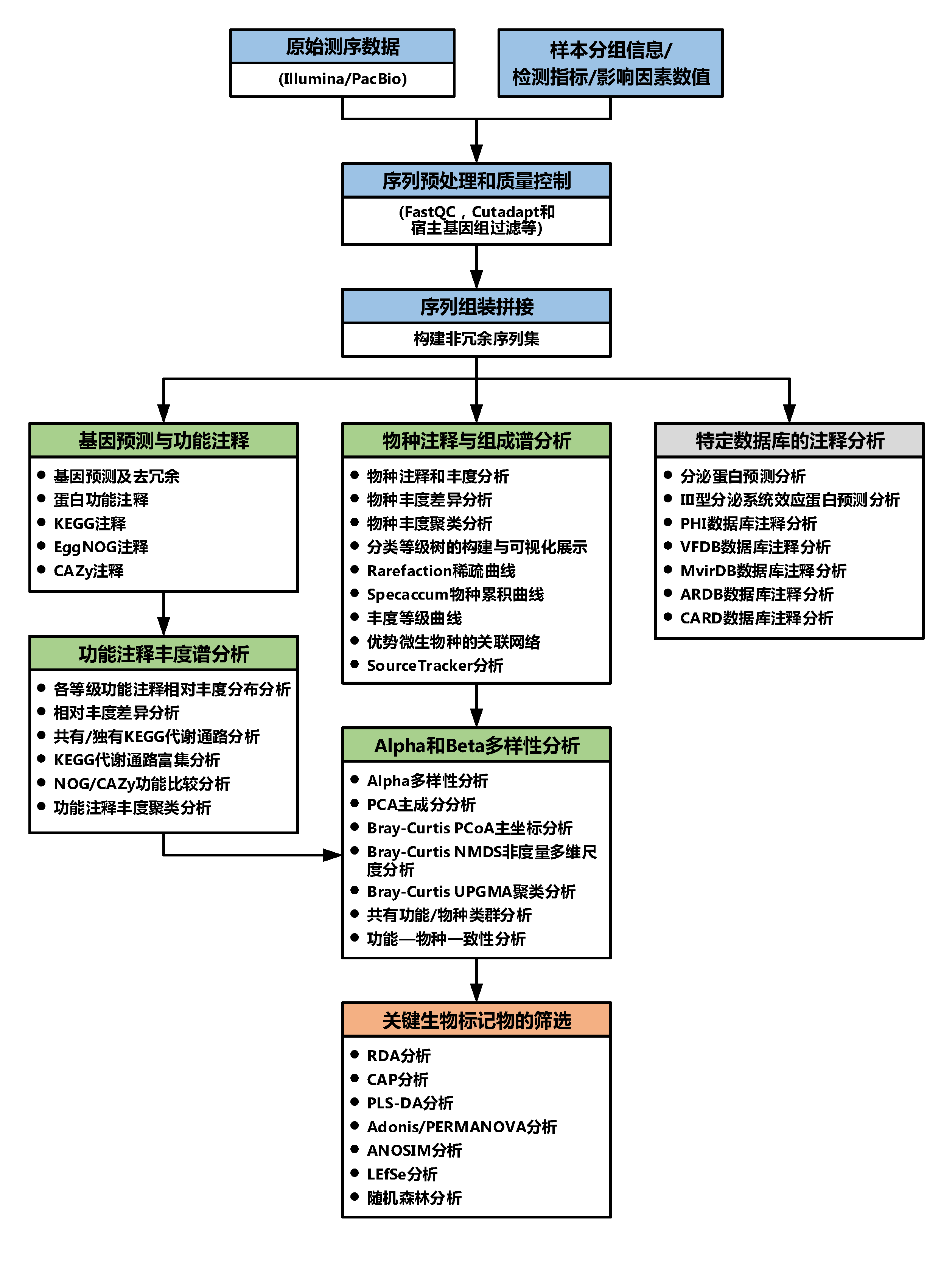

Supplement: Supplementary file 3 [file DataSheet2.zip › static/icon/flow2.png]

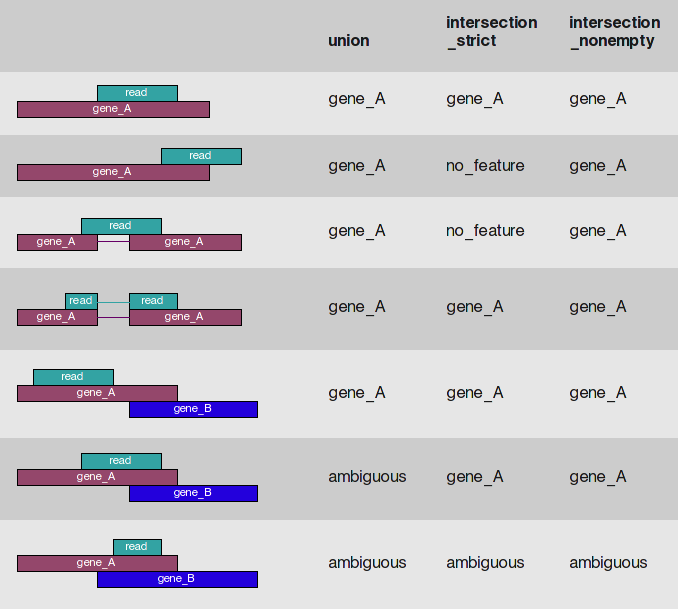

Supplement: Supplementary file 3 [file DataSheet2.zip › static/icon/htseq.png]

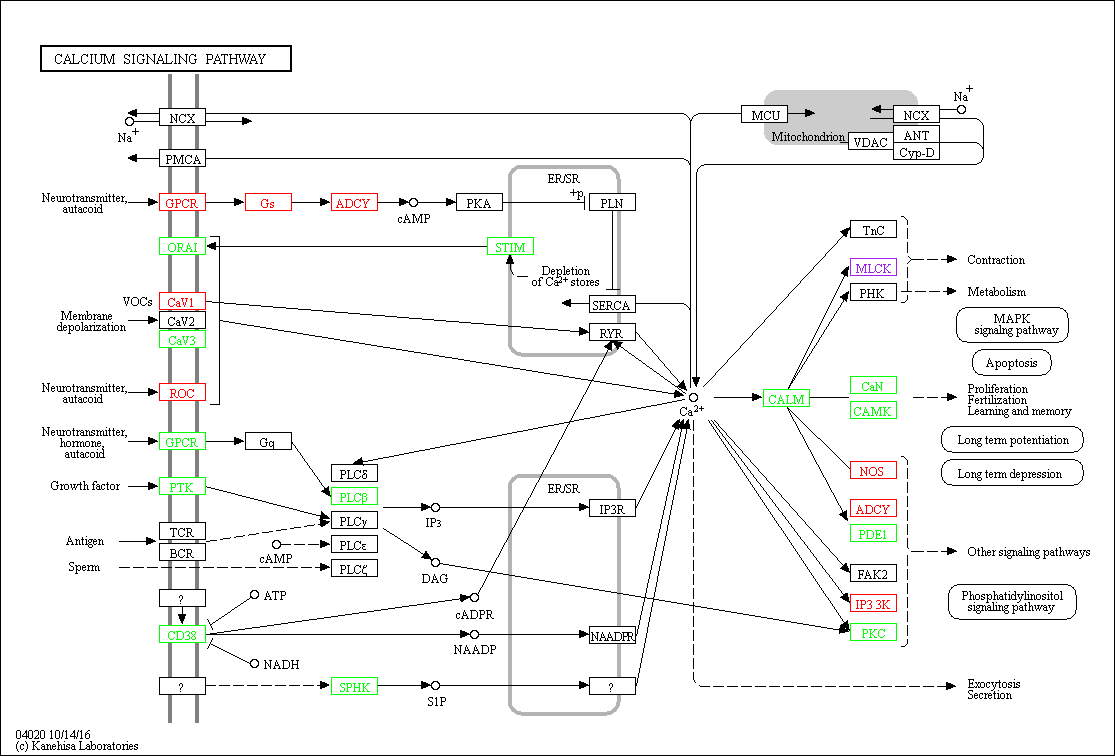

Supplement: Supplementary file 3 [file DataSheet2.zip › static/icon/KO_exam.png]

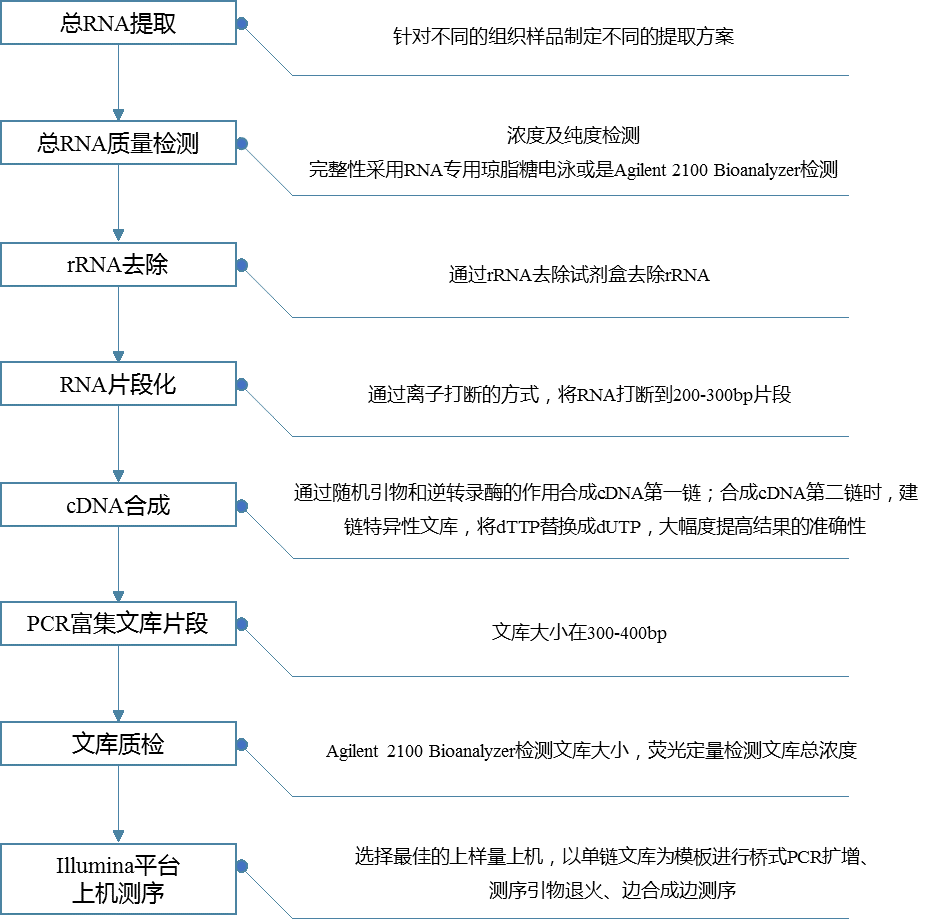

Supplement: Supplementary file 3 [file DataSheet2.zip › static/icon/LncRNA_library.png]

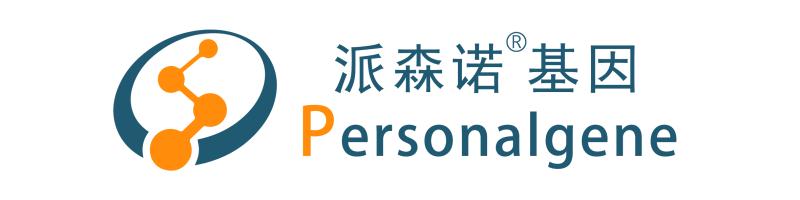

Supplement: Supplementary file 3 [file DataSheet2.zip › static/icon/logo.png]

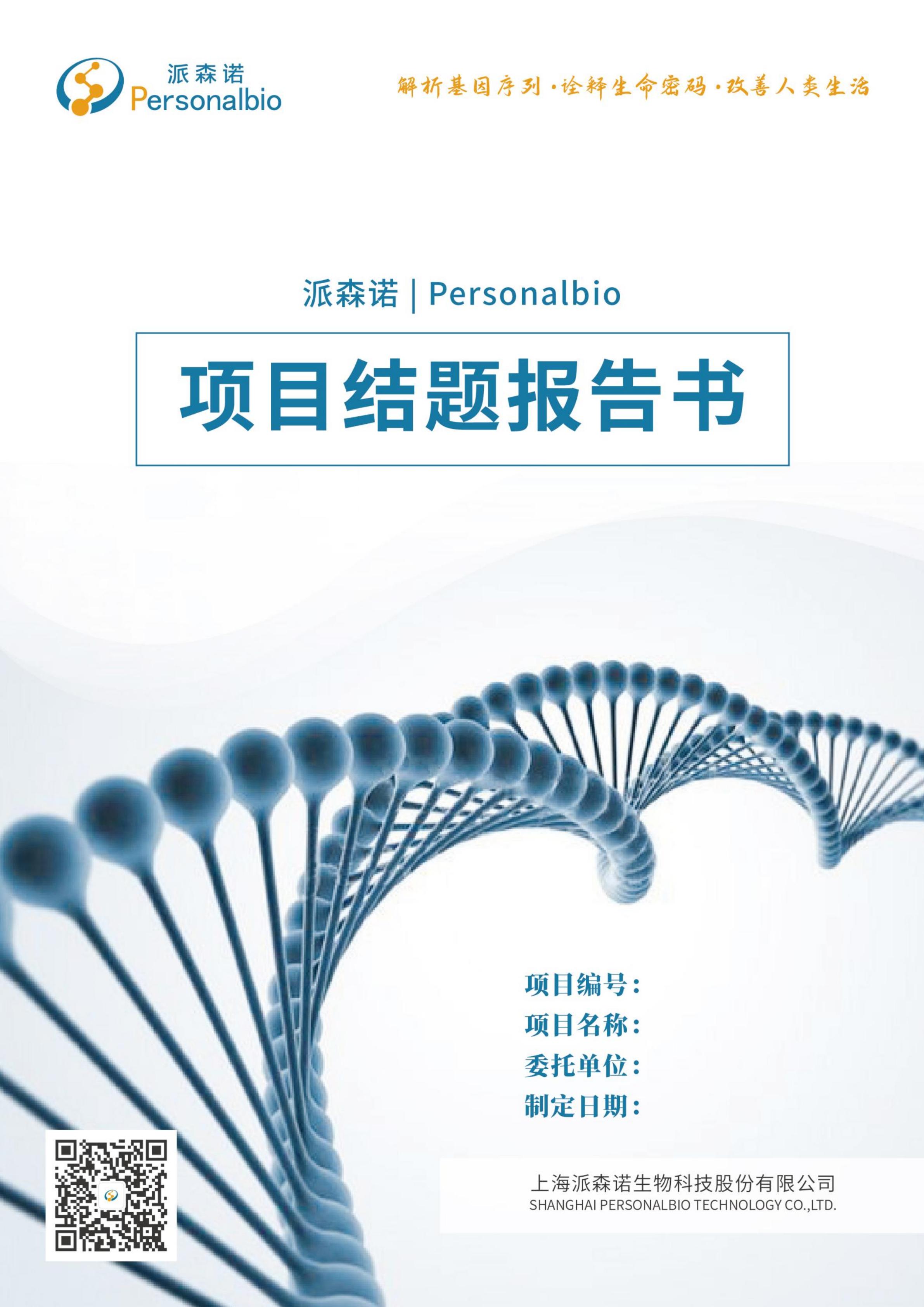

Supplement: Supplementary file 3 [file DataSheet2.zip › static/icon/page_bg.jpg]

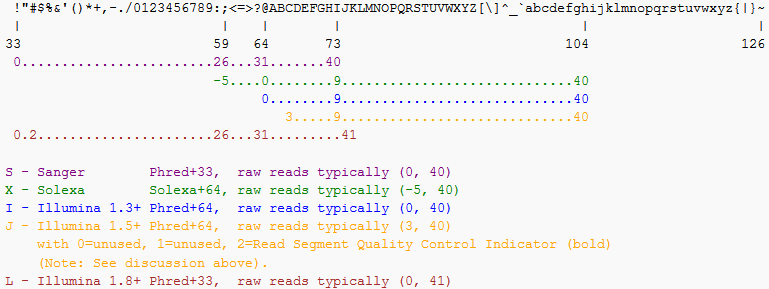

Supplement: Supplementary file 3 [file DataSheet2.zip › static/icon/phred_range.png]

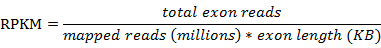

Supplement: Supplementary file 3 [file DataSheet2.zip › static/icon/RPKM_calculation.png]

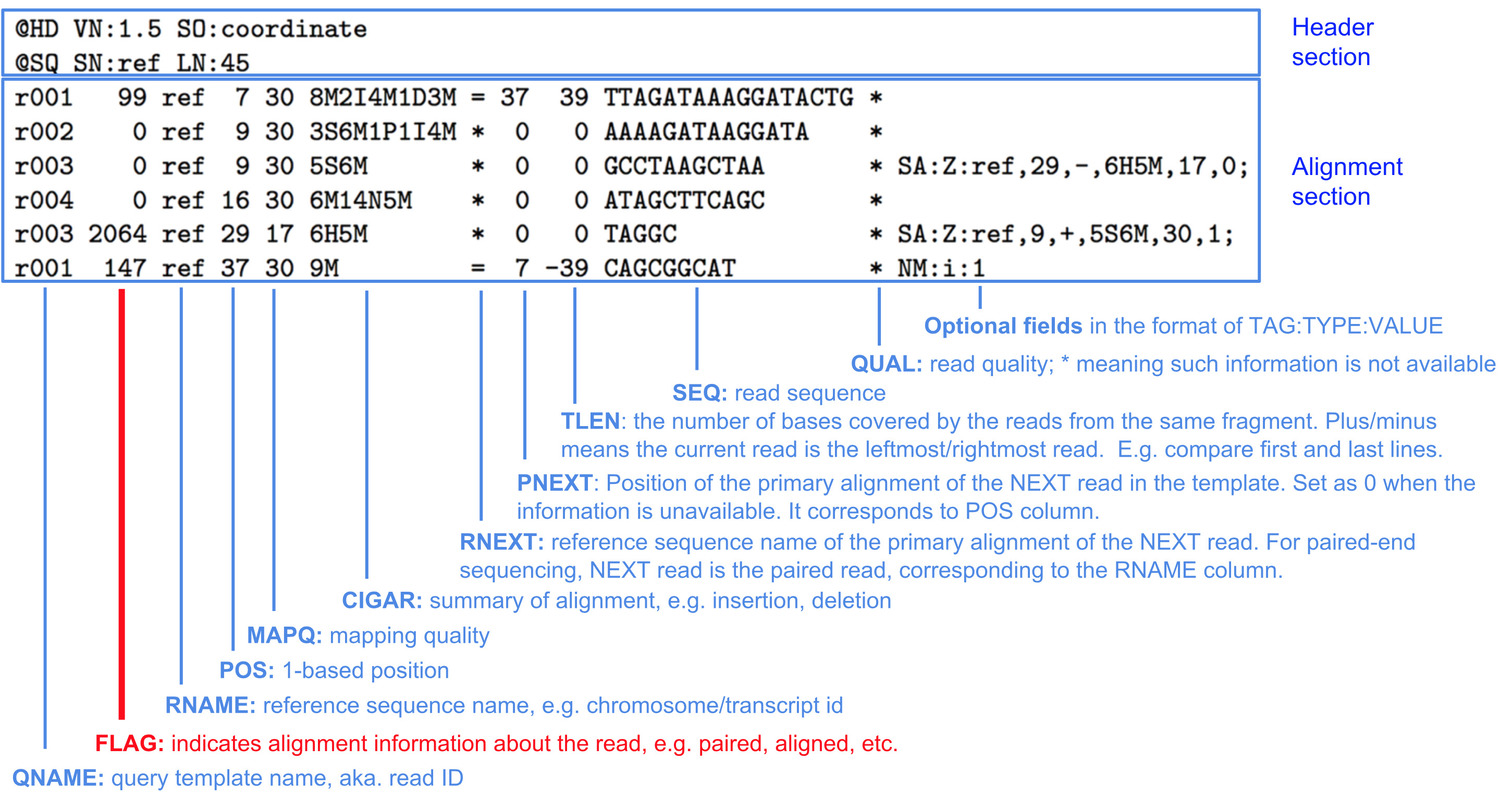

Supplement: Supplementary file 3 [file DataSheet2.zip › static/icon/sam_format.jpg]
