# Supplementary material for: Investigation of the effects of Periplaneta americana (L.) extract on ischemic stroke based on combined multi-omics of gut microbiota
Source: Front Pharmacol. 2024 Nov 28;15:1429960. doi: 10.3389/fphar.2024.1429960 (PMC11638836; doi:10.3389/fphar.2024.1429960)
Supplement: Supplementary file 3 [file DataSheet2.zip › Metabolic pathway prediction/lefse_cladogram.pdf]

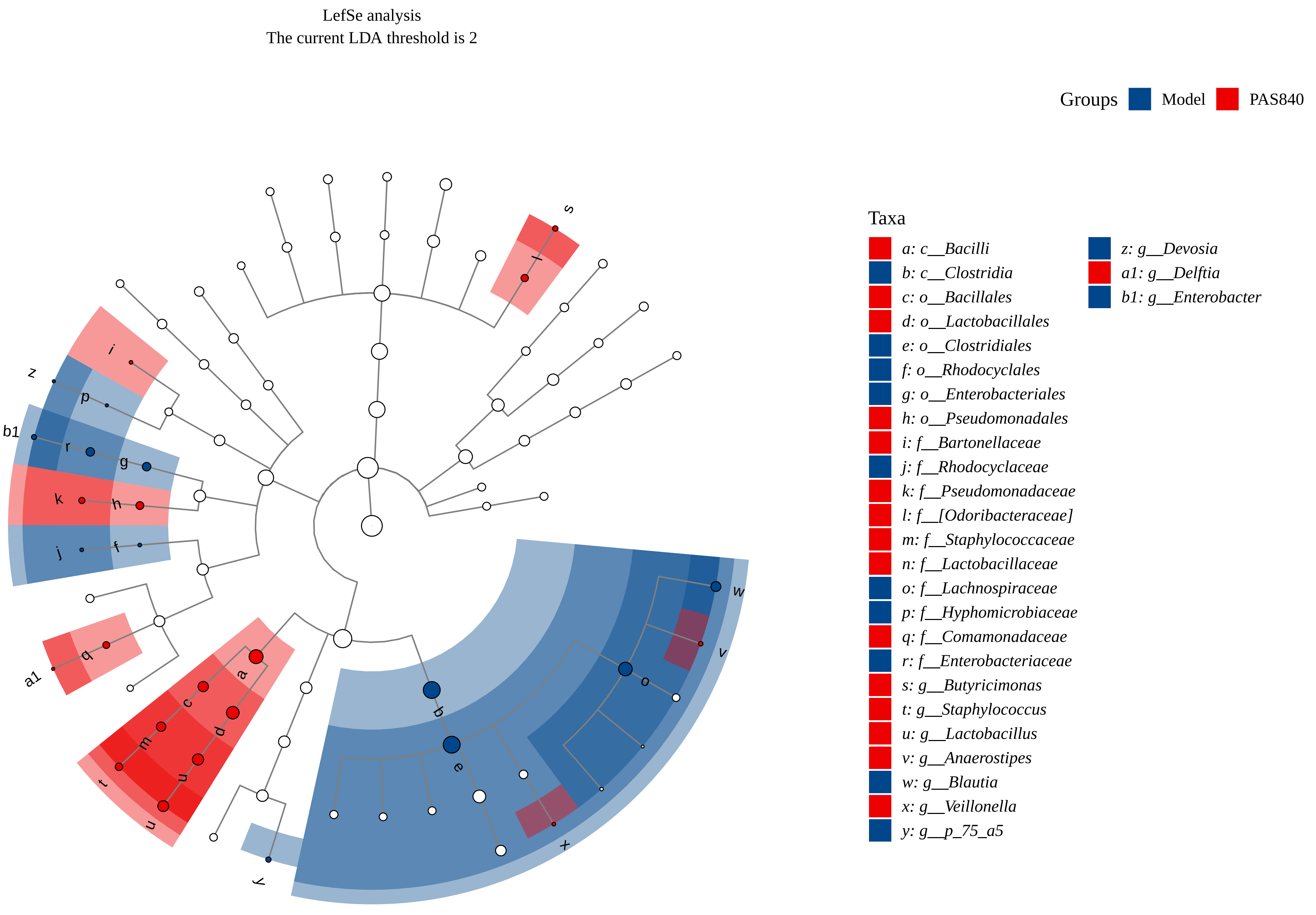

LefSe analysis
The current LDA threshold is 2
Groups
Model
PAS840
Taxa
a: c__Bacilli
b: c__Clostridia
c: o__Bacillales
d: o__Lactobacillales
e: o__Clostridiales
f: o__Rhodocyclales
g: o__Enterobacteriales
h: o__Pseudomonadales
i: f__Bartonellaceae
j: f__Rhodocyclaceae
k: f__Pseudomonadaceae
l: f__[Odoribacteraceae]
m: f__Staphylococcaceae
n: f__Lactobacillaceae
o: f__Lachnospiraceae
p: f__Hyphomicrobiaceae
q: f__Comamonadaceae
r: f__Enterobacteriaceae
s: g__Butyricimonas
t: g__Staphylococcus
u: g__Lactobacillus
v: g__Anaerostipes
w: g__Blautia
x: g__Veillonella
y: g__p_75_a5
z: g__Devosia
a1: g__Delftia
b1: g__Enterobacter
