# Supplementary material for: Investigation of the effects of Periplaneta americana (L.) extract on ischemic stroke based on combined multi-omics of gut microbiota
Source: Front Pharmacol. 2024 Nov 28;15:1429960. doi: 10.3389/fphar.2024.1429960 (PMC11638836; doi:10.3389/fphar.2024.1429960)

Taxonomic Tree in Packed Circles

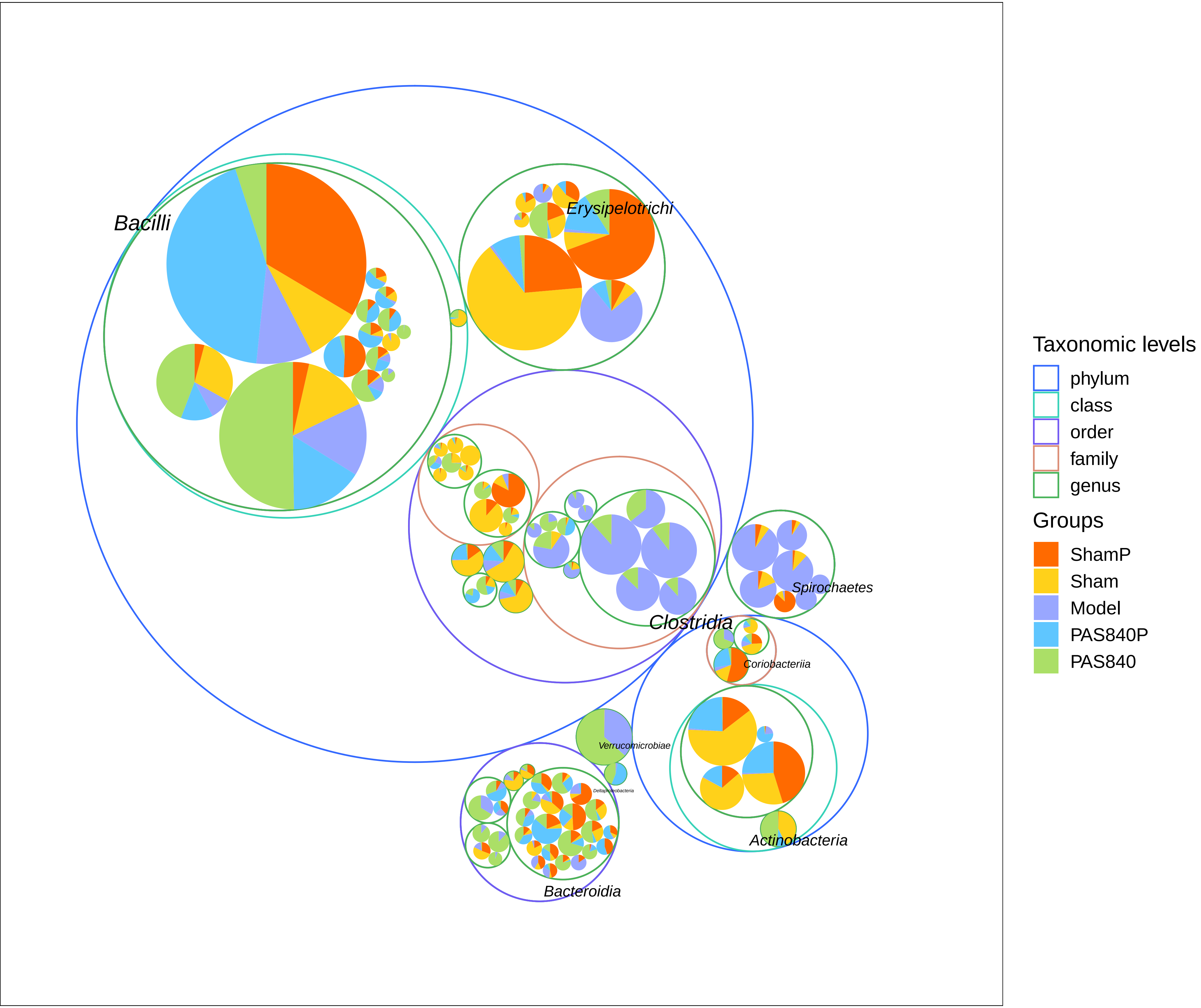

Supplement: Supplementary file 3 [file DataSheet2.zip › Species analysis/taxonomic_tree.pdf]
